# ARNTL-mediated INO80-DHX15 axis reprograms the glycolytic metabolism and augments the progression of endometrial carcinoma

**DOI:** 10.1038/s41419-025-07776-w

**Published:** 2025-06-20

**Authors:** Lei Ye, Genyi Jiang, Yihan Sun, Bilan Li

**Affiliations:** Shanghai Key Laboratory of Maternal Fetal Medicine, Shanghai Institute of Maternal-Fetal Medicine and Gynecologic Oncology, Shanghai First Maternity and Infant Hospital, School of Medicine, Tongji University, Shanghai, PR China

**Keywords:** Cancer metabolism, Cancer metabolism

## Abstract

Immune evasion is a major mechanism responsible for tumor cell survival and dissemination. This study aims to explore key molecules involved in immunosuppression and metastasis of endometrial carcinoma (EC). Primary and metastatic tumors were collected from four patients with EC for array analysis. Metastatic tumors exhibited increased macrophage infiltration, while decreased CD8^+^ T cell infiltration, and aryl hydrocarbon receptor nuclear translocator-like (ARNTL) was identified as a key factor involved. High ARNTL expression was linked to poor tumor differentiation, advanced stage, and increased metastasis in another cohort of 300 EC patients. ARNTL knockout (ARNTL-KO) in EC cells reduced cell proliferation, migration, and invasion, and increased cell death in vitro, and it blocked the tumorigenicity and metastatic activity of cells in mice. The ARNTL-KO EC cells reduced the M2 polarization of macrophages and induced CD8^+^ T cell proliferation both in vitro and in vivo. ARNTL activated the transcription of INO80 complex ATPase subunit (INO80), a chromatin remodeler, which further promoted the transcription of DEAH-box helicase 15 (DHX15) through histone acetylation modifications. Overexpression of either INO80 or DHX15 increased glycolytic activity and immunosuppression in ARNTL-KO EC cells. Collectively, this study suggests that the ARNTL-mediated INO80-DHX15 axis induces glycolysis and immunosuppression during EC progression.

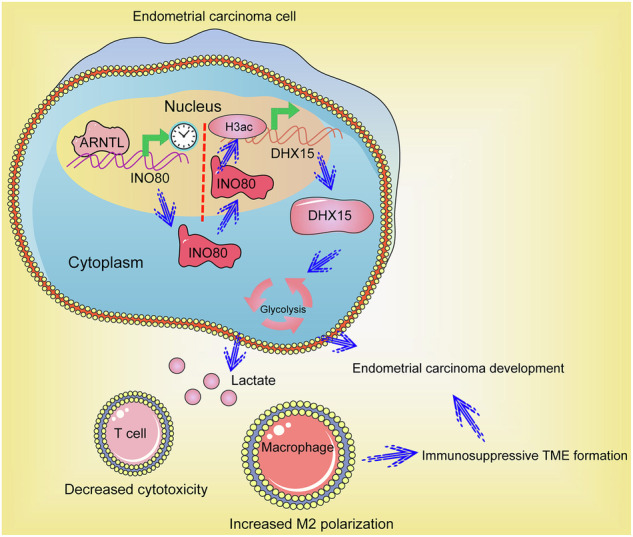

## Introduction

Cancer of the corpus uteri, which is usually referred to as endometrial carcinoma (EC), is defined as a malignancy resident in the inner epithelial lining of the uterus with rising incidence and mortality rates across the world [1]. With an estimated 420,242 new cases and 97,704 disease-associated deaths worldwide, EC represents the sixth most common cancer in women in 2022 [2]. The overall prognosis is relatively good, though high-grade ECs are predisposed to recur and disseminate [3]. The five-year survival rate for patients diagnosed is approximately 96% at stage I and between 80–90% at stage II, while the survival rate significantly declined to 20-25% in metastatic EC [4]. There remain serious challenges in the understanding of EC biology and the identification of key causative factors implicated in metastatic diseases.

Anti-tumor immune responses involve various cell types and multiple steps and rely on the cooperation between innate and adaptive immune systems [5]. It has been recognized that the presence of T cells in tumor lesions, especially CD8^+^ cytotoxicity T cells, is linked to cancer cell elimination and better prognosis of cancer patients [6]. The presence of programmed death 1 (PD-1) on T cells and programmed cell death ligand 1 (PDL-1) on tumor cells represents the major mechanism responsible for T cell dysfunction and exhaustion [7]. Tumor-associated macrophages (TAMs) are another major population of immune cells in the tumor microenvironment (TME) whose infiltration was, however, correlated with therapy resistance and poor prognosis [8]. According to the distinct stimulating cytokines, TAMs can be roughly categorized into two forms: the classically activated (M1) type that exerts pro-inflammatory and anti-tumoral functions and the alternatively activated (M2) type that gives rise to anti-inflammatory and pro-tumoral phenotype [9]. Specifically, the M2-skewed macrophages, which take the predominance in TAMs, can suppress the activity of CD8^+^ T cells by releasing transforming growth factor-β (TGF-β) and interleukin-10 (IL-10) [10]. The interplay of TAMs and CD8^+^ T cells is therefore crucial for tumor development.

In the present study, by conducting clinical sample-based array analysis and integrated bioinformatics analyses, we obtained aryl hydrocarbon receptor nuclear translocator-like (ARNTL) as a key regulator linked to differential infiltration of macrophages and CD8^+^ T cells in metastatic EC samples. ARNTL, also known as BMAL1, is a member of the bHLH-PAS transcription factor family that can govern the cellular circadian rhythm [11]. A disruption of the circadian clock has been correlated with tumorigenesis [12], the remodeling of TME, and increased tumor cell proliferation [13], which is also true for the development of EC [14]. Aberrant ARNTL expression has been associated with immune cell infiltration and malignant phenotype of several human malignancies, such as acute myeloid leukemia [15] and glioblastoma [16]. However, the regulatory role of ARNTL in EC progression, especially in immune cell infiltration, has not been defined.

The additional bioinformatics insights suggested the INO80 complex ATPase subunit (INO80) as a promising downstream molecule of ARNTL. INO80 is a complex, multi-subunit protein that plays a critical role in chromatin remodeling, primarily involved in the repositioning of nucleosomes [17]. It modulates histone acetylation and accessibility of target gene promoters to modulate glycolysis and respiration, thereby maintaining metabolic stability [18]. Currently, it is widely accepted that glycolysis is the primary energy provider in EC cells, facilitating the metabolic adaptability necessary for tumor growth, invasion, and metastasis [19]. Interestingly, aberrant aerobic glycolysis also provides an immunosuppressive niche through several mechanisms and therefore inhibits the anti-tumor immune response and further promotes malignant cell development [20]. However, the function of INO80 in glycolysis and immune response in EC remains unclear. Furthermore, the bioinformatics predictions revealed DEAH-box helicase 15 (DHX15) as a potential glycolysis-related gene regulated by INO80. DHX15 is an ATP-dependent RNA helicase that unwinds RNA structures and is involved in the splicing of pre-mRNA, ribosome biogenesis, DHX15, and RNA decay processes [21]. Interestingly, DHX15 has been identified as a critical regulator of glycolysis in mosquito cells [22]. Still, the functions of DHX15 in the glycolytic process and immune activity in tumors are elusive. Collectively, this study was conducted to verify the interactions between ARNTL, INO80, and DHX15 and delve into their biological functions in the reprogramming of glycolytic metabolism and immune response during EC development.

## Materials and methods

### Clinical samples

Primary EC tumor tissues were surgically obtained from 300 patients treated at Shanghai First Maternity and Infant Hospital from March 2010 to December 2020. These cases encompassed 259 instances of endometrioid carcinoma, 23 cases of serous carcinoma, two cases of clear cell carcinoma, two cases of mixed carcinoma, two cases of undifferentiated carcinoma, and 12 cases of carcinosarcoma. In addition, four pairs of in situ and metastatic tumors were obtained from another four patients diagnosed with metastatic disease; all cases were of endometrioid carcinoma with wild-type p53 and ER/PR positivity. All patients were diagnosed with EC for the first time, and none had any other malignancies. Before surgery, no patient had undergone anti-cancer chemo-radiotherapy. Comprehensive pathological data were available for all patients. Additionally, the study included 10 healthy individuals from whom peripheral blood samples were collected for peripheral blood mononuclear cell (PBMC) collection. The study protocol was approved by the Institutional Review Board of Shanghai First Maternity and Infant Hospital (approval number: KS23332). All included respondents signed the informed consent. All experiments involving human samples were conducted following the *Declaration of Helsinki*.

### Expression profiling by array analysis

Total RNA from the four pairs of in situ and metastatic tumor samples was extracted using the total RNA extractor (TRIzol; Sangon Biotech Co., Ltd., Shanghai, China). Double-strand cDNA was synthesized by chimeric oligonucleotides, oligo-dT, and T7RNA polymerase. The GeneChip Eukaryotic Poly-A RNA Control Kit (900433, Thermo Fisher Scientific, Rockford, IL, USA) was used to monitor the processes of cRNA amplification and labeling, and the exogenous positive control was added to the total RNA before cDNA synthesis. In short, the cRNA was amplified and purified and then biotin-labeled using the GeneChip 3’ IVT PLUS Reagent Kit (902416, Thermo Fisher Scientific). After that, 20 μg biotin-labeled cRNA was added to the cRNA fragmentation buffer and heated at 94 °C for 35 min, followed by hybridization with the GeneChip Human Genome U133A 2.0 Array (900471, Thermo Fisher Scientific) at 45 °C for 1 h. The human genome expression data were obtained by the GeneChip Scanner 3000 7 G Whole-Genome Association System (00-0362, Thermo Fisher Scientific).

### Immunological niche analysis of metastatic tumors

The array analysis data were used to analyze the fractions of infiltrating stromal/immune cells in in-situ and metastatic tumor samples, respectively. The Estimation of STromal and Immune cells in MAlignant Tumors using Expression data (ESTIMATE) R package (version 1.0.13) [23] was applied to analyze the stromal, immune, and ESTIMATE scores in each tumor sample based on the expression of specific genes. The three scores were positively correlated with the stromal cells, immune cells, and the sum of these two. Namely, a higher score indicates a higher fraction of the corresponding cell types in the tumor samples. The Immuno-Oncology Biological Research (IOBR) R package (version 0.99.9) [24] and the deconvo_Cell-type Identification By Estimating Relative Subsets Of RNA Transcript (CIBERSORT) method [25] were used to analyze the infiltration scores of various types of immune cells according to gene expression profiles in the tumor samples of each patient.

To identify the immune-related signaling pathways, we downloaded the immunologic signature gene sets from the Molecular Signatures Database v7.5 (https://software.broadinstitute.org/cancer/software/gsea/wiki/index.php/MSigDB_v7.5.1_Release_Notes) and analyzed the differentially enriched immunologic signature gene sets between the in-situ and metastatic tumor samples using the gene set variation analysis (GSVA) R package (version 3.16) [26].

### Immunohistochemistry (IHC)

Paraffin-embedded tumor tissue sections were dewaxed, rehydrated, soaked in citrate buffer (pH 6.0) for heat-mediated antigen retrieval, incubated in 3% H_2_O_2_ to block the activity of endogenous peroxidase, and incubated with goat serum to block non-specific binding. The sections were incubated with the following diluted primary antibodies overnight at 4 °C: ARNTL [1:50, 14268-1-AP, Proteintech (PTGCN) Group, Inc., Wuhan, Hubei, China], Ki67 (1:1000, 27309-1-AP, PTGCN), INO80 (1:200, 18810-1-AP, PTGCN), PDL-1 (1:500, ab205921, Abcam, Cambridge, MA, USA), and interferon-gamma (IFN-γ, 1:500, ab231036, Abcam). On the next day, the sections were incubated with HRP-conjugated goat anti-rabbit (H + L) (1:50, A0208, Beyotime Biotechnology Co., Ltd, Shanghai, China) at 22–25 °C for 1 h. After color development by DAB and counter-staining by hematoxylin, the sections were observed under microscopy to analyze positive (brownish) staining. Quantitative analysis of positively stained areas was performed by researchers who were unaware of the grouping.

### Multi-label immunofluorescence staining of tissue microarrays

We performed tissue microarray detection on immune cell markers to analyze the infiltration of immune cells in tumors from 300 patients with EC. The multi-label immunofluorescence staining of tissue microarrays was entrusted to Beijing Aibixin Biotechnology Co., Ltd (Beijing, China). The macrophage marker CD68 and T cell markers CD8 (Opal 620), CD28 (Opal 570), and CD80 (Opal 690) were analyzed.

### Cell culture and artificial gene intervention

Human endometrial epithelial cells (EECs, CP-H058), human in-situ EC cell lines HEC-1A (CL-0099) and RL95-2 (CL-0197) derived from primary tumors, and a human EC cell line AN3CA (CL-0505) derived from metastatic tumors were procured from Procell Life Science & Technology Co., Ltd (Wuhan, Hubei, China) and cultured at 37 °C with 5% CO_2_ in minimal-essential medium (MEM) along with 10% fetal bovine serum (FBS) and 1% antibiotics. Another EC cell line, EFE-184 (CBP61196), derived from metastatic tumors, was provided by Nanjing Cobioer Biosciences Co., Ltd (Nanjing, Jiangsu, China). This cell line was cultured in RPMI-1640 supplemented with 10% FBS. All cell lines were authenticated by short tandem repeat profiling and confirmed free of microbial contamination, such as mycoplasma.

Mammal-based CRISPR lentivirus vectors (single gRNAs) for ARNTL knockout (ARNTL-KO), lentiviral vectors containing overexpression plasmid of INO80 (Vector-INO80) and DHX15 (Vector-DHX15), short hairpin RNA (shRNA) of INO80 (sh-INO80), and the negative control (NC) vectors (Vector-NC and sh-NC). The gRNA sequences target Exon 13 of ARNTL (NM_001351804.1). The target sequence of gRNA1# was GCAGTCGTCCAATTGCGACG, and that of gRNA2# was GTTTCTCGGCACGCGATAGA. The target sequence of sh-INO80 was TCTATATGAACAGGGTATTAA. The multiplicity of infection was 10 for cell infection. Cells with stable ARNTL knockout or INO80 knockdown were screened by puromycin, and those with stable INO80 and DHX15 overexpression were screened by G-418.

### Reverse transcription-quantitative polymerase chain reaction (RT-qPCR)

Total RNA from cells or xenograft tumors (see details in the text later) was extracted using the TRIeasy Total RNA Extraction Reagent (Yeasen Biotechnology Co., Ltd). The optical density (OD) values at 260 and 280 nm were detected, and the A260/280 ratio was calculated to evaluate RNA purity. The RNA was reverse-transcribed to cDNA using the First Strand cDNA Synthesis Kit (Roche, Merck KGaA, Darmstadt, Germany), and the qPCR was performed using KiCqStart Probe qPCR ReadyMix (Sigma-Aldrich, Merck KGaA) on the ABI7500 Fast system. Gene expression values relative to the endogenous loading β-actin were evaluated by the 2^−ΔΔCt^ method. The primer sequences are listed in Table S1.

### Cell counting kit-8 (CCK-8) method

The treated EC cells were seeded into 96-well plates (3000 cells/well) and treated with 10 μL of CCK-8 reagent (CA1210, Solarbio Science & Technology Co., Ltd, Beijing, China) at 0, 24, 48, and 72 h, respectively. After another 1.5 h of incubation, the OD450 values were examined to analyze the proliferation of cells.

### Cell death detection

Apoptosis and necrosis of cells were analyzed using the YO-PRO-1/Propidium Iodide (PI) detection kit (C1075S, Beyotime). The cells were seeded into 96-well plates at 5000 cells per well. Each well was then added with 100 μL of YO-PRO-1/PI working solution for 10 min of dark incubation at 37 °C. The staining was observed under fluorescence microscopy. The binding of YO-PRO-1 with the DNA of apoptotic or necrotic cells produces bright green fluorescence, while the binding of PI with the nucleic acid of necrotic cells produces red fluorescence. Positively stained cells were calculated.

### Isolation and culture of immune cells

PBMCs were separated from the blood samples of healthy individuals using the Ficoll-Paque PLUS density gradient media (Cytiva/Global Life Sciences Solutions, Shanghai, China). The PBMCs were cultured in Roswell Park Memorial Institute (RPMI)-1640 complete medium (Thermo Fisher Scientific) containing 40 ng/mL of macrophage-colony stimulating factor and 20 ng/mL of IL-4 for 6 d to differentiate into macrophages. The cells were then harvested for subsequent analyses. CD8^+^ T cells in the untreated PBMCs were isolated by the Dynabeads CD8 Positive Isolation Kit (11333D, Thermo Fisher Scientific), which was expanded and activated using the Dynabeads Human T-Activator CD3/CD28 Kit (11131D, Thermo Fisher Scientific).

Another set of Transwell plates (24-well plates; transparent polyester membrane; 3.0 μm) was applied for the co-culture of macrophages and cancer cells. In short, approximately 3 × 10^4^ macrophages were seeded into the apical chambers, while 6 × 10^4^ EC cells were seeded into the basolateral chambers. The co-culture system was maintained at 37 °C with 5% CO_2_ for 24 h. For T cell treatment, the prepared CD8^+^ T cells were added to the culture system of cancer cells at a ratio of 2:1. After 24 h, the culture solution containing suspended CD8^+^ T cells was collected.

### Transwell migration and invasion assays

Transwell plates (24-well plates with a diameter of 8 μm) were used to analyze the migration and invasion of cells. In short, EC cells (1 × 10^6^) in 200 μL of FBS-free medium were seeded into the apical chambers, and 600 μL of complete medium containing 10% FBS was added to the basolateral chambers. After incubation at 37 °C with 5% CO_2_ for 12 h, the cells migrated to the lower surface and were fixed with 4% paraformaldehyde and stained with 0.1% crystal violet for 30 min. Migratory cells in four random fields under microscopy were counted. The invasion of cells was detected similarly with the additional pre-coating of Matrigel on the apical chambers.

### Flow cytometry

The single cell suspension (100 µL; 5 × 10^6^ cells/mL) was pre-incubated with 5 µL of Human TruStain FcX (Fc Receptor Blocking Solution) (#422301, Biolegend, San Diego, CA, USA) at 22–25 °C for 10 min to rule out the non-specific staining, followed by the addition of fluorophore-conjugated primary antibodies (diluted at 1:100) for dark incubation on ice for 20 min and flow cytometry analysis. The antibodies used included CD206-phycoerythrin (PE) (#321105, Biolegend), CD8-PE (#980902, Biolegend), and CD68-fluorescein isothiocyanate (#333805, Biolegend).

According to the instructions of the CellTrace carboxyfluorescein diacetate succinimidyl ester (CFSE) Cell Proliferation Kit (C34554, Thermo Fisher Scientific), the CD8^+^ T cells were incubated with CFSE solution (1 mM) at 37 °C for 15 min for labeling, followed by incubation with EC cells. After that, the fluorescence intensity of CFSE was analyzed by flow cytometry to evaluate the proliferation of the CD8^+^ T cells. The gating strategies are shown in Supplementary Material 1.

### Enzyme-linked immunosorbent assay (ELISA)

The concentration of IFN-γ released by CD8^+^ T cells was detected using the human IFN-γ ELISA kit (ab46025, Abcam). Standards of known IFN-γ concentrations, control solution, and the supernatant of T cell and cancer cell cultures, were pipetted into the wells of the microtiter strips pre-coated with IFN-γ-specific monoclonal antibody, 100 μL for each well. After that, each well was further added with 50 µL of 1× Biotinylated anti-IFN-γ for 2 h of incubation at 22–25 °C. The plates were then washed and incubated with streptavidin-HRP solution for 30 min, followed by the addition of tetramethylbenzidine substrate solution for 20 min of dark incubation at 22–25 °C. The OD value of each well at 450 nm was read by a spectrophotometer, and the secretion of IFN-γ by CD8^+^ T cells was calculated based on the curve of standards.

EC cells were replaced with fresh medium and incubated for 24 h. Cell culture medium was collected, and the supernatant was taken by centrifugation at 1000 × *g* for 10 min at 4 °C for assay. Human TGF-β1 ELISA Kit (mlC30466-1, MLBio, Shanghai, China), Human IL-10 ELISA Kit (ab185986, Abcam), and Prostaglandin E2 (PGE2) ELISA Kit (ab316263, Abcam) were used to evaluate the concentration of immunosuppressive factors released in cell culture medium.

PBS (150 μL) supplemented with protease inhibitors was added to 1 × 10^6^ EC cells, and the cells were broken by repeated freeze-thawing. The extracts were centrifuged at 1500 × *g* for 10 min at 4 °C, and the supernatant was removed. The concentration of Indoleamine 2,3-dioxygenase 1 (IDO) in the cell extracts was detected using the Human IDO ELISA Kit (ab245710, Abcam) according to the manufacturer’s protocol.

### Growth and metastasis of xenograft tumors in vivo

Humanized immune system mice (huHSC-NOG-EXL mice) were procured from Charles River Laboratory Animal Technology Co., Ltd (Beijing, China). The experiments were initiated on week 12 after hematopoietic stem cell (HSC) implantation, when all immune cells had already differentiated. All animal experiments were approved by the Animal Ethics Committee of Shanghai First Maternity and Infant Hospital (approval number: TJBG04823101).

For xenograft tumor growth assays, approximately 1 × 10^6^ stably infected AN3CA cells were injected into the huHSC-NOG-EXL mice subcutaneously. Ten mice were maintained in each group (estimated based on previous experience). The volume of xenograft tumors was examined once a week by the following formula: volume = 1/2 × major axis × short axis^2^. The mice were sacrificed by intraperitoneal injection of excessive nembutal (150 mg/kg) after week 4 (day 28), and the subcutaneous tumors were collected. The tumor tissues of five random mice were harvested to prepare a single-cell suspension using the mouse Tumor Dissociation Kit (130-096-730, Miltenyi Biotec Technology & Trading Co., Ltd, Shanghai, China) for flow cytometric analysis. The tumors of the rest five mice in each group were collected for IHC and RT-qPCR assays. Relevant assays were done by researchers who were unaware of the grouping.

For tumor metastasis analyses, approximately 2 × 10^6^ stably transfected AN3CA cells were injected into the huHSC-NOG-EXL mice through the tail vein. The survival of 10 mice in each group (estimated based on previous experience) was monitored for 45 days. On day 45, the surviving mice were sacrificed by nembutal injection as well, and then the lung tissues were collected. Infiltration of metastatic tumors in the lungs was assessed by a hematoxylin-eosin (HE) staining kit (Solarbio) by investigators who were unaware of the grouping.

Xenograft tumors were also induced in immunocompromised BALB/c nude mice (acquired from Charles River as well). ARNTL-KO or wild-type (WT) AN3CA cells were injected into the nude mice subcutaneously (1 × 10^6^ cells/mouse), *n* = 5 for each injection (estimated based on previous experience). The volume of xenograft tumors was examined once a week, and the mice were sacrificed after week 4 as well. The tumors were collected and weighed.

### Western blot (WB) analysis

Total protein from EC cells was extracted using the radio-immunoprecipitation assay lysis buffer (Beyotime) containing protease inhibitors, and the protein concentration was determined by the BCA Protein Colorimetric Assay Kit (Elabscience Biotechnology Co., Ltd., Wuhan, Hubei, China). Equal amounts of protein sample (20 μg) were fractionated by 10% SDS-PAGE and transferred onto the polyvinylidene fluoride membranes (Millipore, Billerica, MA, USA), which were blocked by 5% non-fat milk and probed with the primary antibodies against ARNTL (1:1000, 14268-1-AP, PTGCN), Bcl-2 (1:4000, A21873, ABclonal, Wuhan, Hubei, China), Bax (1:4000, A19684, ABclonal), Cleaved Caspase-3 (1:1000, 9664, Cell Signaling Technology, Beverly, MA, USA), PDL-1 (1:1000, ab205921, Abcam), IL-10 (1:1000, A12255, ABclonal), IL-6 (1:1000, A22222, ABclonal), TNF-α (1:2000, 26405-1-AP, PTGCN), INO80 (1:1000, 18810-1-AP, PTGCN), DHX15 (1:1000, 12265-1-AP, PTGCN), and β-actin (1:2000, ab8227, Abcam) overnight at 4 °C, followed by incubation with the goat anti-rabbit IgG (H + L) (1:1000, A0208, Beyotime) at 22-25 °C for 1 h. The band signals were examined by the BeyoECL Plus Kit (P0018S, Beyotime). The expression of target proteins relative to the control β-actin was analyzed by Image J. Full and uncropped western blots are provided in Supplementary Material 2.

### Dual-luciferase reporter gene assay

The promoter sequences of INO80 (chr17:64506294-64506932) were obtained from the UCSC Genome Browser (https://genome.ucsc.edu/index.html) and inserted into the pPro-RB-Report vectors (RiboBio Co., Ltd., Guangzhou, Guangdong, China) to construct promoter-reporter gene vectors. The constructed vectors were transfected into the EC cells. After 48 h, the Firefly and Renilla luciferase activities in cells were determined using the Dual-Luciferase Reporter Gene Assay Kit (Yeasen).

### Chromatin immunoprecipitation (ChIP)-qPCR

According to the instruction manual of the SimpleChIP Plus Sonication ChIP Kit (#56383, Cell Signaling Technology), the EC cells were crosslinked and ultrasonicated. Truncated chromatin segments (~200–500 bp) were reacted with antibodies against ARNTL (1:50, 14268-1-AP, PTGCN), H3K56ac (1:50, A22565, ABclonal), H3K27ac (1:50, A22264, Abclonal), and H3K18ac (1:50, A7257, Abclonal) for immunoprecipitation. Normal rabbit IgG was used as the isotype control. The precipitated DNA was eluted and purified for qPCR analysis to determine the enrichment of promoter fragments of INO80 or DHX15.

### 5-ethynyl-2’-deoxyuridine (EdU) labeling assay

The DNA replication activity of EC cells was determined using the Cell-Light EdU Apollo643 In Vitro Kit (RiboBio). In short, approximately 1 × 10^5^ cells were seeded in 96-well plates and incubated with 100 μL of EdU reagent (50 μM) for 2 h. The cells were fixed thereafter, penetrated by 0.5% Triton X-100, and incubated with 100 μL of Apollo staining reagent in the dark for 30 min. The nuclei were stained with the Hoechst 33342 solution in the dark for 30 min. The cells were then observed under fluorescence microscopy, and the EdU-positive cells were counted.

### Glucose uptake and lactate production

The glycolytic activity of EC cells was analyzed by a Glucose Uptake Assay Kit (ab136955, Abcam) and an L-Lactate Assay Kit (ab65331, Abcam) in strict accordance with the manufacturer’s protocols. For glucose uptake detection, the cells were incubated with 2-deoxyglucose (2-DG) at 37 °C for 20 min. After that, the cells were lysed to prepare supernatant, which was added to the wells along with standards. The 2-DG taken up by glucose transporters can be metabolized to 2-DG-6-phosphate (2-DG6P), which can be oxidized to generate NADPH, whose level was then determined by an enzymatic recycling amplification reaction. For the lactate production detection, the culture supernatant of EC cells was incubated along with (+)-Lactate standards and reaction mix at 22–25 °C for 30 min. The OD value at 450 nm was then detected to analyze the lactate release.

### Extracellular acidification rate (ECAR) and oxygen consumption rate (OCR)

ECAR and OCR of live cells were analyzed using the Seahorse XFe96 analyzer (Agilent Technologies, Palo Alto, CA, USA). The ECAR of cells was examined using the Seahorse XF Glycolysis Stress Test Kit (Agilent) containing glucose, oligomycin, and 2-DG, and the OCR was detected using the Seahorse XF Cell Mito Stress Test Kit (Agilent) containing oligomycin, FCCP, and rotenone/antimycin A (Agilent). All procedures were conducted following the manufacturer’s instruction manual.

### Statistical analysis

All experimental data were collected from five independent experiments and presented as means ± SD and independent data points. The validity of the sample size was confirmed by post-hoc analysis with G*Power (v3.1.9.7) software, and the statistical efficacy Power (1-β err prob) was greater than 0.8. Prism 8.0.2 software (GraphPad Software Inc., CA, USA) was used for statistical analysis. The Shapiro-Wilk test was applied to test the normality of the data. The Brown-Forsythe test was used to assess the similarity of variance between comparison groups. Differences between groups were compared by the paired or unpaired *t-test*, or by the one-way or two-way analysis of variance (ANOVA). Sidak’s multiple comparisons test or Tukey’s multiple comparisons test was applied for significance analysis after ANOVA. The Log-rank test was used for survival analysis. The enumeration data and the correlations of multiple categorical variables were analyzed by the Chi-square test. In cases where the data did not follow a normal distribution, the Kruskal–Wallis test was employed for comparative analysis, followed by Dunn’s multiple comparisons test for the post-hoc test. A *p*-value less than 0.05 was statistically significant.

## Results

### ARNTL expression is associated with the alteration in T cell and macrophage infiltration in metastatic EC tumors

**Fig. 1:**
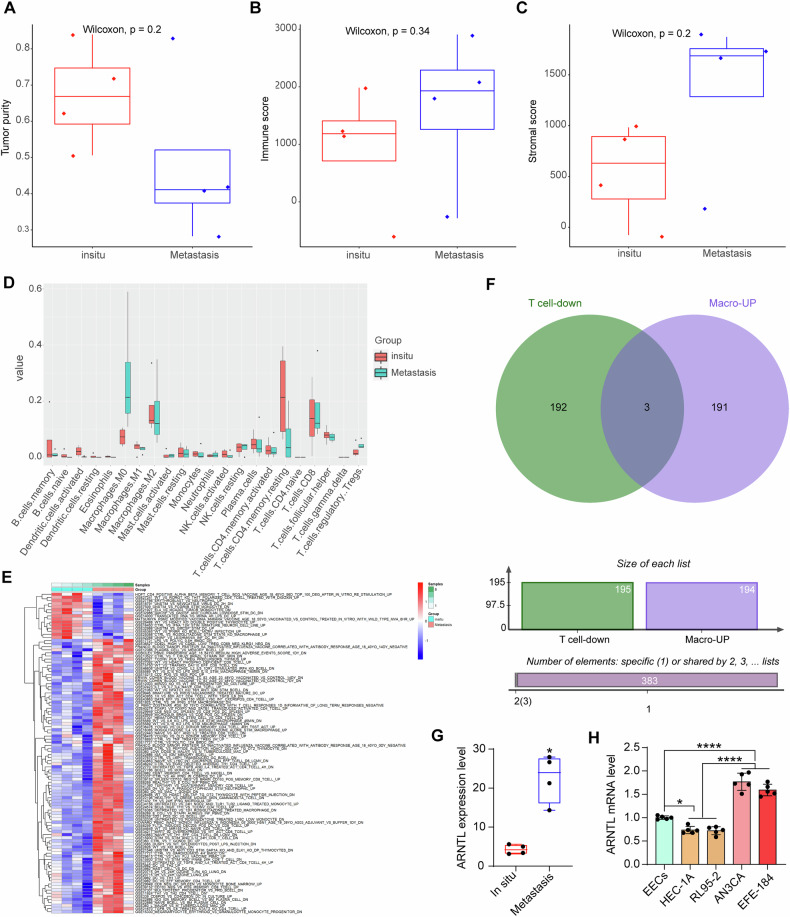
Immune evasion-related gene signatures in metastatic EC samples. Tumor purity (**A**), immune score (**B**) and stromal score (**C**) in in-situ and metastatic EC tumor samples; **D** difference in the infiltrating immune cell types between in-situ and metastatic EC samples; **E** differentially expressed immunologic signature gene sets between in-situ and metastatic EC samples; **F** cross-screening of candidate regulatory genes potentially correlated with differential T cell and macrophage infiltration; **G** ARNTL expression in in-situ and metastatic EC samples analyzed by array analysis (*n* = 4) **H** ARNTL mRNA expression in EECs and EC cell lines (HEC-1A, RL95-2, EFE-184, and AN3CA) determined by RT-qPCR (*n* = 5). All cellular experiments were performed in five biological replicates. Differences were analyzed by the paired *t* test (**G**) or one-way ANOVA (**H**) followed by Tukey’s multiple comparisons test. *p < 0.05, ****p < 0.0001.

The four pairs of in situ and metastatic EC tumor tissue samples were subjected to array analysis. The data were standardized, and the expression data of immune cell-related genes were analyzed to calculate the immune score, stromal score, and ESTIMATE score (tumor purity). Compared to the in-situ tumor tissues, the metastatic tumor tissue samples had reduced tumor purity but elevated immune and stromal scores (Fig. 1A–C), which meet the tumor characteristics.

The fractions of infiltrating immune cells in the eight samples were analyzed by CIBERSORTx. It turned out that macrophages and T cells were the primary immune cell types with substantial alterations between in-situ and metastatic tumor tissues. Overall, macrophages had increased whereas T cells showed decreased infiltration in metastatic tumors (Fig. 1D). The GSVA was thereafter performed to analyze differentially enriched immune cell-related gene sets with *p* < 0.05 as the screening threshold (Fig. 1E). Since the macrophages and T cells are major populations that showed differential abundance between in-situ and metastatic tumor tissues, we primarily focused on the macrophage-related gene sets with upregulation patterns and T-related gene sets with downregulation patterns. The cross-reference revealed three intersections: ARNTL, POSTN, and SPECC1 (Fig. 1F). A disruption of the circadian clock has been correlated with the remodeling of TME and favored tumor cell proliferation [13]. Notably, we observed a significant enrichment of the circadian rhythm regulator ARTIL in metastatic UCEC (referring to uterine corpus EC) (Fig. 1G). Interestingly, RT-qPCR revealed that the expression of ARNTL was decreased in the in-situ EC cell lines (HEC-1A and RL95-2) compared to the normal EECs; however, significantly upregulated ARNTL was detected in the metastatic EC cell lines (AN3CA and EFE-184) (Fig. 1H). This evidence indicates possible correlations between ARNTL expression and the metastatic phenotype of tumor cells and immunosuppression.

### ARNTL is linked to immune cell infiltration and poor prognosis in patients with EC

**Fig. 2:**
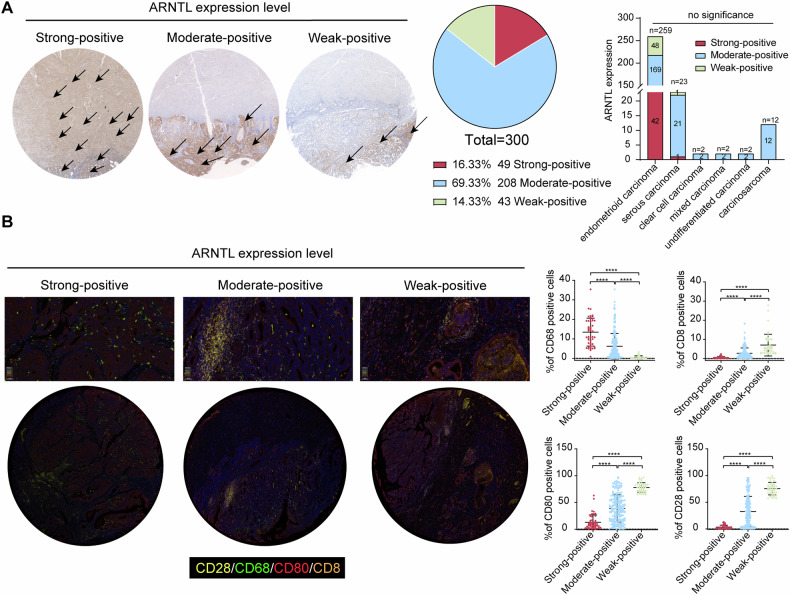
ARNTL is linked to immune cell infiltration and poor prognosis of patients with EC. **A** ARNTL expression in several histological subtypes of tumor tissues examined by IHC assay (black arrows indicate representative positive staining); **B** multi-label immunofluorescence staining of CD28 (yellow), CD68 (green), CD80 (red), and CD8 (orange) in the tumor tissues with different ARNTL levels. Enumeration data were analyzed by the Chi-square test (**A**). Differences between groups were analyzed by the Kruskal–Wallis test (**B**) followed by Dunn’s multiple comparison test. ****p < 0.0001.

Following the insights above, we then analyzed the ARNTL expression in the tumor tissues of 300 patients using IHC. ARNTL expression was detected in all tumor tissues, and patients were allocated into the Strong-positive (*N* = 49), Moderate-positive (*N* = 208), and Weak-positive (*N* = 43) groups. It is noteworthy that the ARNTL expression level showed no significant difference across the various EC histological subtypes (Fig. 2A). After that, the tumor tissue microarrays were subjected to multi-label immunofluorescence staining of CD28 (yellow), CD68 (green), CD80 (red), and CD8 (orange). It was observed that the weak ARNTL expression was linked to reduced TAM marker CD68 but increased T cell markers CD28, CD8, and CD80 (Fig. 2B). The clinical retrospective data of patients showed that the strong positive expression of ARNTL in patients was linked to advanced tumor stage, poor tumor survival, lymph node metastasis, increased CA125 concentration, and increased Ki67 positive rates (Table S2). Again, the ARNTL expression showed no significant differences across the various molecular subtypes (ER status, PR status, and p53 status) (Table S2), indicating a uniformity in the expression/biological functions of ARNTL across these subtypes.

### Knockout of ARNTL suppresses the malignant behavior of EC cells and improves the immune response

**Fig. 3:**
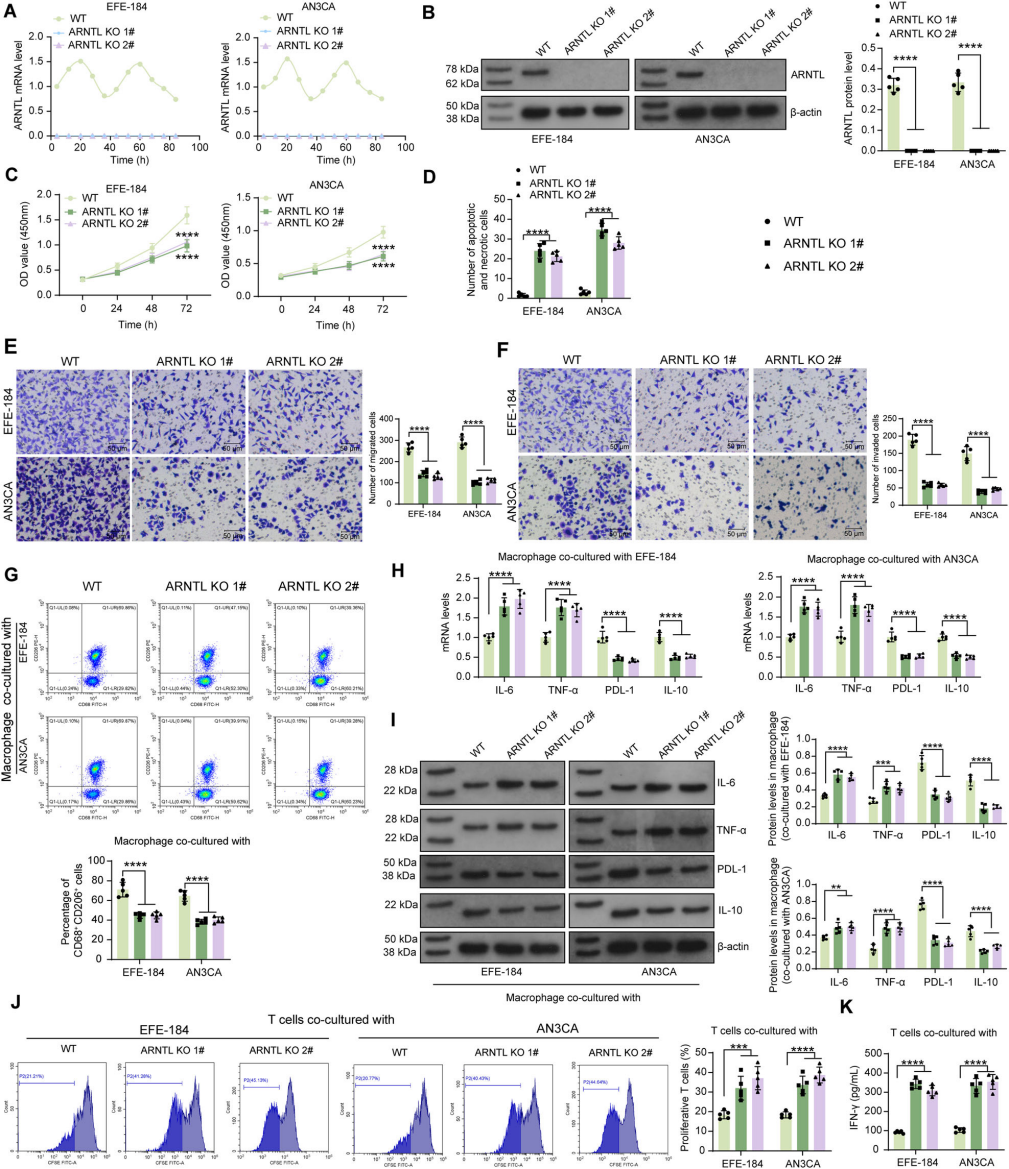
Knockout of ARNTL suppresses the malignant behavior of EC cells and improves immune response. EFE-184 and AN3CA cells were administered two CRISPR/Cas9 lentivirus vectors (ARNTL-KO 1, 2#). mRNA (**A**) and protein (**B**) expression of ARNTL in WT or ARNTL-KO EC cells examined by RT-qPCR and WB analysis, respectively; **C** the proliferation of EC cells analyzed by CCK-8 method; **D** apoptosis and necrosis of EC cells analyzed by YO-PRO-1/PI staining; migration (**E**) and invasion (**F**) of EC cells determined by Transwell assays; **G** the proportion of M2 macrophages after co-culture with WT or ARNTL-KO EC cells analyzed by flow cytometry; mRNA (**H**) and protein (**I**) expression of M1 phenotype markers (IL-6 and TNF-α) and M2 markers (PDL-1 and IL-10) in the co-cultured macrophages analyzed by RT-qPCR and WB analysis, respectively; **J** proliferation of CD8^+^ T cells co-cultured with WT or ARNTL-KO EC cells analyzed by CFSE labeling; **K** concentrations of secretory IFN-γ in the co-culture system of EC cells and CD8^+^ T cells analyzed by ELISA. All cellular experiments were performed in five biological replicates. Differences were analyzed by two-way ANOVA followed by Sidak’s multiple comparisons test. **p < 0.01, ***p < 0.001, ****p < 0.0001.

The two metastatic EC cell lines EFE-184 and AN3CA were used to analyze the functions of ARNTL. Two CRISPR/Cas9 lentivirus vectors targeting ARNTL (Exon 13) (ARNTL-KO 1, 2#) and a specific qPCR primer targeting ARNTL (Exon 13) were designed. RT-qPCR results showed the circadian expression pattern of ARNTL in WT EC cell lines, while no ARNTL expression was detected in cells after ARNTL-KO 1, 2# treatments (Fig. 3A). Similarly, WB analysis showed that the targeting knockout of ARNTL at Exon 13 blocked the formation of functional ARNTL protein (Fig. 3B).

The CCK-8 assay revealed that the ARNTL-KO cells showed significantly reduced proliferation ability (Fig. 3C). The YO-PRO-1/PI staining showed that the ARNTL knockout increased the number of apoptotic and necrotic EC cells (Fig. 3D). WB assay detected that ARNTL knockout attenuated expression of the intracellular apoptosis inhibitor protein Bcl-2 and enhanced expression of the pro-apoptotic proteins Bax and Cleaved Caspase-3 (Fig. S1A). Meanwhile, these cells also showed reduced activity in migration and invasion (Fig. 3E, F).

ARNTL knockout effectively inhibited the release of immunosuppressive factors TGF-β1 and PGE2 from EC cells, and decreased the intracellular level of immunosuppressive factor IDO, but had no significant effect on the secretion of IL-10 (Fig. S1B). The WT or ARNTL-KO EC cells were co-cultured with macrophages and CD8^+^ T cells induced/isolated from PBMCs. The flow cytometry showed that the ARNTL-KO cells reduced the M2 polarization (CD68^+^CD206^+^) of the co-cultured macrophages (Fig. 3G). RT-qPCR and WB analysis showed that the macrophages, after co-cultured with ARNTL-KO EC cells, showed substantially elevated levels of M1 phenotype markers IL-6 and TNF-α, whereas decreased levels of M2 markers PDL-1 and IL-10 (Fig. 3H, I). When it comes to the activity of T cells, the CFSE labeling showed the CD8^+^ T cells had enhanced proliferation when co-cultured with the ARNTL-KO EC cells (Fig. 3J), and the ARNTL-KO condition led to increased concentration of secretory IFN-γ (Fig. 3K). Both vectors (ARNTL-KO 1, 2#) successfully knocked out ARNTL expression in cells and significantly suppressed the malignant phenotype of EC cells. ARNTL KO 1# was applied in the subsequent experiments. AN3CA cells were transplanted into immunocompromised BALB/c nude mice to generate xenograft tumors. Compared to WT cells, the ARNTL-KO cells showed significantly reduced tumorigenic ability, as manifested by decreased tumor volume and weight during the experimental period (Fig. S1C, D).

### Knockout of ARNTL suppresses the growth and metastasis of EC cells and enhances immune response in vivo

**Fig. 4:**
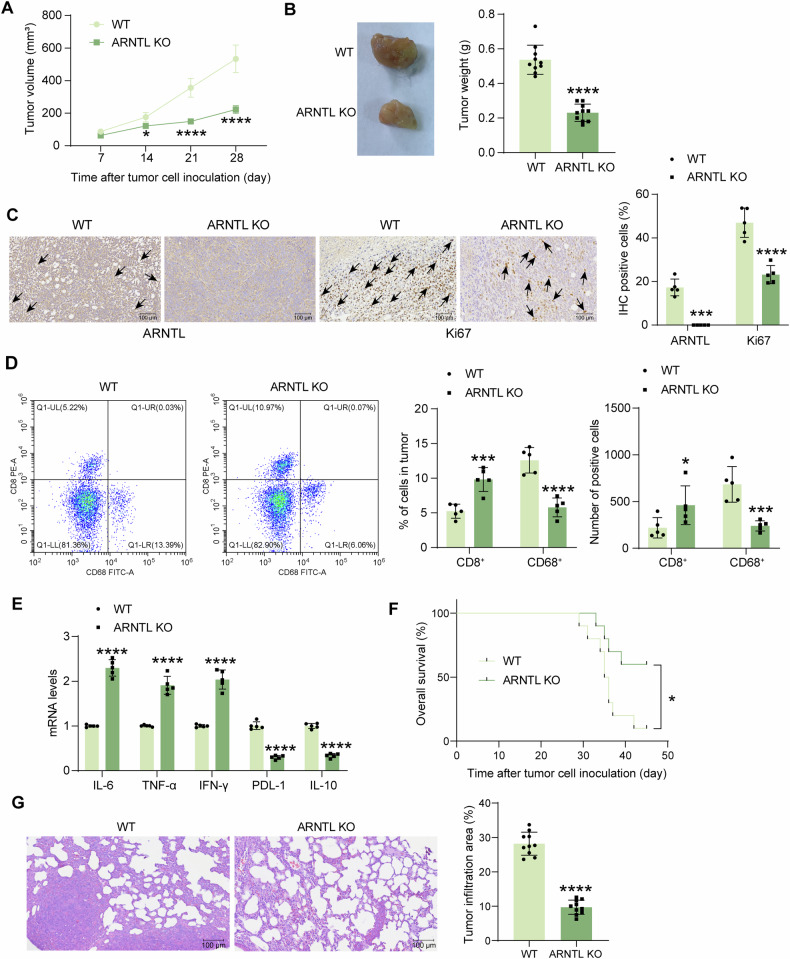
Knockout of ARNTL suppresses the growth and metastasis of EC cells and enhances immune response in vivo. WT AN3CA cells or those stably transfected with ARNTL-KO 1 were injected into immunocompetent huHSC-NOG-EXL mice subcutaneously. **A** Volume of xenograft tumors in mice (*n* = 10); **B** weight of xenograft tumors on day 28 and the representative images (*n* = 10); **C** expression of ARNTL and Ki67 in xenograft tumor tissues analyzed by IHC (black arrows indicate representative positive staining) (*n* = 5); **D** number of infiltrating CD8^+^ T cells and macrophages in xenograft tumors (*n* = 5); **E** mRNA expression of IL-6, TNF-α, IFN-γ, PDL-1, and IL-10 in tumor tissues analyzed by RT-qPCR (*n* = 5). WT AN3CA cells or those administered ARNTL-KO 1 were injected into immunocompetent huHSC-NOG-EXL mice through the tail vein. **F** Survival of mice in two groups within 45 days (*n* = 10); **G** tumor infiltration in mouse lung tissues analyzed by HE staining (*n* = 10). Differences were analyzed by the unpaired *t* test (**B**, **G**) or two-way ANOVA followed by Sidak’s multiple comparisons test (**A**, **C**, **D**, **E**). Survival of mice was analyzed by the Log-rank test (**F**). *p < 0.05, ***p < 0.001, ****p < 0.0001.

Xenograft tumor models were also induced in immunocompetent huHSC-NOG-EXL mice to examine the effect of ARNTL on immune response activity. WT or ARNTL-KO AN3CA cells were injected into mice subcutaneously or through the tail vein for tumor growth and metastasis assessment, respectively. Compared to the WT cells, the ARNTL-KO cells had significantly reduced tumorigenicity in mice, which led to conspicuously decreased tumor volume (Fig. 4A) and tumor weight (week 4) (Fig. 4B). In the xenograft formed by ARNTL-KO cells, ARNTL expression loss was detected, along with decreased immunohistochemical staining of Ki-67, a tumor proliferation marker (Fig. 4C). Flow cytometric analysis of the single-cell suspension of the xenograft tumor tissues showed that the ARNTL knockout increased the number of infiltrating T cells, whereas it decreased the number of infiltrating macrophages (Fig. 4D). Meanwhile, increased expression levels of IL-6, TNF-α, and IFN-γ, whereas decreased levels of PDL-1 and IL-10 were detected in the tumors formed by ARNTL-KO cells (Fig. 4E). In the metastatic models, mice injected with ARNTL-KO cells had a higher survival rate within 45 days compared to those injected with WT cells (Fig. 4F). The lung tissues of tumor-bearing mice were harvested. The HE staining showed that the ARNTL knockout substantially reduced the tumor infiltration in the lung (Fig. 4G).

### ARNTL KO reduces INO80 expression in EC cells

To investigate the downstream molecular mechanisms by which ARNTL knockout exerts its anti-cancer effects, genes significantly positively correlated (top 1000 in terms of the correlation coefficient) with ARNTL expression in UCEC were downloaded from UALCAN (Table S3). Potential downstream target genes of ARNTL were obtained from hTFtarget (https://guolab.wchscu.cn/hTFtarget//#!/) (Table S4). Cross-referencing the two datasets revealed 29 intersecting genes (Fig. S2A). The protein-protein interaction (PPI) network for these genes was constructed using the STRING system (https://string-db.org/). Central proteins in the network were identified using the Maximal Clique Centrality Algorithm in Cytoscape’s CytoHubba plugin (Fig. S2B). Among the top three central factors (PUM2, DDX5, INO80), only INO80 showed a significant change in mRNA expression in ARNTL-KO cells, with a notable decrease (Fig. S2C). The baseline protein level of INO80 in ARNTL-KO cells was found to be decreased as well (Fig. S2D), prompting further investigation. Analysis of ARNTL ChIP-seq data from the Cistrome Data Browser (http://cistrome.org/db/#/) indicated promising binding of ARNTL to the INO80 promoter region (Fig. S2E). Similar to ARNTL, INO80 expression was significantly decreased in primary EC tumor cell lines (HEC-1A and RL95-2), but higher in metastatic EC cell lines (AN3CA and EFE-184) (Fig. S2F). ChIP-qPCR revealed the binding between ARNTL and the INO80 promoter in EC cells (Fig. S2G). Additionally, the dual-luciferase assays showed that ARNTL knockout significantly reduced the transcriptional activity of the INO80 promoter in EC cells (Fig. S2H).

### INO80 overexpression rescues the malignant behavior of the ARNTL-KO EC cells

**Fig. 5:**
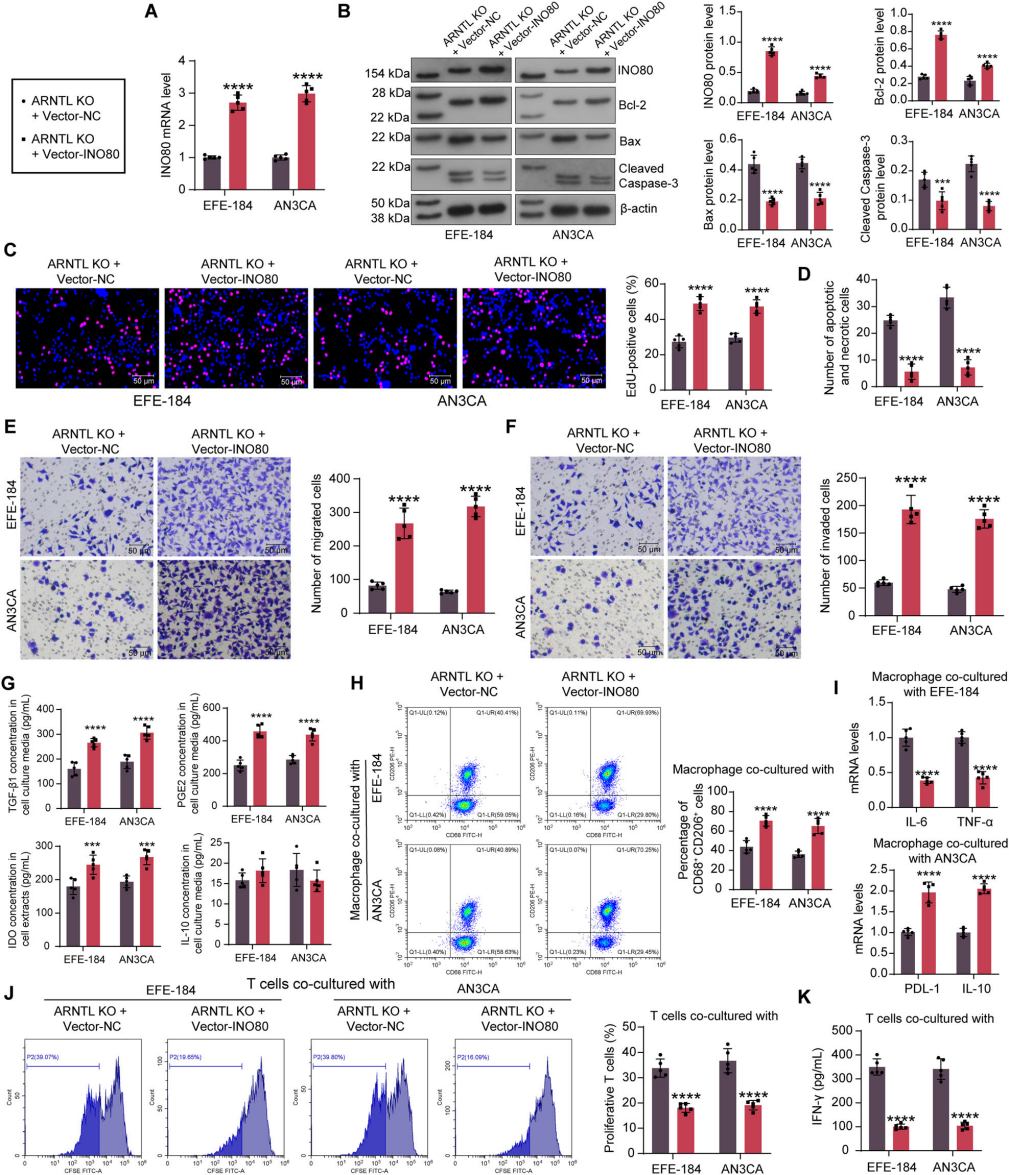
INO80 overexpression rescues the malignant behavior of the ARNTL-KO EC cells. ARNTL-KO EC cells were further administered Vector-INO80 or Vector NC. **A** mRNA expression of INO80 in EC cells determined by RT-qPCR; **B** intracellular INO80, Bcl-2, Bax, Cleaved Caspase-3 protein expression by WB analysis; **C** DNA replication of EC cells analyzed by EdU labeling assay; **D** apoptosis and necrosis of EC cells analyzed by YO-PRO-1/PI staining; migration (**E**) and invasion (**F**) of EC cells determined by Transwell assays; **G** the concentrations of TGF-β1, PGE2, and IL-10 released from EC cells and the intracellular concentration of IDO by ELISA; **H** proportion of M2 macrophages after co-cultured with the treated EC cells analyzed by flow cytometry; **I** expression of M1 phenotype markers (IL-6 and TNF-α) and M2 markers (PDL-1 and IL-10) in the co-cultured macrophages analyzed by RT-qPCR; **J** proliferation of CD8^+^ T cells co-cultured with the EC cells analyzed by CFSE labeling; **K** concentrations of secretory IFN-γ in the co-culture system of EC cells and CD8^+^ T cells analyzed by ELISA. All cellular experiments were performed in five biological replicates. Differences were analyzed by two-way ANOVA followed by Sidak’s multiple comparisons test. ****p < 0.0001.

To investigate whether INO80 participates in the tumor-promoting role of ARNTL in EC, we introduced Vector-ARNTL in the ARNTL-KO EC cells, and the successful exogenous overexpression of INO80 in cells was determined by RT-qPCR (Fig. 5A). WB assay detected that INO80 overexpression also elevated intracellular Bcl-2 protein expression and attenuated Bax and Cleaved Caspase-3 protein expression (Fig. 5B).

After that, the EC cells showed significantly enhanced DNA replication activity (Fig. 5C), reduced apoptosis and necrosis (Fig. 5D), as well as rescued migratory and invasive abilities (Fig. 5E, F).

Overexpression of INO80 also promoted the expression of the immunosuppressive molecule IDO as well as the secretion of TGF-β1 and PGE2 in EC cells and still had no significant effect on IL-10 secretion (Fig. 5G). In the co-culture systems, it was found that the INO80 overexpression in EC cells increased the percentage of M2-polarized (CD68^+^CD206^+^) macrophages according to flow cytometry (Fig. 5H). The RT-qPCR results also suggested that the co-cultured macrophages presented elevated expression of PDL-1 and IL-10 but decreased expression of IL-6 and TNF-α (Fig. 5I). Moreover, the INO80 overexpression in EC cells suppressed the proliferation activity of the co-cultured CD8^+^ T cells (Fig. 5J) and reduced the secretion of IFN-γ (Fig. 5K).

### Forced expression of INO80 accelerates the tumorigenicity and metastasis of EC cells in vivo and weakens the immune response

**Fig. 6:**
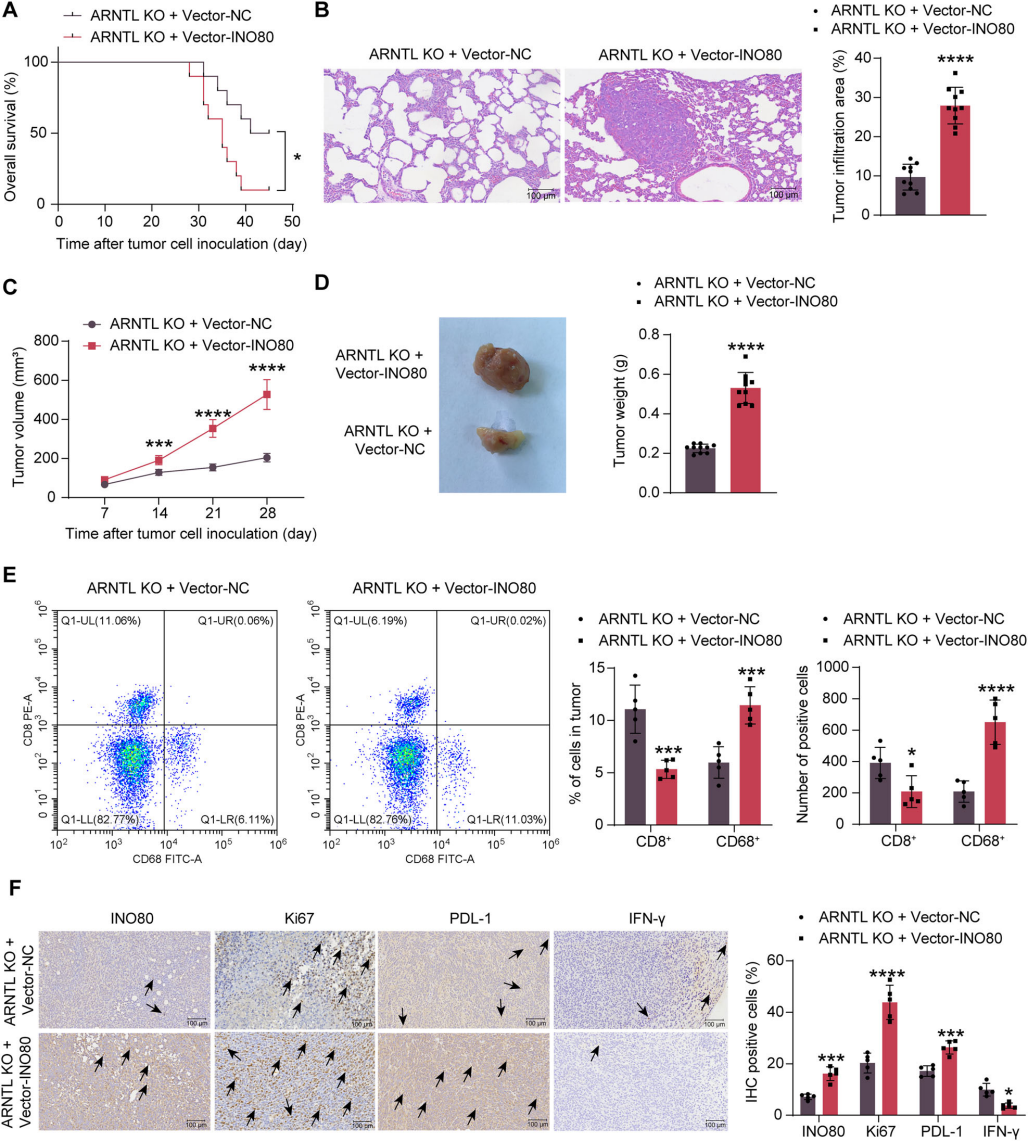
Forced expression of INO80 accelerates tumorigenicity and metastasis of EC cells in vivo and weakens the immune response. AN3CA cells administered ARNTL-KO 1 + Vector-INO80/Vector-NC were injected into immunocompetent huHSC-NOG-EXL mice through the tail vein. **A** Survival of mice within 45 days (*n* = 10); **B** tumor infiltration in mouse lung tissues analyzed by HE staining (*n* = 10). AN3CA cells administered ARNTL-KO 1 + Vector-INO80/Vector-NC were injected into immunocompetent huHSC-NOG-EXL mice subcutaneously. **C** volume of xenograft tumors in mice (*n* = 10); **D** weight of xenograft tumors on day 28 and the representative images (*n* = 10); **E** number of infiltrating T cells and macrophages in xenograft tumors (*n* = 5); **F** expression of INO80, Ki-67, PDL-1, and IFN-γ in tumor tissues analyzed by IHC (black arrows indicate representative positive staining) (*n* = 5). Differences were analyzed by the unpaired *t* test (**B**, **D**) or two-way ANOVA followed by Sidak’s multiple comparisons test (**C**, **E**, **F**). Survival of mice was analyzed by the Log-rank test (**A**). *p < 0.05, ***p < 0.001, ****p < 0.0001.

After INO80 was artificially upregulated in the ARNTL-KO AN3CA cells, tail vein injection of these cells led to an increased mortality rate of the huHSC-NOG-EXL mice (Fig. 6A) and increased tumor metastasis burden in their lung tissues (Fig. 6B). In the setting of subcutaneous injection, the INO80-overexpressing cells also increased the growth rate and weight of the xenograft tumors (Fig. 6C, D), along with reduced T cell infiltration but increased macrophage infiltration within (Fig. 6E). Moreover, the IHC assay showed that the INO80 upregulation increased Ki67 and PDL-1 levels but a decline in IFN-γ expression in the subcutaneous xenograft tumors (Fig. 6F).

### The ARNTL-INO80 axis mediates DHX15 expression

INO80 is a chromatin remodeler that modulates histone acetylation and accessibility of target gene promoters to modulate glycolysis and respiration [27]. To further investigate whether the ARNTL-INO80 axis modulates EC progression by modulating glycolysis, genes significantly correlated with INO80 expression in UCEC were downloaded from UALCAN (Table S5), and the top 1000 predicted target genes of INO80 were obtained from hTFtarget (Table S6). A list of glycolysis-related genes was downloaded from the GeneCards system (https://www.genecards.org/) using the keyword “Glycolysis” (Table S7). The cross-reference analysis revealed 10 intersecting genes (Fig. S3A). The PPI network for these intersecting genes identified four central proteins with protein node degrees ≥ 3: APC, DHX15, PRKAR1A, and SP3 (Fig. S3B). Among them, DHX15 was selected for further analysis since it presented the highest degree of alteration upon INO80 overexpression in EC cells (Fig. S3C). Interestingly, RT-qPCR experiments revealed that DHX15 expression was elevated in metastatic but not primary EC cell lines compared to EECs (Fig. S3D). In metastatic EC cells, the DHX15 expression was reduced upon ARNTL knockout but significantly restored after INO80 overexpression (Fig. S3E, F). ChIP-seq data from the Cistrome Data Browser showed significant histone acetylation modifications near the DHX15 promoter region (Fig. S3G). Complementing this evidence, ChIP-qPCR analysis revealed that ARNTL knockout led to decreased histone acetylation (H3K56ac, H3K27ac, H3K18ac) levels near the DHX15 promoter region, which were rescued by forced overexpression of INO80 in ECs (Fig. S3H).

### Overexpression of INO80 or DHX15 restores glycolytic reprogramming in ARNTL-KO EC cells

**Fig. 7:**
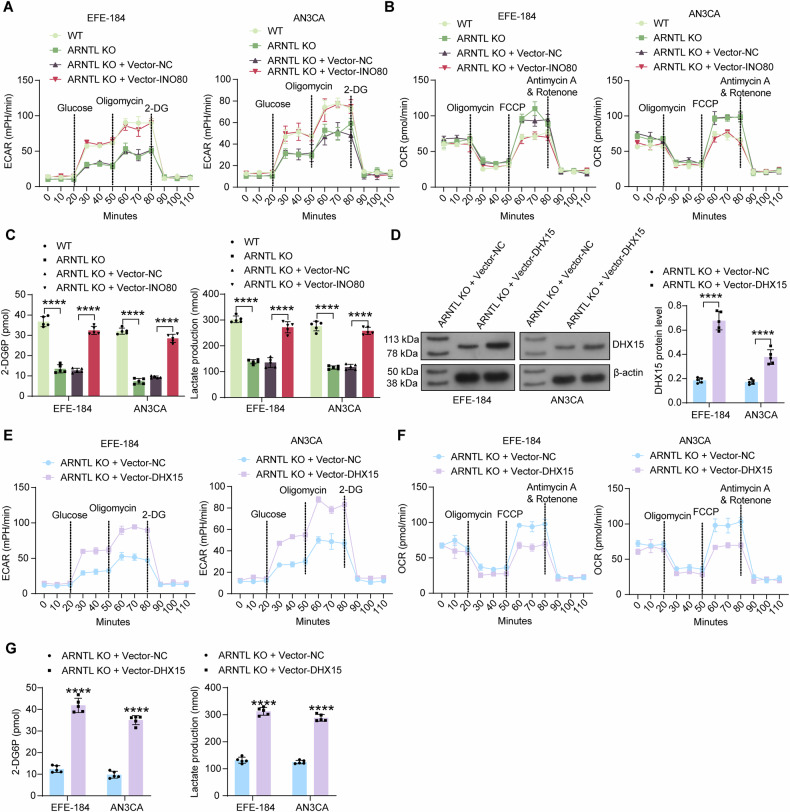
Overexpression of INO80 or DHX15 restores glycolytic reprogramming in ARNTL-KO EC cells. **A** Glycolytic activity in EFE-184 and AN3CA cells with ARNTL KO or INO80 overexpression evaluated by ECAR; **B** mitochondrial respiration in EC cells evaluated by OCR; **C** glucose uptake and lactate production in EC cells determined by colorimetry. ARNTL-KO EC cells were further administered Vector-DHX15 or Vector NC. **D** protein expression of DHX15 in cells determined by WB analysis; **E** glycolytic activity in cells evaluated by ECAR; **F** mitochondrial respiration in cells evaluated by OCR; **G** glucose uptake and lactate production in cells determined by colorimetry. All cellular experiments were performed in five biological replicates. Differences were analyzed by two-way ANOVA followed by Sidak’s multiple comparisons test (**D**, **G**) or Tukey’s multiple comparisons test (**C**). ****p < 0.0001.

Regarding the roles of ARNTL, INO80, and DHX15 in glycolytic activity in EC cells, ARNTL-KO EC cells showed decreased ECAR but increased OCR (Fig. 7A, B), as well as decreased glucose uptake and lactate production (Fig. 7C). Forced expression of INO80 in these cells restored ECAR and decreased OCR, and it rescued glucose uptake and lactate production (Fig. 7A–C). Additionally, we induced DHX15 overexpression in ARNTL-KO EC cells using lentivirus vectors. The successful DHX15 protein upregulation was confirmed by WB analysis (Fig. 7D). Notably, the DHX15 overexpression significantly restored ECAR while decreasing OCR in the ARNTL-KO EC cells (Fig. 7E, F). Additionally, the glucose uptake and lactate production in cells were increased upon the DHX15 overexpression as well (Fig. 7G).

Additionally, to analyze the interaction between INO80 and DHX15, as well as their roles in glycolysis in EC, AN3CA, and EFE-184 cells were administered lentivirus vectors-encapsulated sh-INO80 and Vector-DHX15 were administered. The sh-INO80 transfection reduced INO80 expression and then decreased DHX15 levels, and the additional Vector-DHX15 transfection restored DHX15 expression without altering INO80 expression (Fig. S4A). The INO80 knockdown in cells significantly reduced ECAR while increasing OCR (Fig. S4B, C), accompanied by a decrease in glucose uptake and lactate production (Fig. S4D). Again, these changes were negated by the additional DHX15 overexpression in cells (Fig. S4B–D).

### DHX15 regulates immune response and tumor progression in EC

**Fig. 8:**
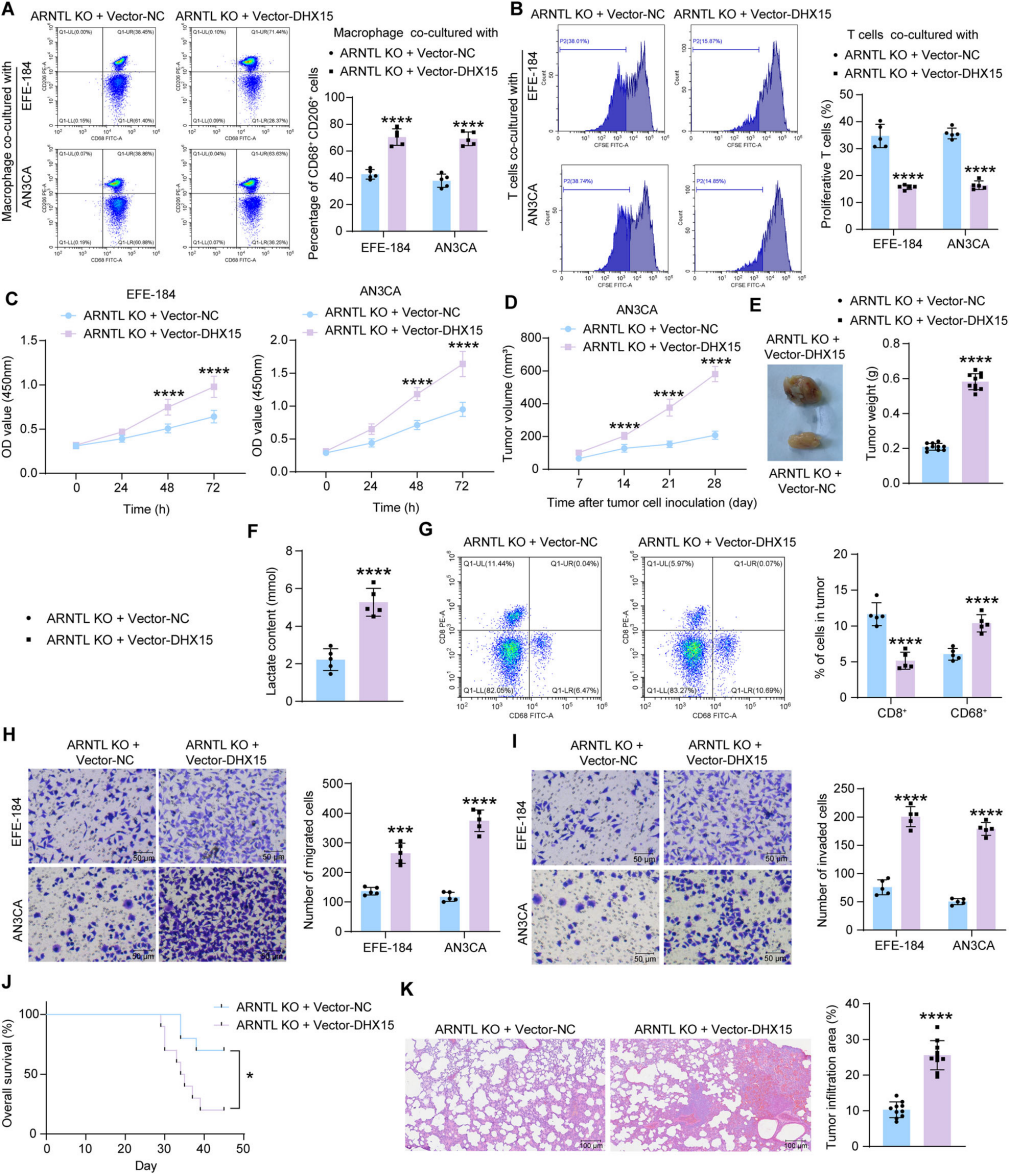
DHX15 regulates immune response and tumor progression in EC. Vector DHX15/Vector NC was administered into the ARNTL-KO EC cells. **A** The proportion of M2 macrophages after co-cultured with the EC cells analyzed by flow cytometry (*n* = 3); **B** proliferation of CD8^+^ T cells co-cultured with the EC cells analyzed by CFSE labeling (*n* = 3); **C** proliferation of the EC cells analyzed by CCK-8 method (*n* = 3). AN3CA cells administered ARNTL-KO 1 + Vector-INO80/Vector NC were injected into immunocompetent huHSC-NOG-EXL mice subcutaneously. **D** volume of xenograft tumors in mice (*n* = 10); **E** weight of xenograft tumors on day 28 and the representative images (*n* = 10); **F** lactate content in xenograft tumors analyzed by colorimetry (*n* = 5); **G** number of infiltrating CD8^+^ T cells and macrophages in xenograft tumors determined by flow cytometry (*n* = 5); migration (**H**) and invasion (**I**) of EC cells determined by Transwell assays (*n* = 3). AN3CA cells administered ARNTL-KO 1 + Vector-INO80/Vector NC were injected into immunocompetent huHSC-NOG-EXL mice via the tail vein. **J** survival of mice within 45 days after tail vein injection of EC cells (*n* = 10); **K** tumor infiltration in mouse lung tissues analyzed by HE staining (*n* = 10). Differences were analyzed by the unpaired *t* test (**E**, **F**, **K**) or two-way ANOVA followed by Sidak’s multiple comparisons tests (**A**–**D**, **G**–**I**). All cellular experiments were performed in three biological replicates. Survival of mice was analyzed by the Log-rank test (**J**). ***p < 0.001, ****p < 0.0001.

Overexpression of DHX15 in ARNTL knockout cells effectively promoted the release of immunosuppressive factors TGF-β1 and PGE2, and enhanced intracellular IDO expression, but had no significant effect on IL-10 release, as detected by ELISA (Fig. S5A). To analyze the interaction of glycolytic reprogramming with immune response activity in EC cells, we analyzed the macrophage phenotype and T cell activity when the immune cells were co-cultured with ARNTL-KO EC cells overexpressing DHX15. Importantly, the DHX15 overexpression in EC cells conspicuously increased the M2 polarization of the co-cultured M2 macrophages (Fig. 8A) and inhibited the proliferation of the co-cultured CD8^+^ T cells (Fig. 8B).

WB detected elevated Bcl-2 expression in EC cells overexpressing DHX15, along with attenuated expression of Bax and Cleaved Caspase-3 (Fig. S5B). Meanwhile, the DHX15 overexpression also promoted the proliferation of EC cells in vitro (Fig. 8C) and increased the tumorigenicity of cells in mice (Fig. 8D, E). The DHX15 overexpression in EC cells also increased the lactate content in the subcutaneous tumors (Fig. 8F), along with increased macrophage infiltration, whereas decreased CD8^+^ T cell infiltration (Fig. 8G). Moreover, the Vector-DHX15 augmented the migration and invasion of cells in vitro (Fig. 8H, I), and it increased the mortality rate of mice and promoted lung metastasis in vivo when the cells were injected through the tail vein (Fig. 8J, K).

## Discussion

To date, the driver molecules involved in tumor immune evasion in EC remain largely unknown. In this study, we report that the circadian regulator ARNTL participates in the formation of immunosuppressive TME, glycolytic reprogramming, and metastasis in EC by regulating the INO80-DHX15 axis.

Here, we found that the metastatic tumor samples had reduced tumor purity with increased immune cell infiltration compared to the primary tumors, with a major increase in the population of macrophages and a decrease in the population of T cells. Partly consistent with our findings, Yin and colleagues found a similar composition of tumor-infiltrating immune cells in high- and low-risk EC sample groups, while the high-risk samples showed a significantly increased population of M2 macrophages with a relatively low population of T cells [28]. Our subsequent integrated bioinformatics analyses based on the gene expression profiles identified ARNTL as a promising key regulator linked to the aberrant immune cell infiltration in metastatic EC samples. ARNTL plays either tumor-suppressive [19, 29] or oncogenic [30] roles in human malignancies depending on the specific cellular or tissue contexts and the distinct downstream cascades. The correlation between ARNTL and immune response in tumors has been well established in glioblastoma, where ARNTL has been found to help recruit immune-suppressive microglia to the TME while reducing CD8^+^ T cell infiltration [16, 31]. Here, the higher expression of ARNTL was linked to worse clinical presentations of patients with EC. Notably, heightened ARNTL expression was only detected in metastatic EC cell lines but not in primary EC cell lines. This interesting observation highlights the potential correlation between ARNTL upregulation and EC cell metastasis. Furthermore, we found that KO of ARNTL in metastatic EC cell lines suppressed their malignant properties both in vitro and in vivo, which also suppressed the M2 skewing of the macrophages and increased the proliferation and activity of CD8^+^ T cells in TME. Consistently, ARNTL knockdown in glioblastoma cell lines induced an M2-to-M1 skewing of macrophages, thus reducing immunosuppression and favoring tumor elimination [32].

Our subsequent cross-screening bioinformatics analyses suggested INO80 as a target of ARNTL that is correlated with differential macrophage and CD8^+^ T cell infiltration in EC. Interestingly, we identified specific INO80 upregulation in metastatic EC cells, which was reduced upon ARNTL KO, indicating it as a direct target of ARNTL in this context. In non-small cell lung cancer, INO80 has been demonstrated to play a critical role in promoting oncogenic transcription and tumorigenesis [33]. In terms of anti-tumor immunity, INO80 has been found to cause T cell exhaustion, with its perturbation improving T cell persistence in tumors [34]. Considering the correlation between glycolysis and immunosuppression, there might be specific links between the glycolysis-regulatory role of INO80 to the immunosuppressive events. Notably, we found that restoring INO80 expression in ARNTL-KO cells rescued the glycolytic activity of cells and promoted M2 polarization of the co-cultured macrophages while suppressing CD8^+^ T cell activity. These cells also presented rescued tumorigenic activity in mice while reducing immune activity in xenograft tumors. This evidence demonstrates the implication of INO80 in ARNTL-mediated oncogenic and immunosuppressive events.

DHX15, which was identified as a downstream target of INO80, was found to present a consistent expression pattern like ARNTL and INO80 in the EC cell lines, and its expression was found to be reduced upon ARNTL KO and upregulated upon INO80 overexpression. Specifically, ARNTL KO led to reduced levels of H3K56ac, H3K27ac, and H3K18ac near the DHX15 promoter, which might be ascribed to reduced INO80 levels. DHX15 has been reported to form a positive feedback loop with the oncogenic transcription factor NF-κB p65, thus promoting breast cancer progression [35]. Increased DHX15 expression has also been correlated with a poor prognosis in hepatocellular carcinoma patients [36]. Existing evidence has demonstrated that DHX15 knockdown in mosquito cells downregulates genes involved in the glycolytic process and suppresses lactate production [22]. Moreover, loss of DHX15 has been found to inhibit energy metabolism in endothelial cells, and DHX15^+/-^ mice presented partially inhibited tumor growth and reduced lung metastasis after injection of tumor cells [37]. Notably, we found that DHX15 restoration in ARNTL-KO cells similarly rescued the glycolytic and tumorigenic activities of cells, as well as promoted an immunosuppressive phenotype both in vitro and in vivo. ARNTL silencing has been demonstrated to suppress lactate production (glycolysis) in glioblastoma [32]. The findings of this study may provide novel evidence that the INO80-DHX15 axis is possibly implicated in ARNTL’s effects on glycolysis stimulation and immunosuppression during tumor progression.

## Conclusions

In conclusion, by conducting abundant bioinformatics analyses and functional trials, we demonstrate that the circadian rhythm regulator ARNTL directs metastasis, glycolytic reprogramming, and immunosuppression in EC by transcriptionally activating the INO80-DHX15 axis. However, several limitations should be acknowledged. A correlation analysis between ARNTL and INO80 expression was not performed in the cohort of 300 patient samples due to the consumption of samples during the earlier experiments. Additionally, all cellular experiments were not performed on patient-derived EC cells due to ethical, time, and budget constraints. Nevertheless, the commercially purchased EC cell lines were authenticated and suitable as alternatives. The current evidence demonstrates that the ARNTL-INO80-DHX15 axis may represent a critical oncogenic cascade in EC or other malignancies. Although more related basic research is warranted to validate feasibility, using specific inhibitory agents of any members of this axis may be a promising strategy for EC treatment, especially when used in combination with anti-tumor immune therapies.

## Supplementary information

Supplementary Figure S1-5

Table S1: Primer sequences for RT-qPCR

Table S2: Correlation between ARNTL expression and the clinical parameters of patients with EC (N = 300)

Table S3: ARNTL-correlated genes.

Table S4: Target genes of ARNTL.

Table S5. INO80-correlated genes.

Table S6. Target genes of INO80.

Table S7. Glycolysis-correlated genes.

The gating strategies

Original western blots

## Data Availability

The dataset generated and/or analyzed during the current study is available from the corresponding author upon reasonable request.

## References

[1] Makker V, MacKay H, Ray-Coquard I, Levine DA, Westin SN, Aoki D, et al. Endometrial cancer. Nat Rev Dis Prim. 2021;7:88. 34887451 10.1038/s41572-021-00324-8 PMC9421940

[2] Bray F, Laversanne M, Sung H, Ferlay J, Siegel RL, Soerjomataram I, et al. Global cancer statistics 2022: GLOBOCAN estimates of incidence and mortality worldwide for 36 cancers in 185 countries. CA Cancer J Clin. 2024;74:229–63. 38572751 10.3322/caac.21834

[3] Sung H, Ferlay J, Siegel RL, Laversanne M, Soerjomataram I, Jemal A, et al. Global cancer statistics 2020: GLOBOCAN estimates of incidence and mortality worldwide for 36 cancers in 185 countries. CA Cancer J Clin. 2021;71:209–49. 33538338 10.3322/caac.21660

[4] Tashireva LA, Larionova IV, Ermak NA, Maltseva AA, Livanos EI, Kalinchuk AY, et al. Predicting immunotherapy efficacy in endometrial cancer: focus on the tumor microenvironment. Front Immunol. 2024;15:1523518. 39902047 10.3389/fimmu.2024.1523518 PMC11788352

[5] Choi Y, Shi Y, Haymaker CL, Naing A, Ciliberto G, Hajjar J. T-cell agonists in cancer immunotherapy. J Immunother Cancer. 2020;8. 10.1136/jitc-2020-000966 PMC7537335 33020242

[6] van der Leun AM, Thommen DS, Schumacher TN. CD8(+) T cell states in human cancer: insights from single-cell analysis. Nat Rev Cancer. 2020;20:218–32. 32024970 10.1038/s41568-019-0235-4 PMC7115982

[7] Thommen DS, Schumacher TN. T cell dysfunction in cancer. Cancer Cell. 2018;33:547–62. 29634943 10.1016/j.ccell.2018.03.012 PMC7116508

[8] Xiang X, Wang J, Lu D, Xu X. Targeting tumor-associated macrophages to synergize tumor immunotherapy. Signal Transduct Target Ther. 2021;6:75. 33619259 10.1038/s41392-021-00484-9 PMC7900181

[9] Cassetta L, Pollard JW. Tumor-associated macrophages. Curr Biol. 2020;30:R246–R248. 32208142 10.1016/j.cub.2020.01.031

[10] Shen L, Zhou Y, He H, Chen W, Lenahan C, Li X, et al. Crosstalk between macrophages, T cells, and iron metabolism in tumor microenvironment. Oxid Med Cell Longev. 2021;2021:8865791. 33628389 10.1155/2021/8865791 PMC7889336

[11] Jiang Y, Li S, Xu W, Ying J, Qu Y, Jiang X, et al. Critical roles of the circadian transcription factor BMAL1 in reproductive endocrinology and fertility. Front Endocrinol. 2022;13:818272. 10.3389/fendo.2022.818272 PMC8924658 35311235

[12] Papagiannakopoulos T, Bauer MR, Davidson SM, Heimann M, Subbaraj L, Bhutkar A, et al. Circadian Rhythm Disruption Promotes Lung Tumorigenesis. Cell Metab. 2016;24:324–31. 27476975 10.1016/j.cmet.2016.07.001 PMC5367626

[13] Aiello I, Fedele MLM, Roman F, Marpegan L, Caldart C, Chiesa JJ, et al. Circadian disruption promotes tumor-immune microenvironment remodeling favoring tumor cell proliferation. Sci Adv. 2020;6. 10.1126/sciadv.aaz4530 PMC7556830 33055171

[14] Shih MC, Yeh KT, Tang KP, Chen JC, Chang JG. Promoter methylation in circadian genes of endometrial cancers detected by methylation-specific PCR. Mol Carcinog. 2006;45:732–40. 16683245 10.1002/mc.20198

[15] Yin Z, Li F, Zhou Q, Zhu J, Liu Z, Huang J, et al. A ferroptosis-related gene signature and immune infiltration patterns predict the overall survival in acute myeloid leukemia patients. Front Mol Biosci. 2022;9:959738. 36046602 10.3389/fmolb.2022.959738 PMC9421034

[16] Chen P, Hsu WH, Chang A, Tan Z, Lan Z, Zhou A, et al. Circadian regulator CLOCK recruits immune-suppressive microglia into the GBM tumor microenvironment. Cancer Discov. 2020;10:371–81. 31919052 10.1158/2159-8290.CD-19-0400 PMC7058515

[17] Willhoft O, Wigley DB. INO80 and SWR1 complexes: the non-identical twins of chromatin remodelling. Curr Opin Struct Biol. 2020;61:50–58. 31838293 10.1016/j.sbi.2019.09.002 PMC7171469

[18] Morrison AJ. Chromatin-remodeling links metabolic signaling to gene expression. Mol Metab. 2020;38:100973. 32251664 10.1016/j.molmet.2020.100973 PMC7300377

[19] Chen K, Li H, Li Y, Yang Z, Luo J, Zhou Z. ARNTL inhibits the malignant behaviors of oral cancer by regulating autophagy in an AKT/mTOR pathway-dependent manner. Cancer Sci. 2023;114:3914–23. 37562810 10.1111/cas.15928 PMC10551587

[20] Reinfeld BI, Rathmell WK, Kim TK, Rathmell JC. The therapeutic implications of immunosuppressive tumor aerobic glycolysis. Cell Mol Immunol. 2022;19:46–58. 34239083 10.1038/s41423-021-00727-3 PMC8752729

[21] Asselin-Mullen P, Chauvin A, Dubois ML, Drissi R, Levesque D, Boisvert FM. Protein interaction network of alternatively spliced NudCD1 isoforms. Sci Rep. 2017;7:12987. 29021621 10.1038/s41598-017-13441-w PMC5636827

[22] Rosendo Machado S, Qu J, Koopman WJH, Miesen P. The DEAD-box RNA helicase Dhx15 controls glycolysis and arbovirus replication in Aedes aegypti mosquito cells. PLoS Pathog. 2022;18:e1010694. 36441781 10.1371/journal.ppat.1010694 PMC9731432

[23] Yoshihara K, Shahmoradgoli M, Martinez E, Vegesna R, Kim H, Torres-Garcia W, et al. Inferring tumour purity and stromal and immune cell admixture from expression data. Nat Commun. 2013;4:2612. 24113773 10.1038/ncomms3612 PMC3826632

[24] Zeng D, Ye Z, Shen R, Yu G, Wu J, Xiong Y, et al. IOBR: Multi-omics immuno-oncology biological research to decode tumor microenvironment and signatures. Front Immunol. 2021;12:687975. 34276676 10.3389/fimmu.2021.687975 PMC8283787

[25] Newman AM, Liu CL, Green MR, Gentles AJ, Feng W, Xu Y, et al. Robust enumeration of cell subsets from tissue expression profiles. Nat Methods. 2015;12:453–7. 25822800 10.1038/nmeth.3337 PMC4739640

[26] Hanzelmann S, Castelo R, Guinney J. GSVA: gene set variation analysis for microarray and RNA-seq data. BMC Bioinform. 2013;14:7. 10.1186/1471-2105-14-7 PMC3618321 23323831

[27] Beckwith SL, Schwartz EK, Garcia-Nieto PE, King DA, Gowans GJ, Wong KM, et al. The INO80 chromatin remodeler sustains metabolic stability by promoting TOR signaling and regulating histone acetylation. PLoS Genet. 2018;14:e1007216. 29462149 10.1371/journal.pgen.1007216 PMC5834206

[28] Weijiao Y, Fuchun L, Mengjie C, Xiaoqing Q, Hao L, Yuan L, et al. Immune infiltration and a ferroptosis-associated gene signature for predicting the prognosis of patients with endometrial cancer. Aging. 2021;13:16713–32. 34170849 10.18632/aging.203190 PMC8266342

[29] Zhao D, Dong Y, Duan M, He D, Xie Q, Peng W, et al. Circadian gene ARNTL initiates circGUCY1A2 transcription to suppress non-small cell lung cancer progression via miR-200c-3p/PTEN signaling. J Exp Clin Cancer Res. 2023;42:229. 37667322 10.1186/s13046-023-02791-1 PMC10478228

[30] Qu M, Zhang G, Qu H, Vu A, Wu R, Tsukamoto H, et al. Circadian regulator BMAL1::CLOCK promotes cell proliferation in hepatocellular carcinoma by controlling apoptosis and cell cycle. Proc Natl Acad Sci USA. 2023;120:e2214829120. 36595671 10.1073/pnas.2214829120 PMC9926257

[31] Xuan W, Hsu WH, Khan F, Dunterman M, Pang L, Wainwright DA, et al. Circadian Regulator CLOCK Drives Immunosuppression in Glioblastoma. Cancer Immunol Res. 2022;10:770–84. 35413115 10.1158/2326-6066.CIR-21-0559 PMC9177794

[32] Wang F, Liao W, Li C, Zhu L. Silencing BMAL1 promotes M1/M2 polarization through the LDHA/lactate axis to promote GBM sensitivity to bevacizumab. Int Immunopharmacol. 2024;134:112187. 38733825 10.1016/j.intimp.2024.112187

[33] Zhang S, Zhou B, Wang L, Li P, Bennett BD, Snyder R, et al. INO80 is required for oncogenic transcription and tumor growth in non-small cell lung cancer. Oncogene. 2017;36:1430–9. 27641337 10.1038/onc.2016.311 PMC6534818

[34] Belk JA, Yao W, Ly N, Freitas KA, Chen YT, Shi Q, et al. Genome-wide CRISPR screens of T cell exhaustion identify chromatin remodeling factors that limit T cell persistence. Cancer Cell. 2022;40:768–786.e767. 35750052 10.1016/j.ccell.2022.06.001 PMC9949532

[35] Zheng W, Wang X, Yu Y, Ji C, Fang L. CircRNF10-DHX15 interaction suppressed breast cancer progression by antagonizing DHX15-NF-kappaB p65 positive feedback loop. Cell Mol Biol Lett. 2023;28:34. 37101128 10.1186/s11658-023-00448-7 PMC10131429

[36] Xie C, Liao H, Zhang C, Zhang S. Overexpression and clinical relevance of the RNA helicase DHX15 in hepatocellular carcinoma. Hum Pathol. 2019;84:213–20. 30339968 10.1016/j.humpath.2018.10.006

[37] Ribera J, Portoles I, Cordoba-Jover B, Rodriguez-Vita J, Casals G, Gonzalez-de la Presa B, et al. The loss of DHX15 impairs endothelial energy metabolism, lymphatic drainage and tumor metastasis in mice. Commun Biol. 2021;4:1192. 34654883 10.1038/s42003-021-02722-w PMC8519955

