# Supplementary material for: ARNTL-mediated INO80-DHX15 axis reprograms the glycolytic metabolism and augments the progression of endometrial carcinoma
Source: Cell Death Dis. 2025 Jun 20;16(1):463. doi: 10.1038/s41419-025-07776-w (PMC12181345; doi:10.1038/s41419-025-07776-w)
Supplement: Supplementary file 1 — Supplementary Figure S1-5 [file 41419_2025_7776_MOESM1_ESM.docx]

**Fig S1**

**
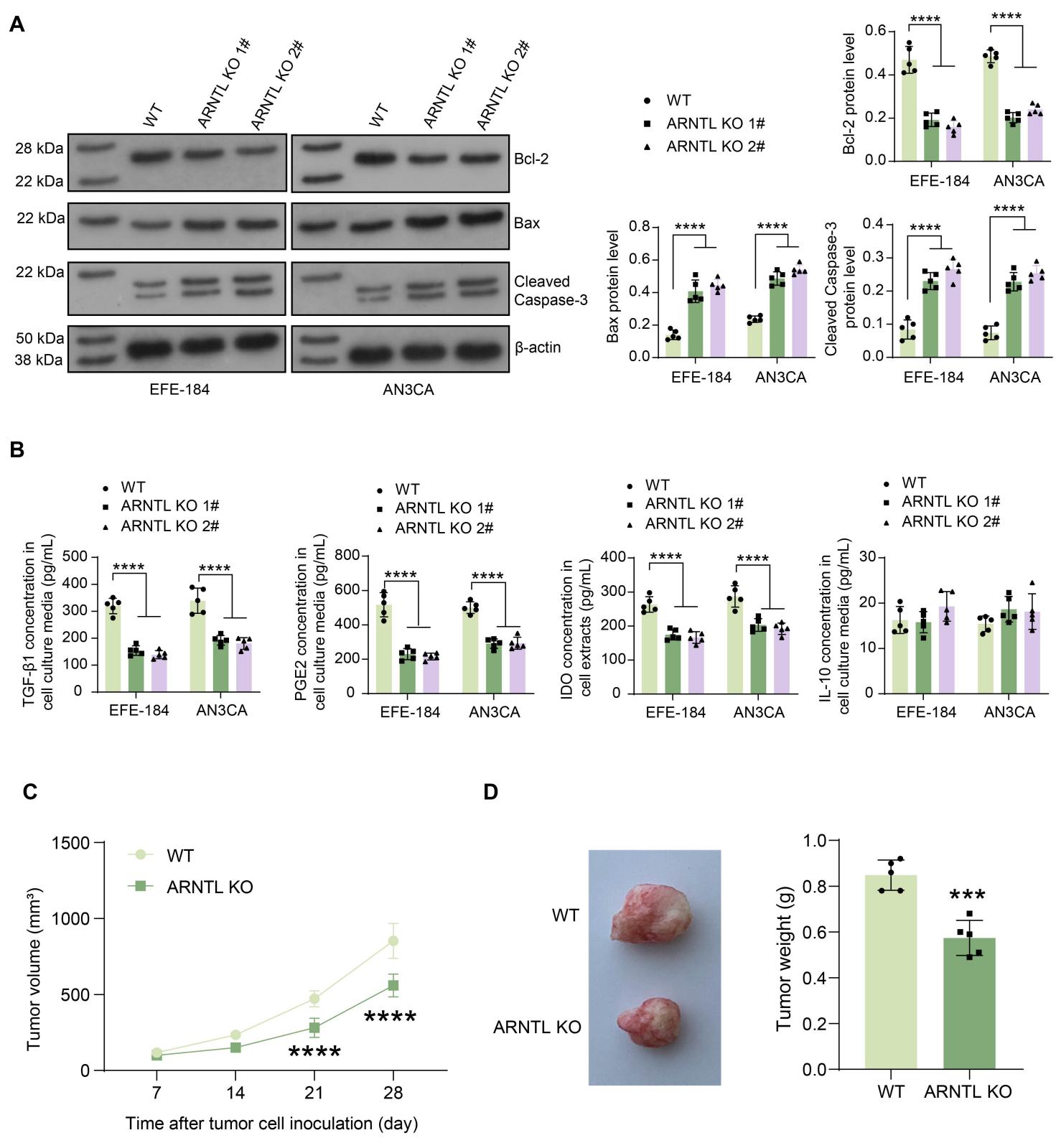
**

**Fig S1** Effect of ARNTL KO on EC cells. A, WB detection of Bcl-2, Bax, and Cleaved Caspase-3 protein expression in EC cells; B, the concentrations of TGF-β1, PGE2, and IL-10 released from EC cells and the intracellular IDO concentration by ELISA. WT AN3CA cells or those stably transfected with ARNTL-KO 1# were injected into immunocompromised BALB/c nude mice to generate xenograft tumors. C, the volume of xenograft tumors in mice (n = 5); D, the weight of xenograft tumors on day 28 and the representative images (n = 5). Differences were analyzed by the unpaired *t-test* (D) or two-way ANOVA followed by Sidak's multiple comparisons test (ABC). ****p* < 0.001, *****p* < 0.0001.

**Fig S2**

**
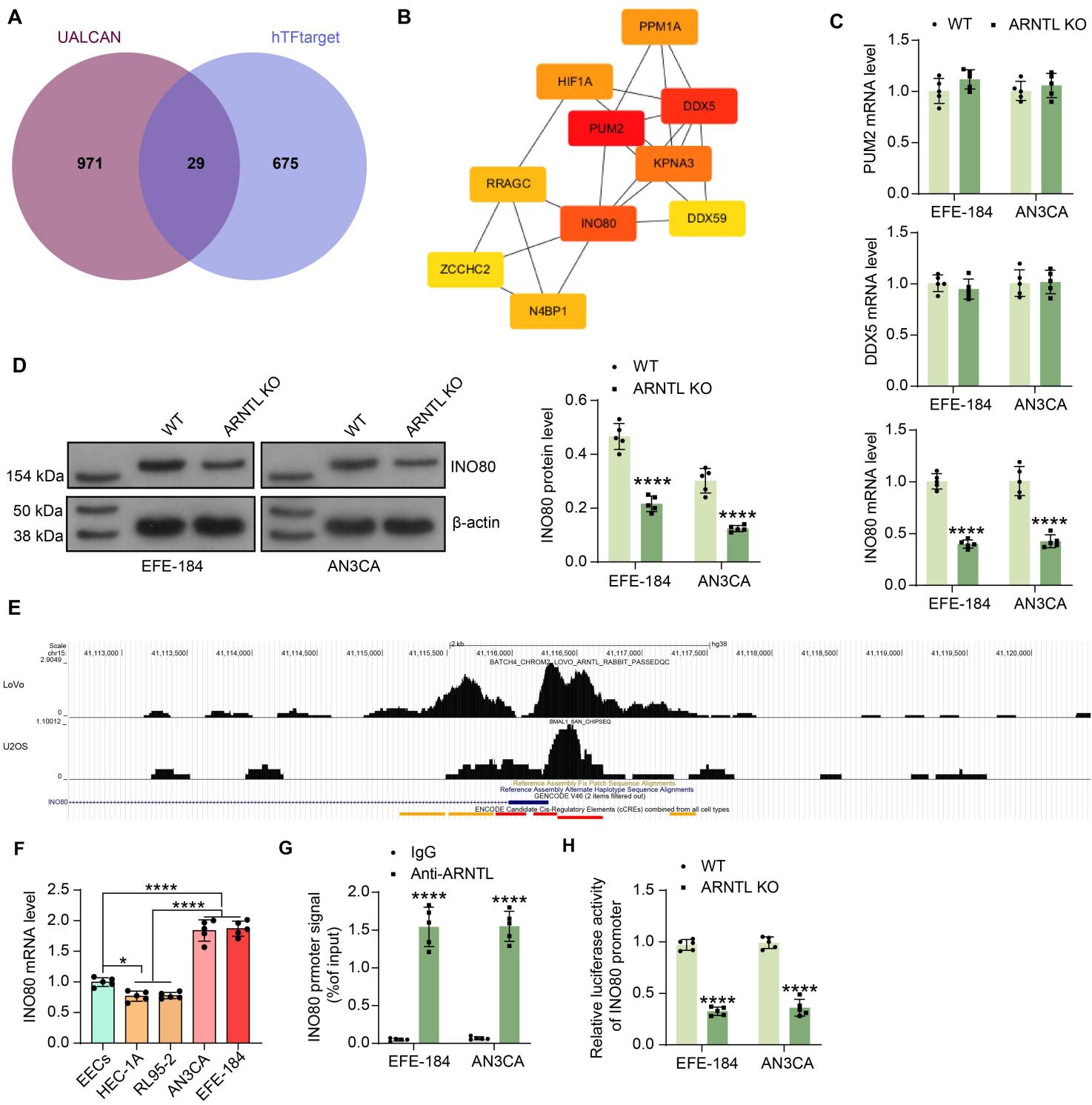
**

**Fig S2** ARNTL knockout reduces INO80 expression in EC cells. A, intersections of genes significantly positively correlated with ARNTL expression in UCEC predicted from the UALCAN system and downstream genes of ARNTL predicted from hTFtarget system; B, a PPI network for these genes and the core proteins; C, mRNA expression of the three candidate downstream genes (PUM2, DDX5, and INO80) in ARNTL-KO cells determined using RT-qPCR; D, the protein level of INO80 in ARNTL-KO cells determined using WB analysis; E, binding of ARNTL to the INO80 promoter region predicted from the ARNTL ChIP-seq data; F, INO80 mRNA expression in EECs and EC cell lines (HEC-1A, RL95-2, EFE-184, and AN3CA) determined by RT-qPCR; G, binding relationship between ARNTL and the INO50 promoter examined using ChIP-qPCR assay; H, regulation of ARNTL on the transcription activity of the INO80 promoter determined using the dual luciferase reporter assay. All cellular experiments were performed in five biological replicates. Differences were analyzed by the one-way (F) or two-way (C, D, G, and H) ANOVA, followed by Tukey's or Sidak's multiple comparisons test. **p* < 0.05, *****p* < 0.0001.

**Fig S3**

**
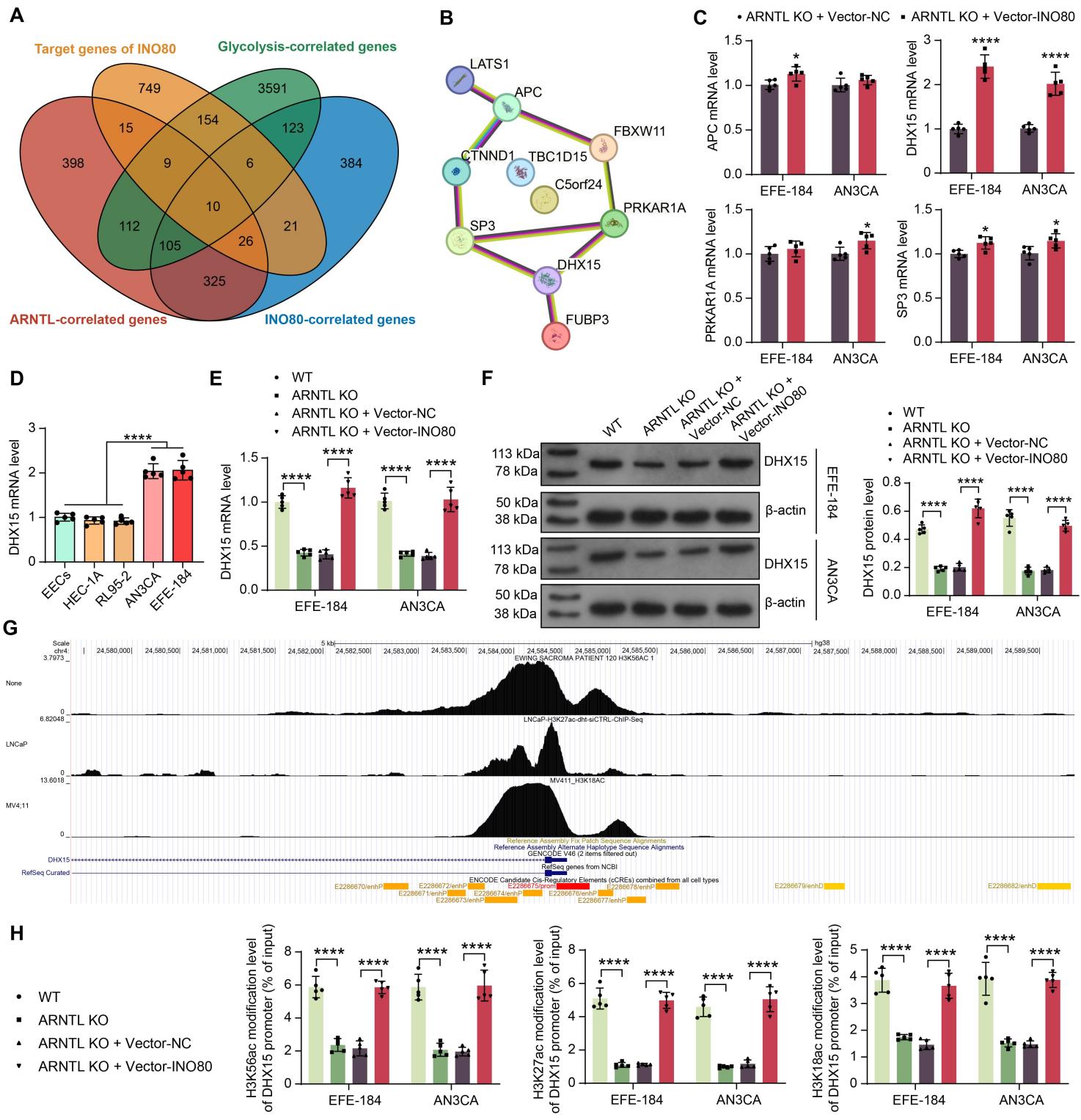
**

**Fig S3** The ARNTL-INO80 axis modulates DHX15 expression. A, intersections of genes significantly correlated with ARNTL and INO80 expression in UCEC predicted from UALCAN system, top 1000 target genes of INO80 predicted from hTFtarget, and glycolysis-related genes downloaded from GeneCards system; B, a PPI network for these intersecting genes and the core proteins; C, mRNA expression of the four candidate downstream genes (APC, DHX15, PRKAR1A, and SP3) in EC cells overexpressing INO80 determined using RT-qPCR; D, DHX15 mRNA expression in EECs and EC cell lines (HEC-1A, RL95-2, EFE-184, and AN3CA) determined by RT-qPCR; E-F, mRNA (E) and protein (F) levels of DHX15 in EFE-184 and AN3CA cells after ARNTL KO and INO80 overexpression determined using RT-qPCR and WB analysis, respectively; G, significant histone acetylation modifications near the DHX15 promoter region according to the ChIP-seq data; H, histone acetylation marker (H3K56ac, H3K27ac, H3K18ac) levels near the DHX15 promoter in EC cells determined using ChIP-qPCR assay. All cellular experiments were performed in five biological replicates. Differences were analyzed by the one-way (D) or two-way (C, E, F, and H) ANOVA, followed by Sidak's or Tukey's multiple comparisons test. **p* < 0.05, *****p* < 0.0001.

**Fig S4**

**
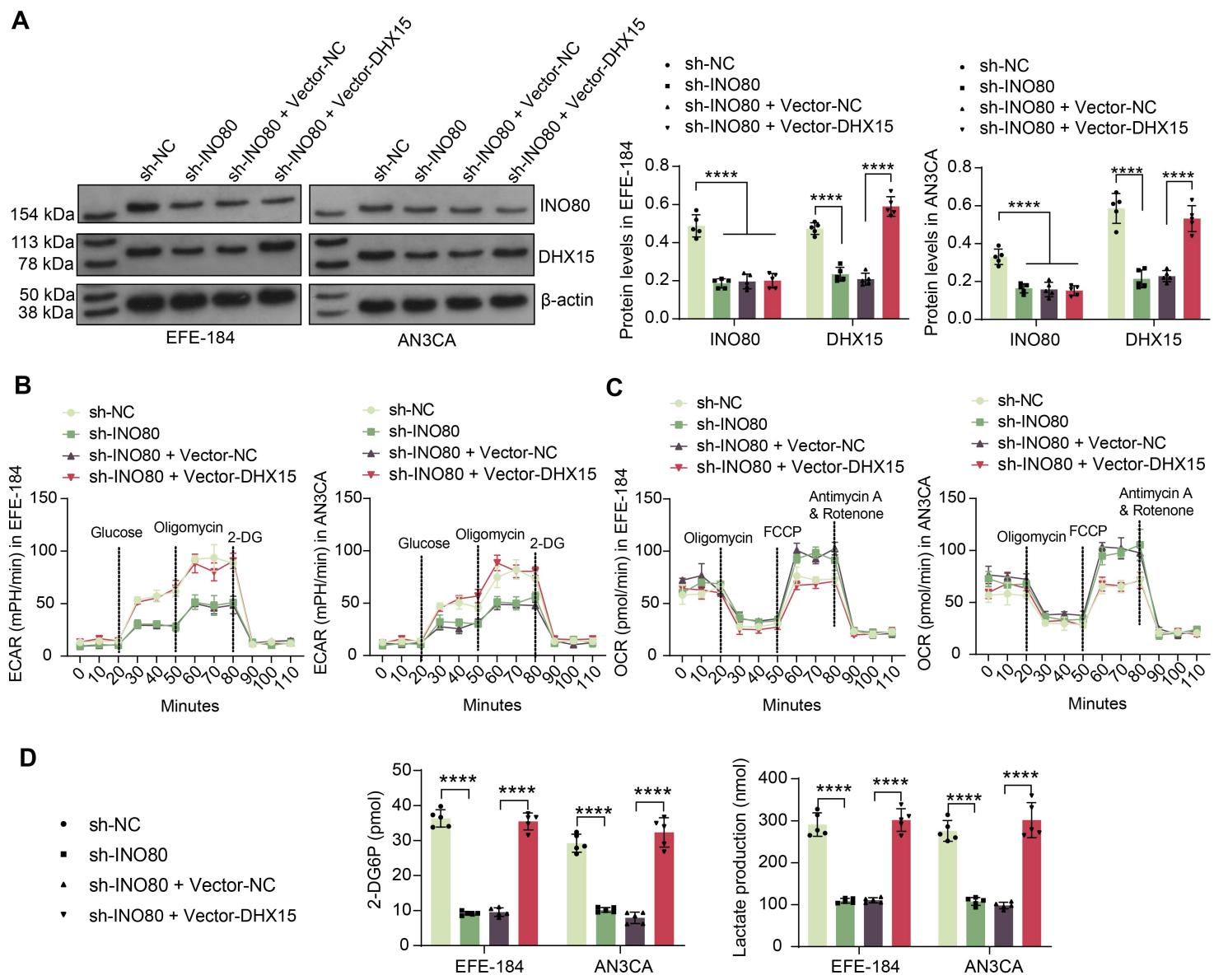
**

**Fig S4** Overexpression of DHX15 restores glycolytic reprogramming in EC cells suppressed by INO80 silencing. EFE-184 and AN3CA were administered lentivirus vectors-encapsulated sh-INO80 and Vector-DHX15. A, protein levels of DHX15 and INO80 in cells determined by WB analysis; B, glycolytic activity in cells evaluated by ECAR; C, mitochondrial respiration in cells evaluated by OCR; D, glucose uptake and lactate production in cells determined by colorimetry. All cellular experiments were performed in five biological replicates. Differences were analyzed by two-way ANOVA followed by Tukey's multiple comparisons test (A and D). *****p* < 0.0001.

**Fig S5**


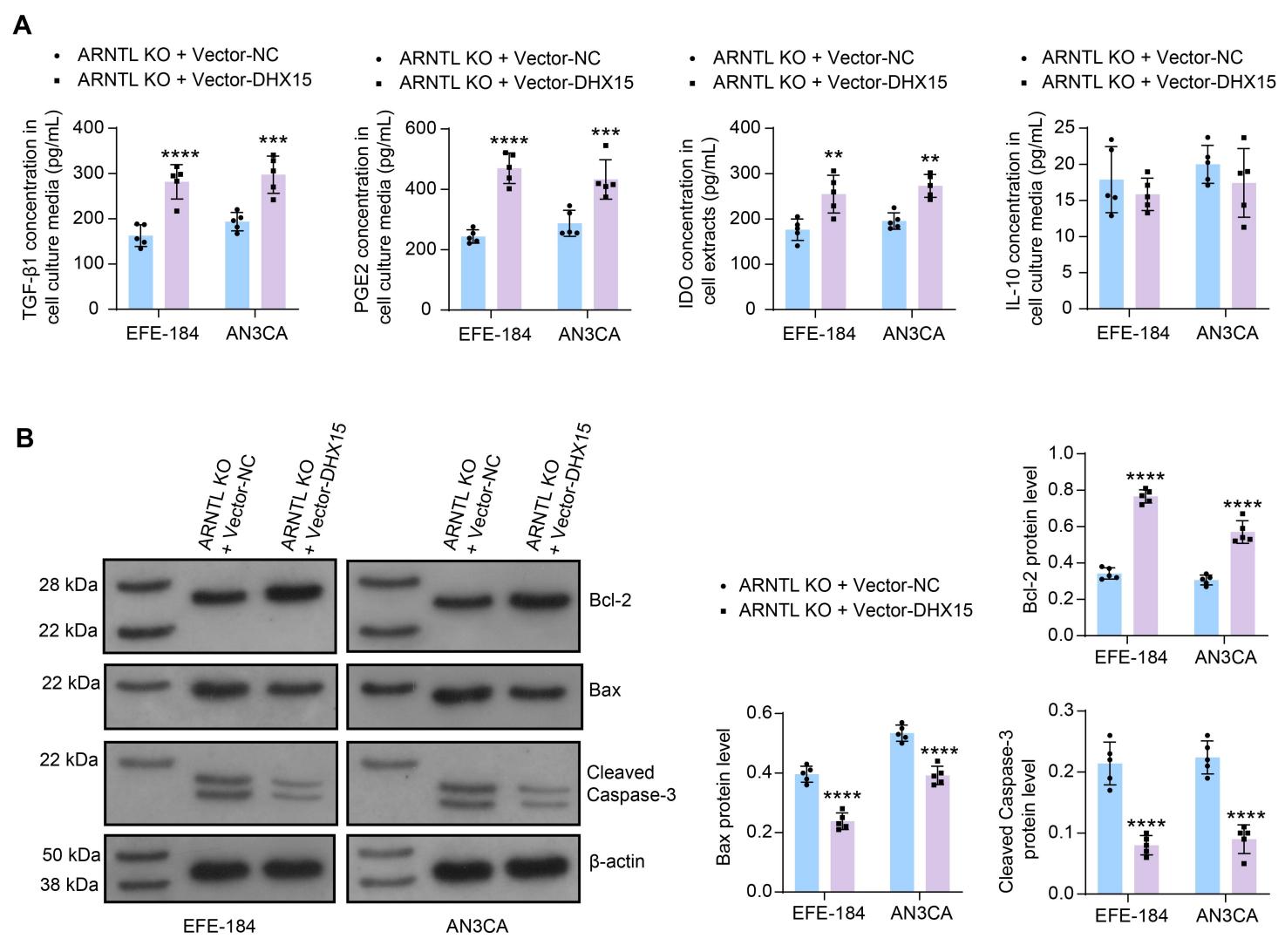


**Fig S5** DHX15 regulates the expression of immunosuppressive molecules and apoptosis-related proteins in EC cells. A, TGF-β1, PGE2, IL-10 releases, and intracellular IDO content in EC cells by ELISA; B, intracellular Bcl-2, Bax, and Cleaved Caspase-3 protein expression detected by WB. All cellular experiments were performed in five biological replicates. Differences were analyzed by two-way ANOVA followed by Tukey's multiple comparisons test (A and B). ***p* < 0.01, ****p* < 0.001, *****p* < 0.0001.
