# Supplementary material for: ARNTL-mediated INO80-DHX15 axis reprograms the glycolytic metabolism and augments the progression of endometrial carcinoma
Source: Cell Death Dis. 2025 Jun 20;16(1):463. doi: 10.1038/s41419-025-07776-w (PMC12181345; doi:10.1038/s41419-025-07776-w)
Supplement: Supplementary file 2 — Table S1: Primer sequences for RT-qPCR [file 41419_2025_7776_MOESM2_ESM.docx]

**Table S1**: Primer sequences for qPCR

| Gene symbol | Forward primer (5ʹ-3ʹ) | Reverse primer (5ʹ-3ʹ) |
| --- | --- | --- |
| ARNTL (Exon 13:1504bp-1747bp) | CCACAAAGATGGGGCTGGAT | CACCCTGATTTCCCCGTTCA |
| PUM2 | CGGTTAATGGCTCCAACACCTG | CGAAACAGACCATTTGTGCTGCC |
| DDX5 | GCTTCCTGCCATTGTCCACATC | GCAGCTACTTGCTGCACCTGTT |
| INO80 | CCACCAGAGATACCTGAGGAAC | CAACCTGGTCTGAGTAGCCACT |
| APC | AGGCTGCATGAGAGCACTTGTG | CACACTTCCAACTTCTCGCAACG |
| DHX15 | CACTGCTGAACGTCTACCATGC | CATTGTCTGCGGACATCAGGGA |
| PRKAR1A | TATGGAACACCGAGAGCAGCCA | CATCTTCCGCTTTCTCAGTGTGC |
| SP3 | TGTCCCAACTGTAAAGAAGGTGG | CTCCAGAATGCCAACGCAGATG |
| IL-6 | AGACAGCCACTCACCTCTTCAG | TTCTGCCAGTGCCTCTTTGCTG |
| TNF-α | CTCTTCTGCCTGCTGCACTTTG | ATGGGCTACAGGCTTGTCACTC |
| IFN-γ | GAGTGTGGAGACCATCAAGGAAG | TGCTTTGCGTTGGACATTCAAGTC |
| PDL-1 | TGCCGACTACAAGCGAATTACTG | CTGCTTGTCCAGATGACTTCGG |
| IL-10 | TCTCCGAGATGCCTTCAGCAGA | TCAGACAAGGCTTGGCAACCCA |
| INO80 promoter | GCACTCTCTTGACCTCACCT | GTCAACGGGAAAGTAGTGCG |
| DHX15 promoter | GCCGGAACCAACAGCTAAAA | AGCTACAGGAAGAGGGAGGA |
| β-actin | CACCATTGGCAATGAGCGGTTC | AGGTCTTTGCGGATGTCCACGT |

**Note:** qPCR, quantitative polymerase chain reaction; ARNTL, aryl hydrocarbon receptor nuclear translocator-like; PUM2, pumilio RNA binding family member 2; DDX5, DEAD-box helicase 5; INO80, INO80 complex ATPase subunit; APC, APC regulator of WNT signaling pathway; DHX15, DEAH-box helicase 15; PRKAR1A, protein kinase a regulatory subunit I alpha; SP3, Sp3 transcription factor; IL, interleukin; TNF-α, tumor necrosis factor-α; IFN-γ, interferon-γ; PDL-1, programmed cell death ligand 1.
