# Supplementary material for: ARNTL-mediated INO80-DHX15 axis reprograms the glycolytic metabolism and augments the progression of endometrial carcinoma
Source: Cell Death Dis. 2025 Jun 20;16(1):463. doi: 10.1038/s41419-025-07776-w (PMC12181345; doi:10.1038/s41419-025-07776-w)
Supplement: Supplementary file 3 — Table S2: Correlation between ARNTL expression and the clinical parameters of patients with EC (N = 300) [file 41419_2025_7776_MOESM3_ESM.docx]

**Table S2:** Correlation between ARNTL expression and the clinical parameters of patients with EC (N = 300)

| Clinical parameters | | ARNTL expression | | | Chi-square | *P* value |
| --- | --- | --- | --- | --- | --- | --- |
|  |  | Strong (N = 49) | Moderate (N = 208) | Weak (N = 43) |  |  |
| Age (years) | > 50 | 30 | 150 | 29 | 2.343 | 0.3098 |
|  | ≤ 50 | 19 | 58 | 14 |  |  |
| CA125 (U/mL) | > 40 | 36 | 22 | 1 | 108.8 | < 0.0001 |
|  | ≤ 40 | 13 | 186 | 42 |  |  |
| Tumor differentiation grade | Ⅰ | 23 | 123 | 38 | 69.46 | < 0.0001 |
|  | Ⅱ | 10 | 37 | 4 |  |  |
|  | Ⅲ | 16 | 8 | 0 |  |  |
|  | Non-endometrioid carcinoma | 0 | 40 | 1 |  |  |
| TNM Stage | Ⅰ | 21 | 149 | 39 | 27.59 | 0.0001 |
|  | Ⅱ | 12 | 22 | 2 |  |  |
|  | Ⅲ | 13 | 33 | 2 |  |  |
|  | IV | 3 | 4 | 0 |  |  |
| Lymph node metastasis | Positive | 12 | 26 | 1 | 10.1 | 0.0064 |
|  | Negative | 37 | 182 | 42 |  |  |
| Ki67-positive cells | > 40% | 26 | 103 | 5 | 22.37 | < 0.0001 |
|  | ≤ 40% | 23 | 105 | 38 |  |  |
| ER status | Negative | 7 | 26 | 2 | 2.52 | 0.2837 |
|  | Positive | 42 | 182 | 41 |  |  |
| PR status | Negative | 14 | 47 | 5 | 3.972 | 0.1372 |
|  | Positive | 35 | 161 | 38 |  |  |
| p53 status | Negative | 0 | 6 | 0 | 9.412 | 0.0516 |
|  | Wild type | 46 | 161 | 38 |  |  |
|  | Mutant type | 3 | 41 | 5 |  |  |

Note: Chi-square test was used to analyze the enumeration data and to calculate the association between every two categorical variables. A *p*-value less than 0.05 was statistically significant. ARNTL, aryl hydrocarbon receptor nuclear translocator-like; EC, endometrial cancer; TNM, tumor node metastasis
