# Supplementary material for: ARNTL-mediated INO80-DHX15 axis reprograms the glycolytic metabolism and augments the progression of endometrial carcinoma
Source: Cell Death Dis. 2025 Jun 20;16(1):463. doi: 10.1038/s41419-025-07776-w (PMC12181345; doi:10.1038/s41419-025-07776-w)

Fig 3G

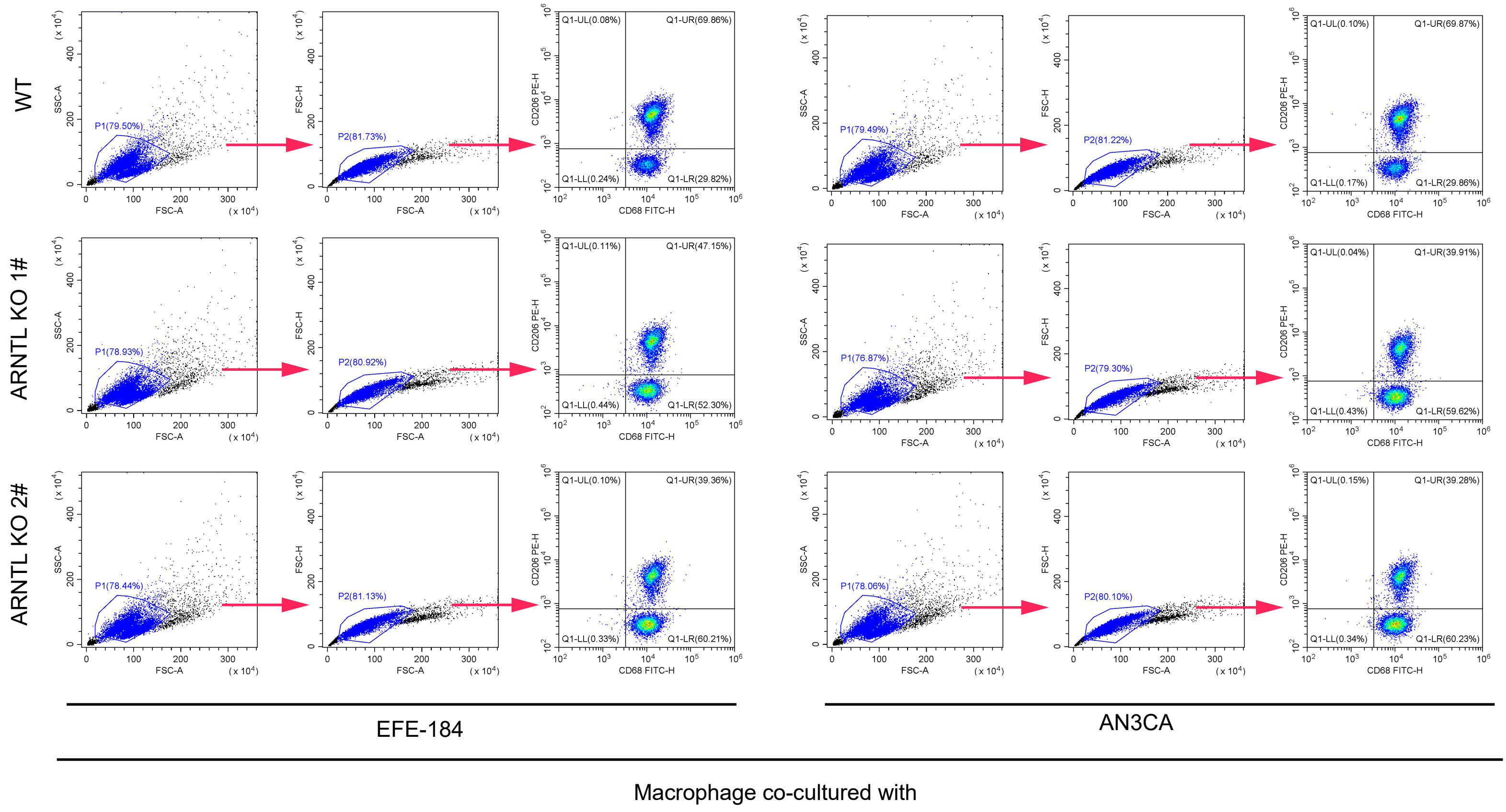
WT
ARNTL KO 1#
ARNTL KO 2#
SSC-A
FSC-A
FSC-H
CD206 PE-H
CD68 FITC-H
P1(79.50%)
P2(81.73%)
Q1-UL(0.08%)
Q1-UR(69.86%)
Q1-LL(0.24%)
Q1-LR(29.82%)
P1(78.93%)
P2(80.92%)
Q1-UL(0.11%)
Q1-UR(47.15%)
Q1-LL(0.44%)
Q1-LR(52.30%)
P1(78.44%)
P2(81.13%)
Q1-UL(0.10%)
Q1-UR(39.36%)
Q1-LL(0.33%)
Q1-LR(60.21%)
P1(79.49%)
P2(81.22%)
Q1-UL(0.10%)
Q1-UR(69.87%)
Q1-LL(0.17%)
Q1-LR(29.86%)
P1(76.87%)
P2(79.30%)
Q1-UL(0.04%)
Q1-UR(39.91%)
Q1-LL(0.43%)
Q1-LR(59.62%)
P1(78.06%)
P2(80.10%)
Q1-UL(0.15%)
Q1-UR(39.28%)
Q1-LL(0.34%)
Q1-LR(60.23%)
EFE-184
AN3CA
Macrophage co-cultured with

Fig 3J

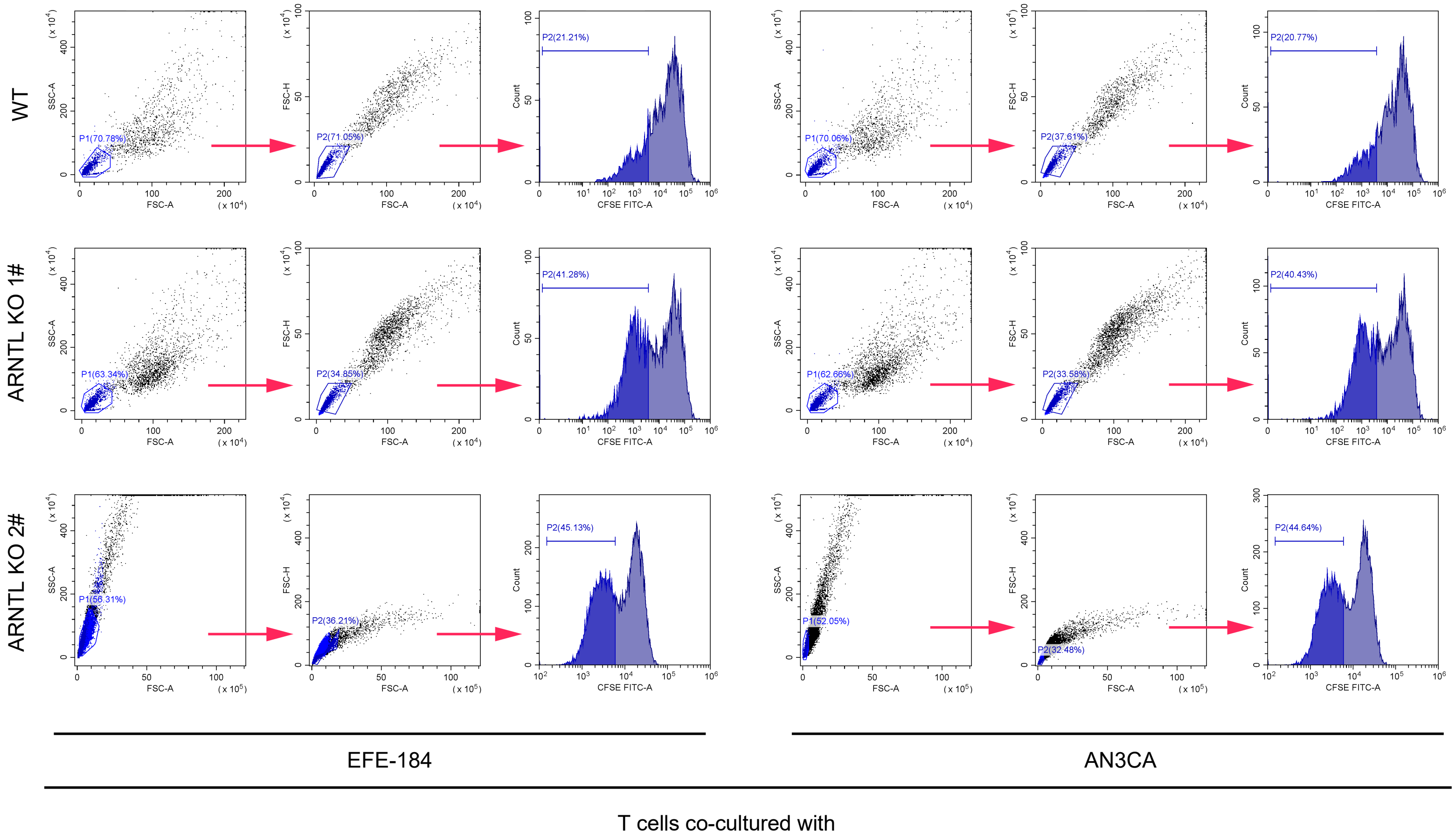
WT
ARNTL KO 1#
ARNTL KO 2#
SSC-A
FSC-A
FSC-H
Count
CFSE FITC-A
P1(70.78%)
P2(71.05%)
P2(21.21%)
P1(70.06%)
P2(37.61%)
P2(20.77%)
P1(63.34%)
P2(34.85%)
P2(41.28%)
P1(62.66%)
P2(33.58%)
P2(40.43%)
P1(56.31%)
P2(36.21%)
P2(45.13%)
P1(52.05%)
P2(32.48%)
P2(44.64%)
EFE-184
AN3CA
T cells co-cultured with

Fig 4D

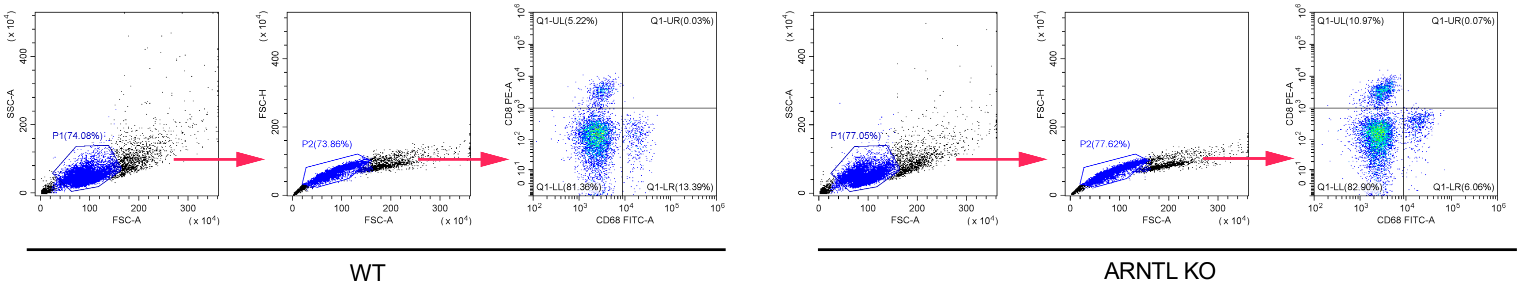

Fig 5H

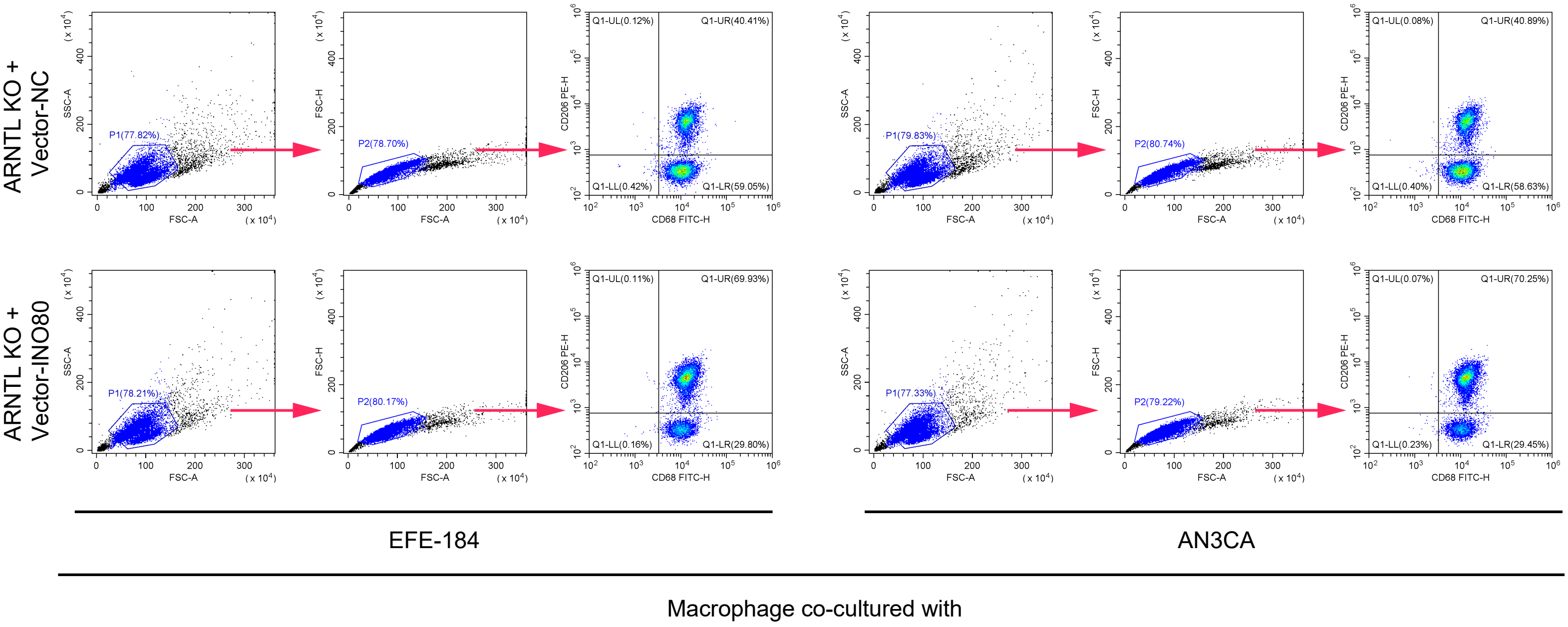

Fig 5J

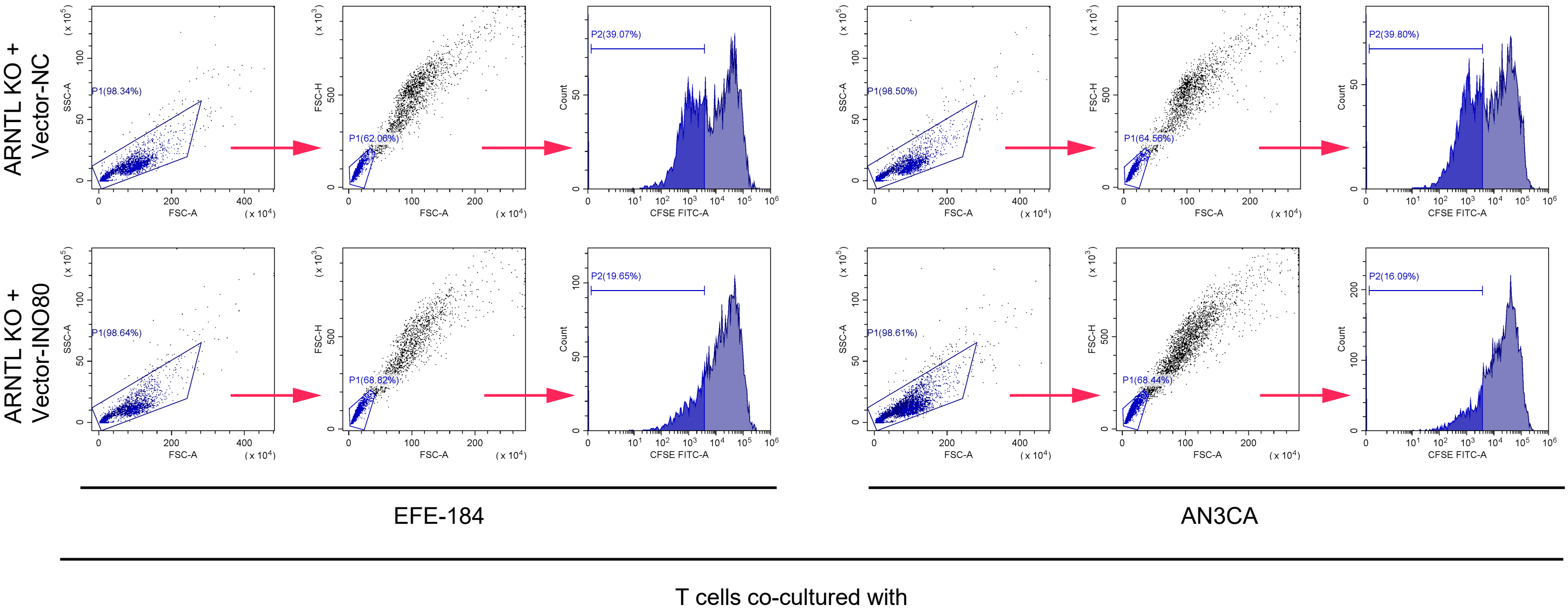

Fig 6E

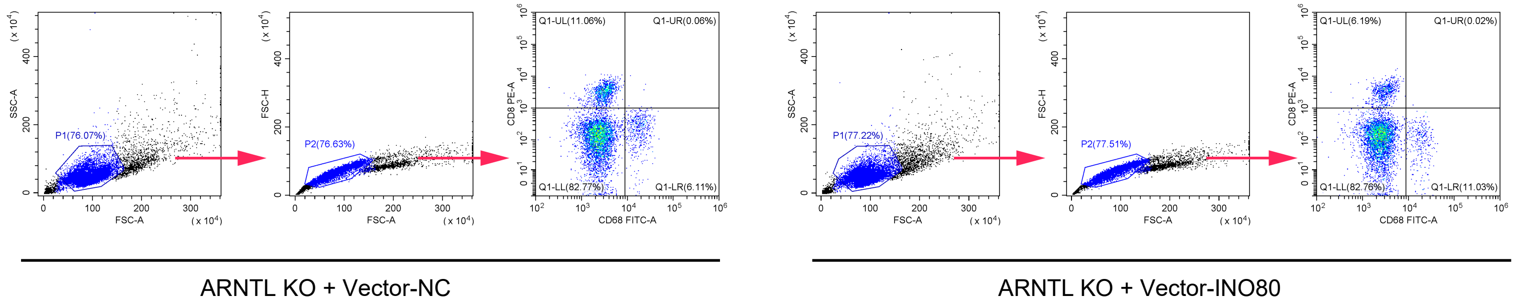

Fig 8A

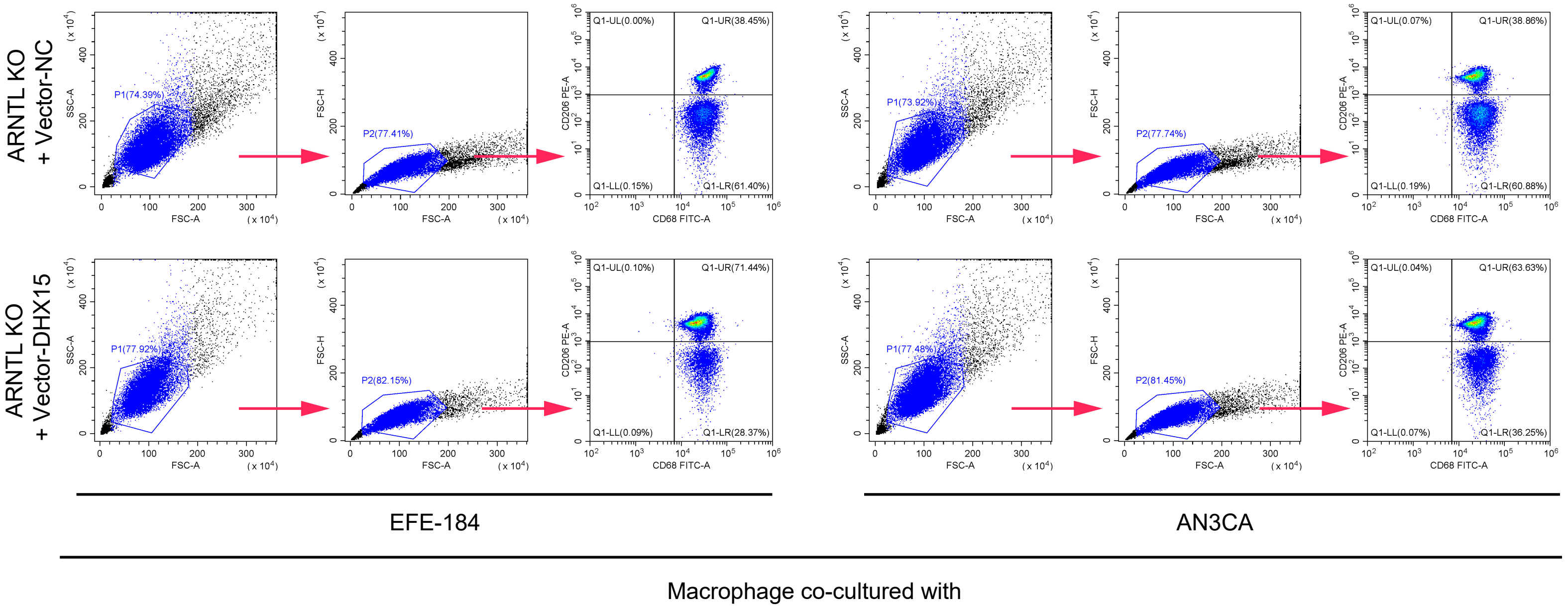

Fig 8B

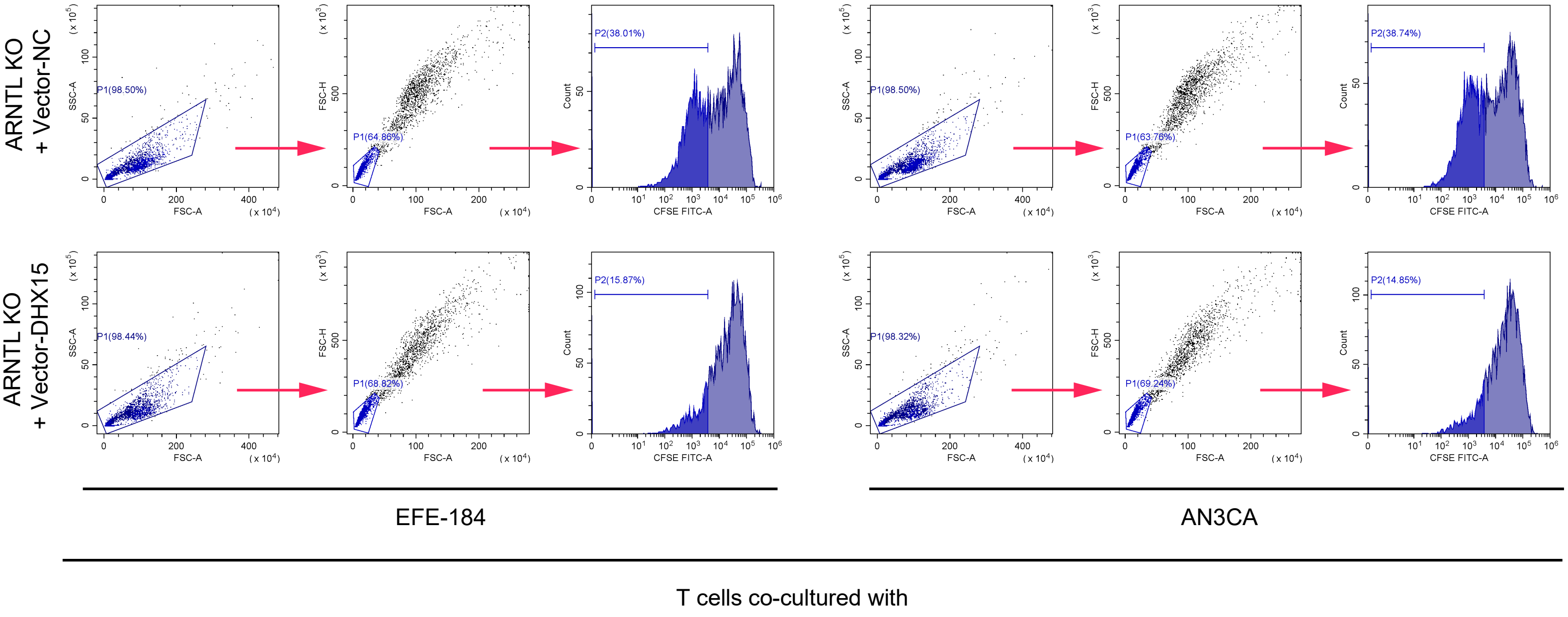

Fig 8G

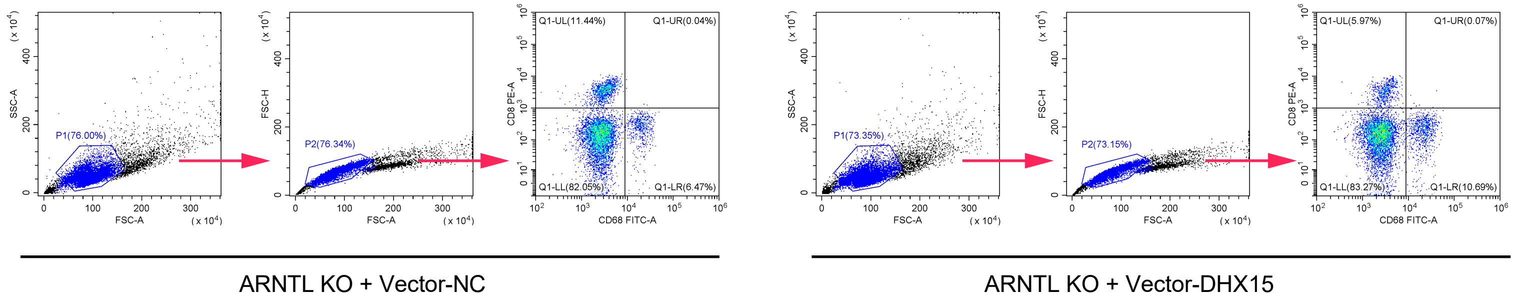

Supplement: Supplementary file 9 — The gating strategies [file 41419_2025_7776_MOESM9_ESM.pdf]
