# Supplementary material for: ARNTL-mediated INO80-DHX15 axis reprograms the glycolytic metabolism and augments the progression of endometrial carcinoma
Source: Cell Death Dis. 2025 Jun 20;16(1):463. doi: 10.1038/s41419-025-07776-w (PMC12181345; doi:10.1038/s41419-025-07776-w)

Original western blots

Fig 3B-1 ARNTL

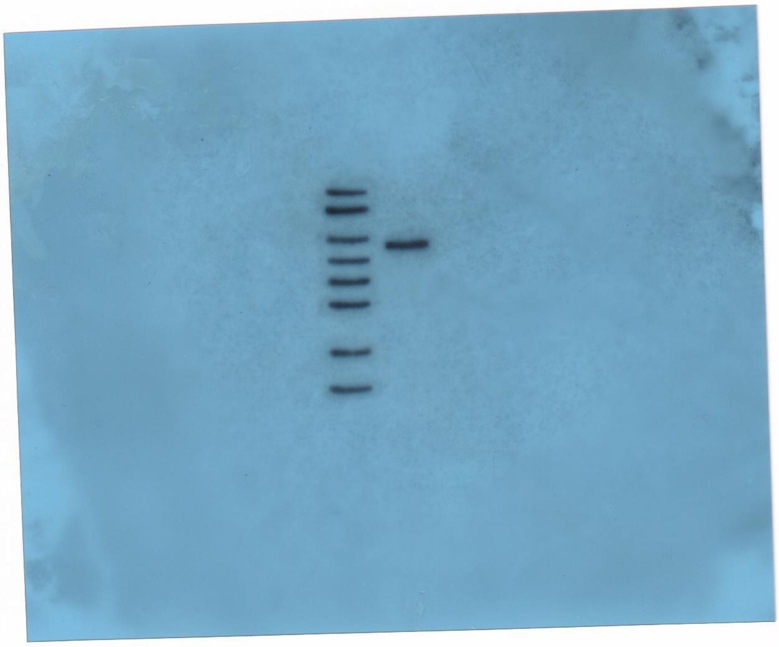

Fig 3B-2 β-actin

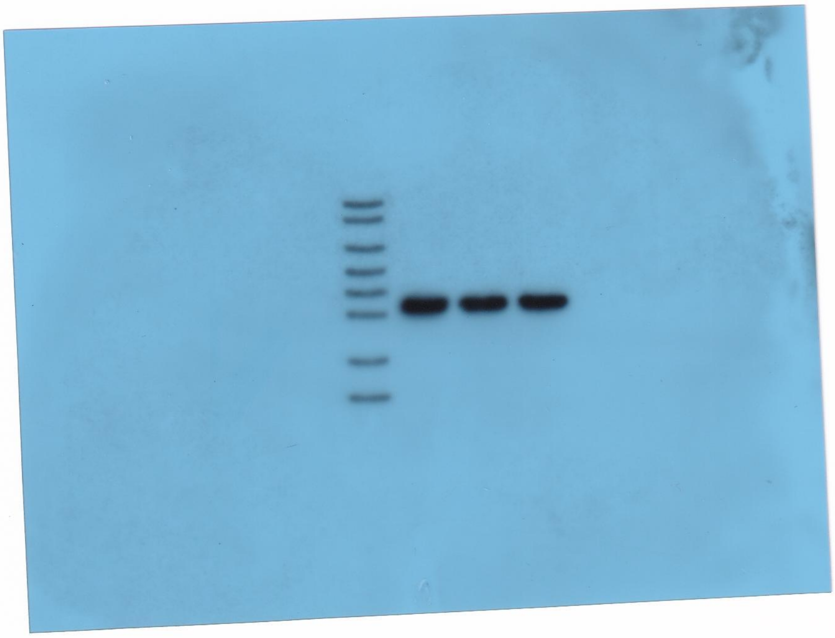

Fig 3B-3 ARNTL

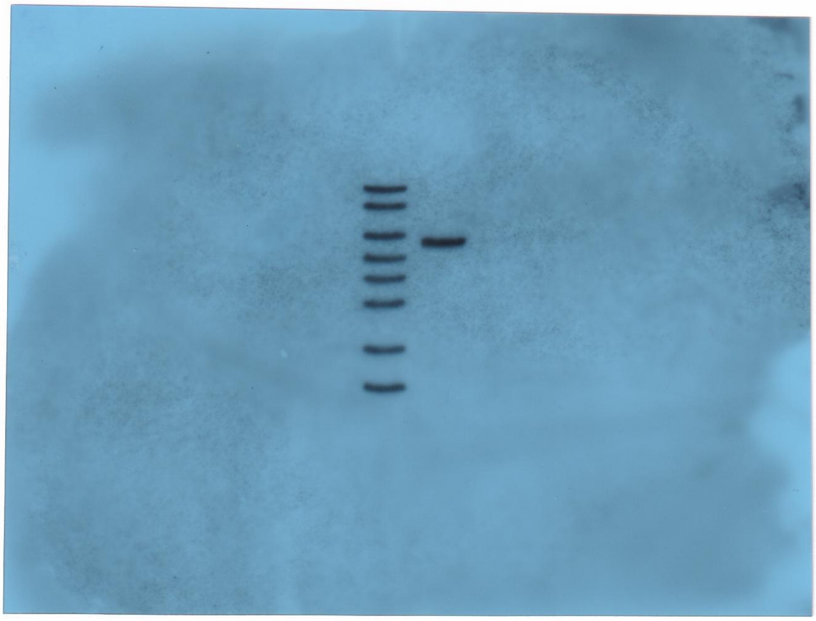

Fig 3B-4 β-actin

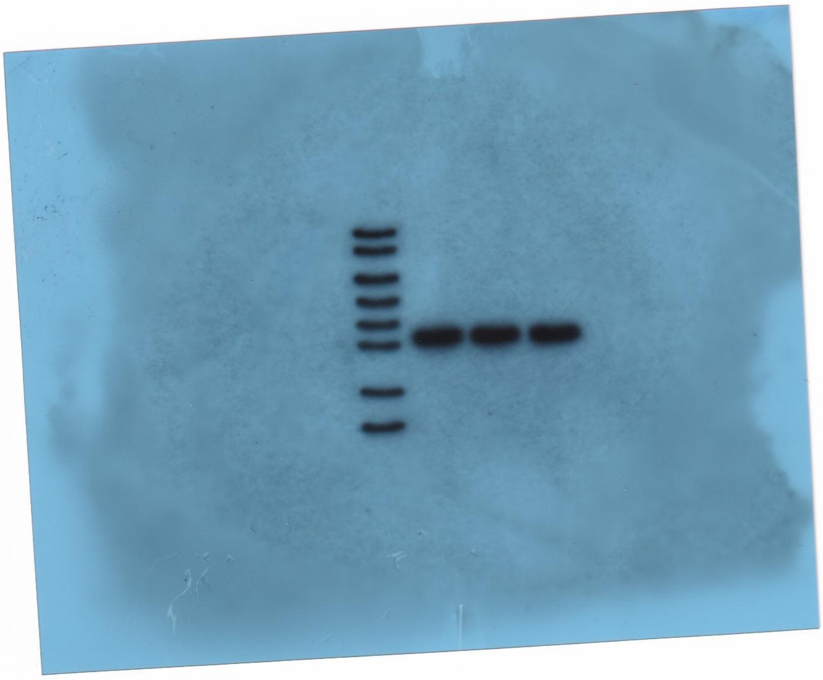

Fig 3I-1 IL-6

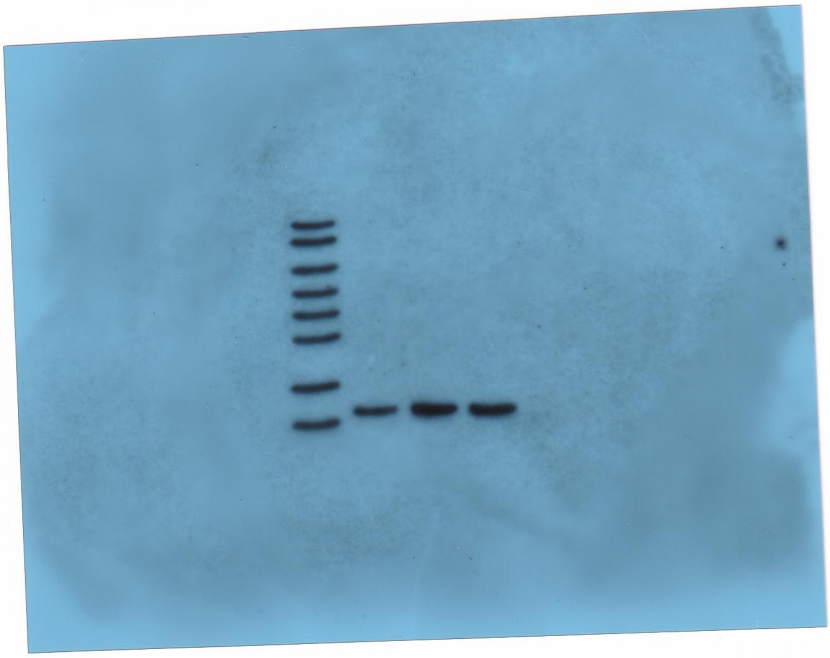

Fig 3I-2 TNF-α

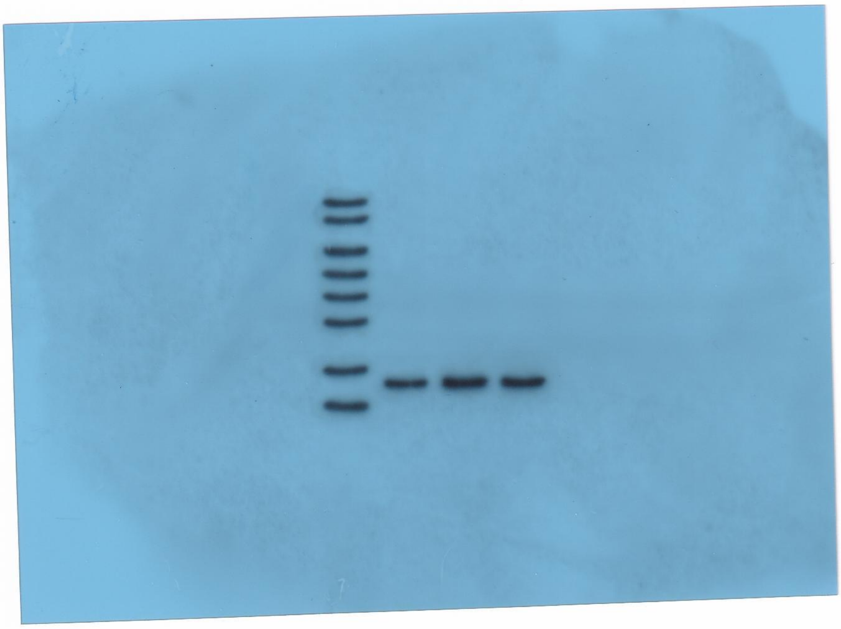

Fig 3I-3 PDL-1

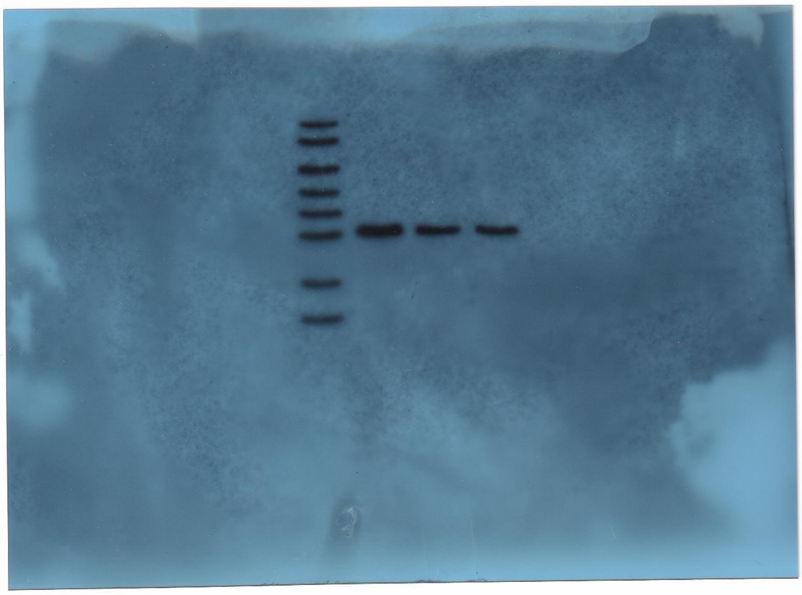

Fig 3I-4 IL-10

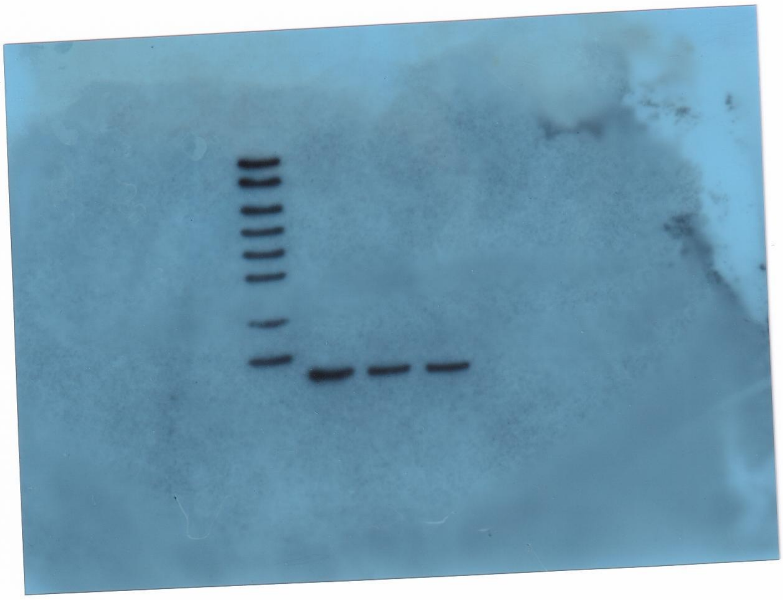

Fig 3I-5 β-actin

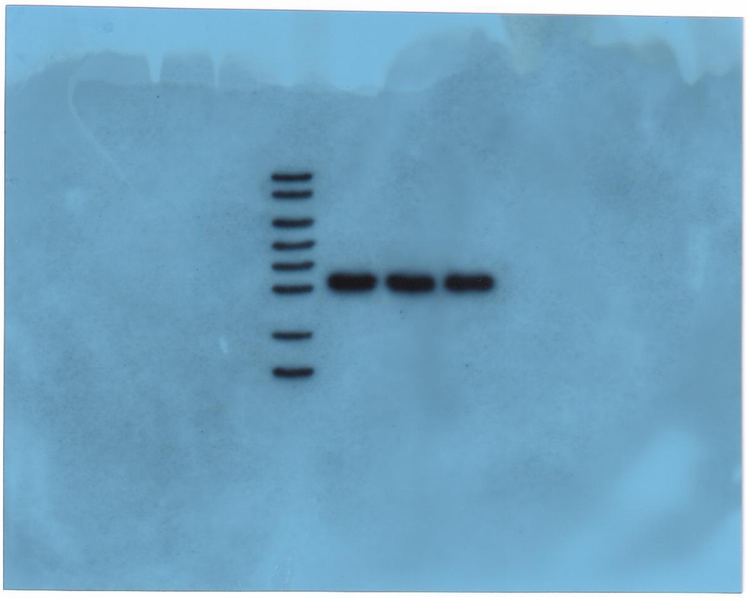

Fig 3I-6 IL-6

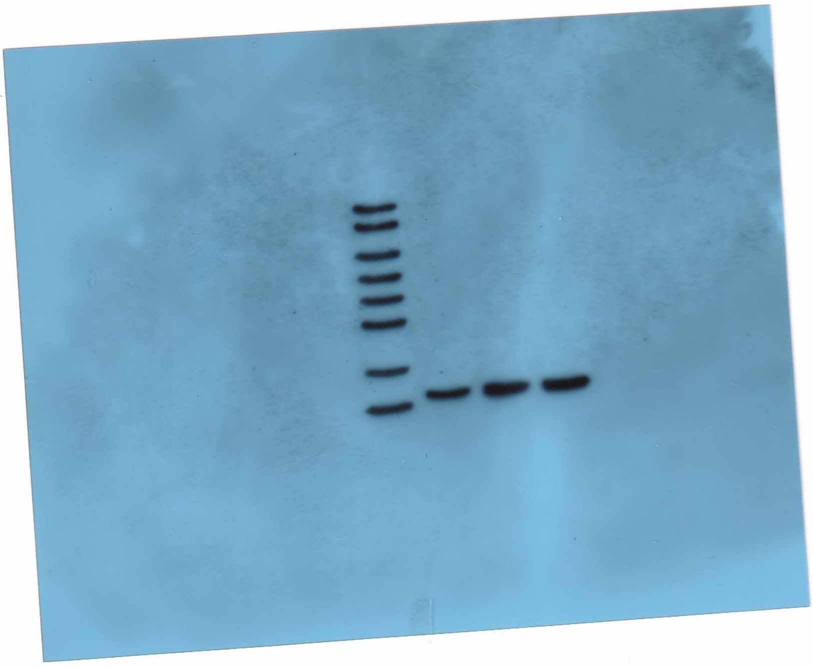

Fig 3I-7 TNF-α

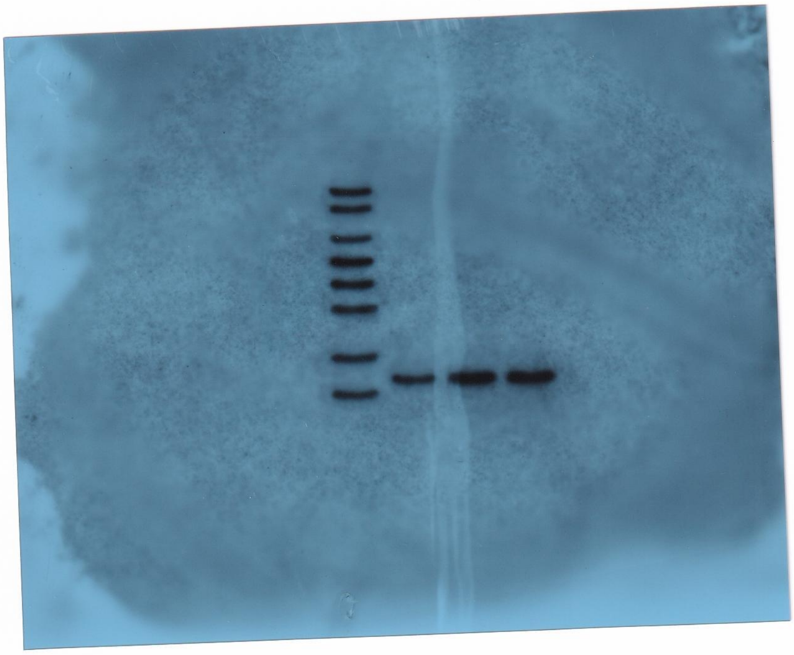

Fig 3I-8 PDL-1

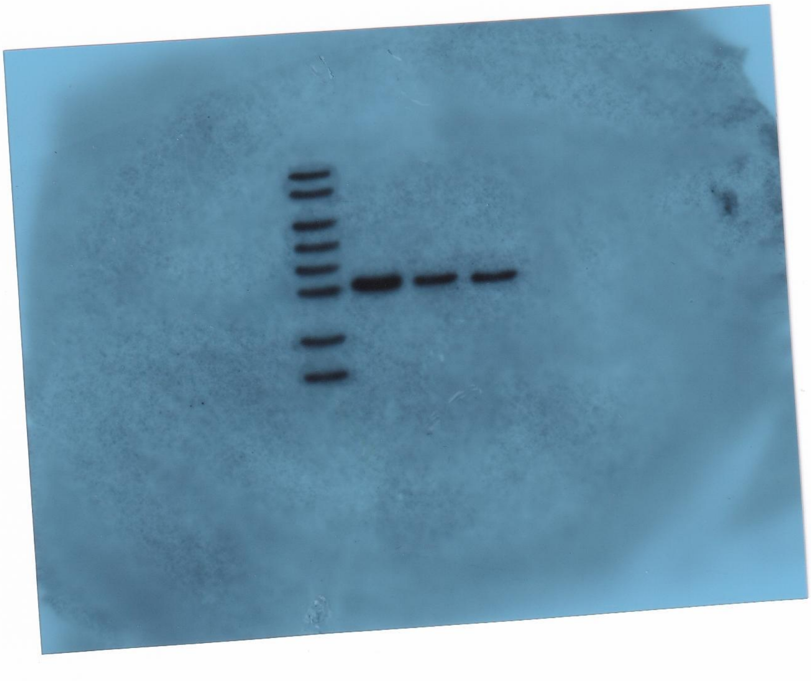

Fig 3I-9 IL-10

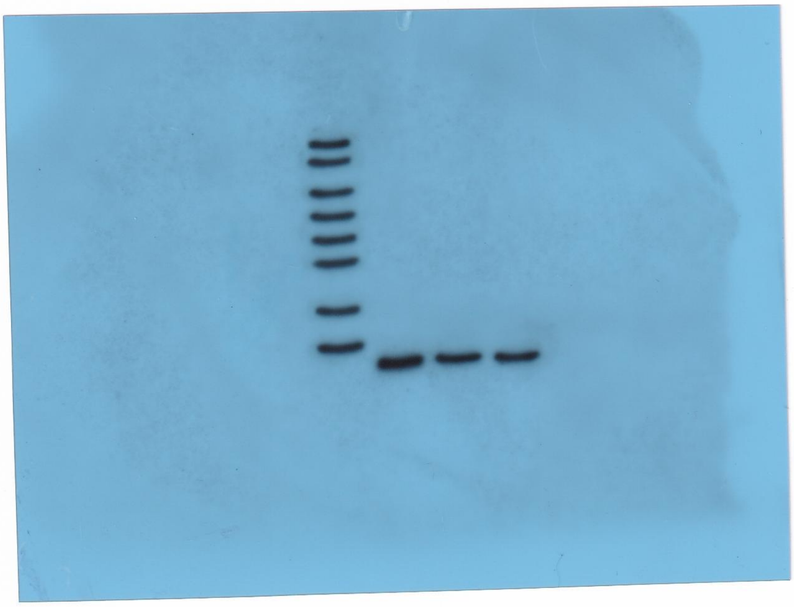

Fig 3I-10 β-actin

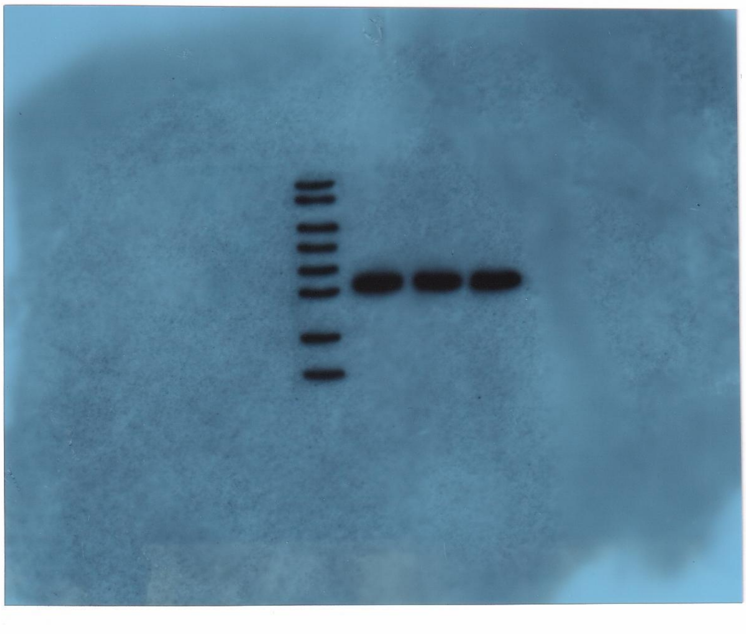

Fig 5B-1 INO80

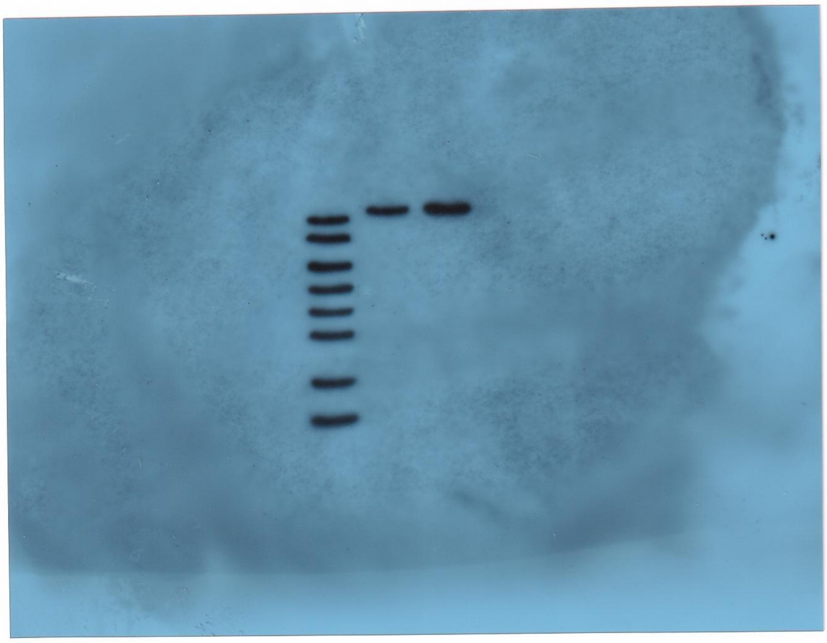

Fig 5B-2 Bcl-2

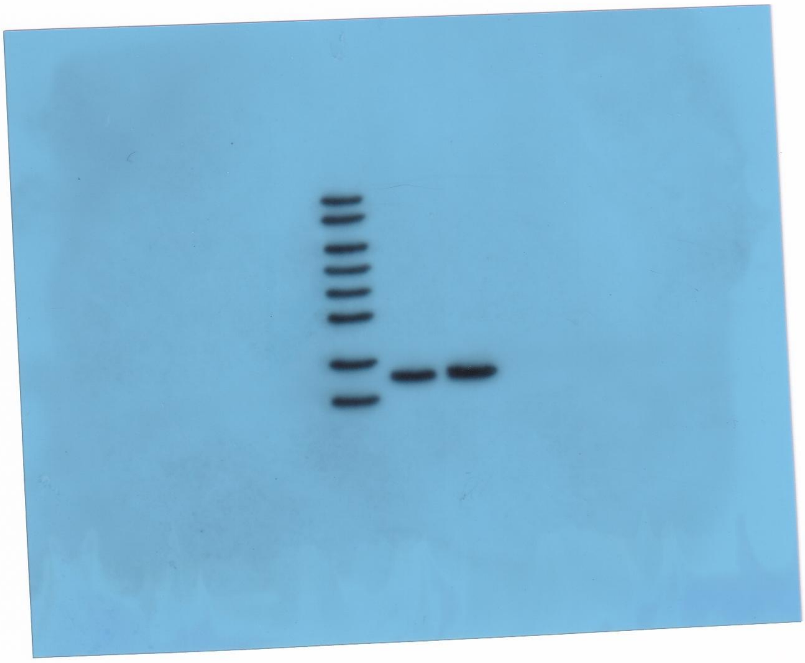

Fig 5B-3 Bax

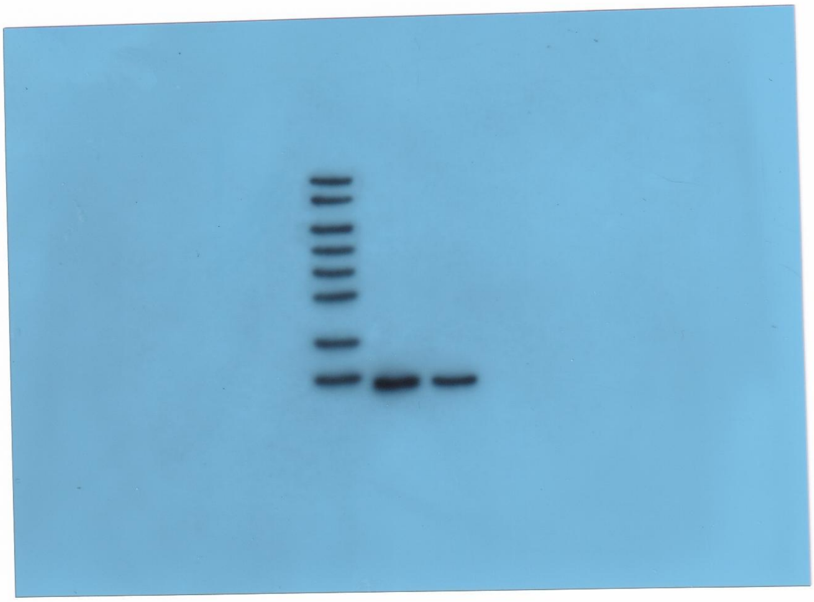

Fig 5B-4 Cleaved Caspase-3

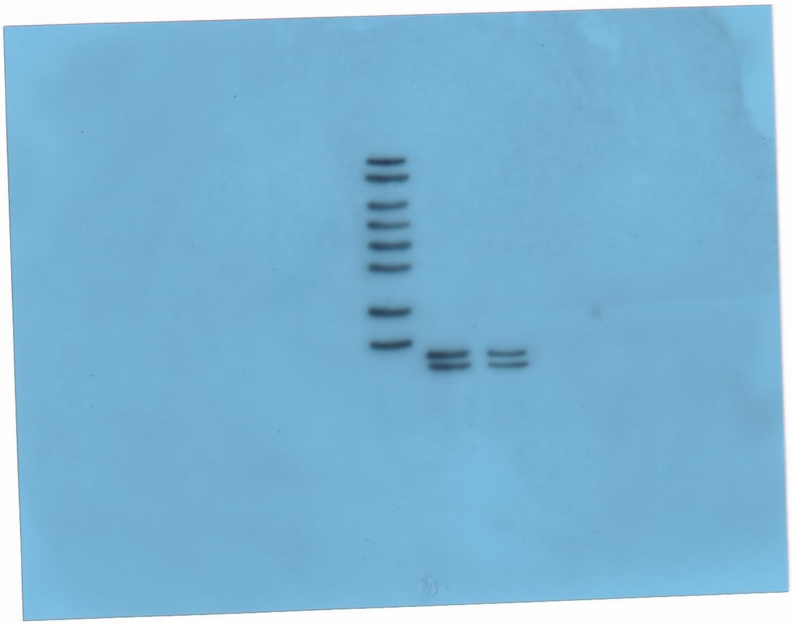

Fig 5B-5 β-actin

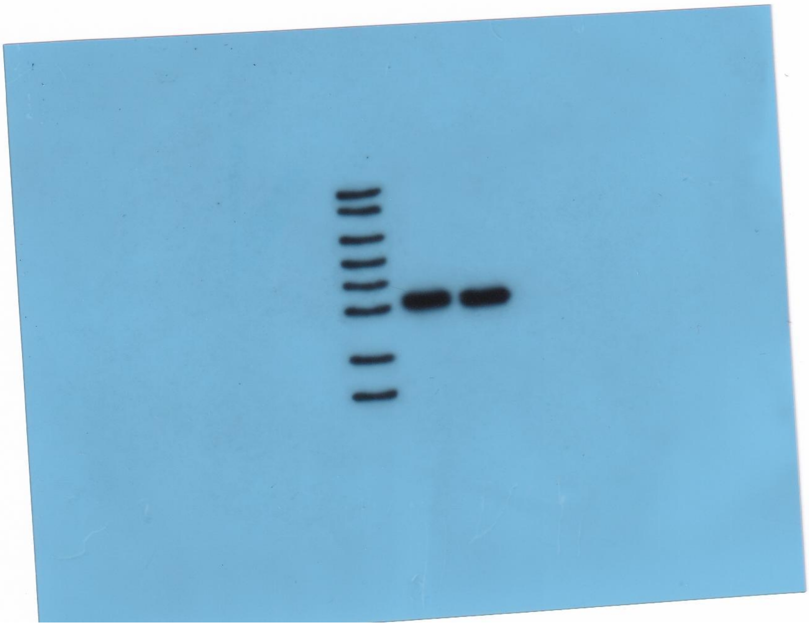

Fig 5B-6 INO80

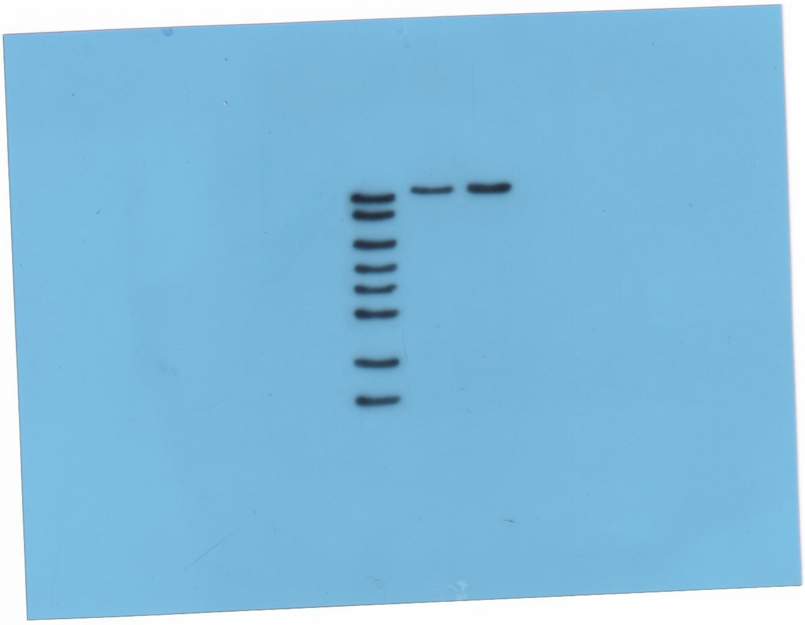

Fig 5B-7 Bcl-2

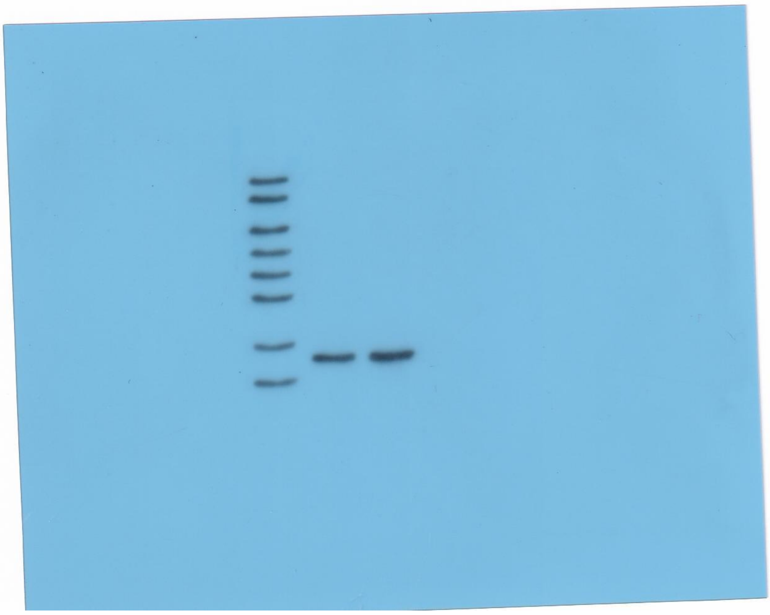

Fig 5B-8 Bax

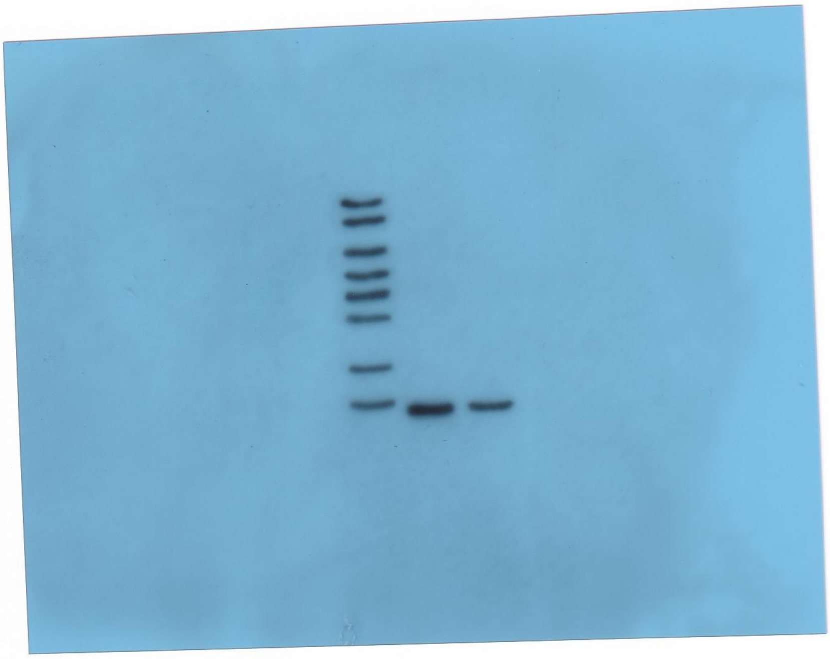

Fig 5B-9 Cleaved Caspase-3

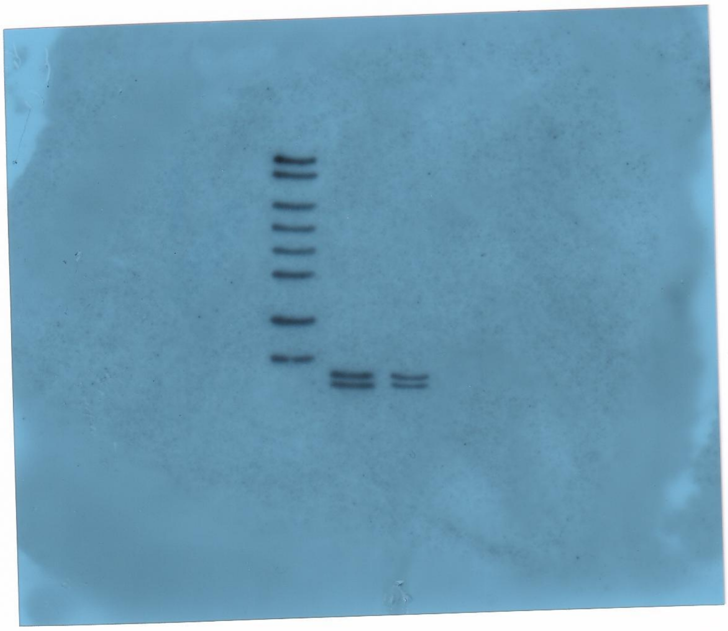

Fig 5B-10 β-actin

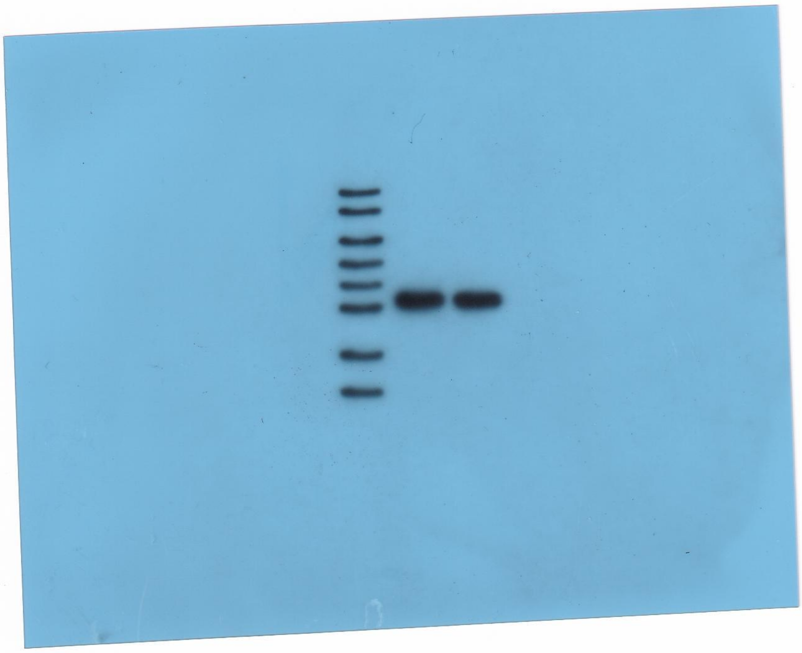

Fig 7D-1 DHX15

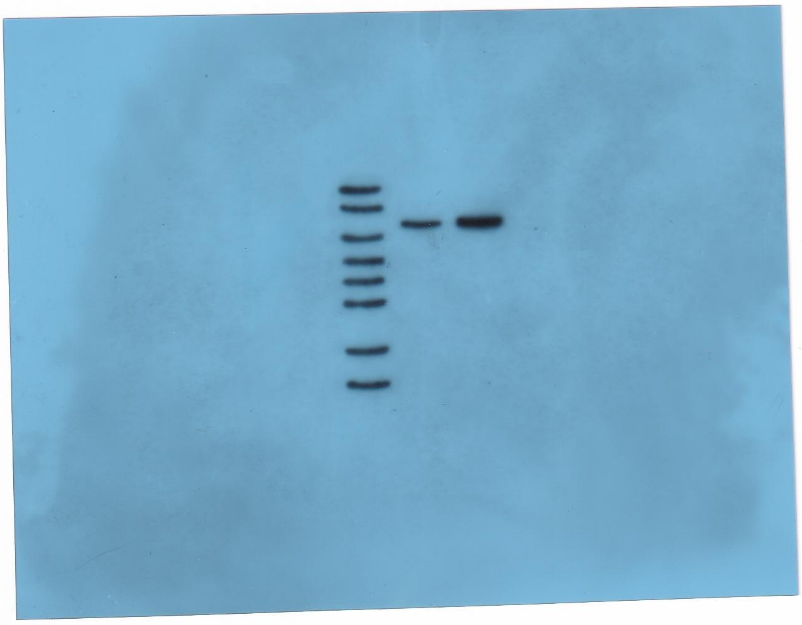

Fig 7D-2 β-actin

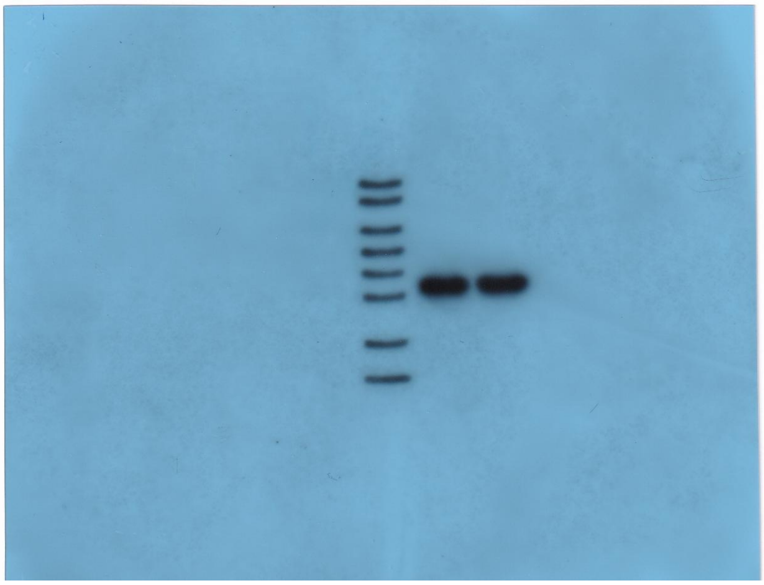

Fig 7D-3 DHX15

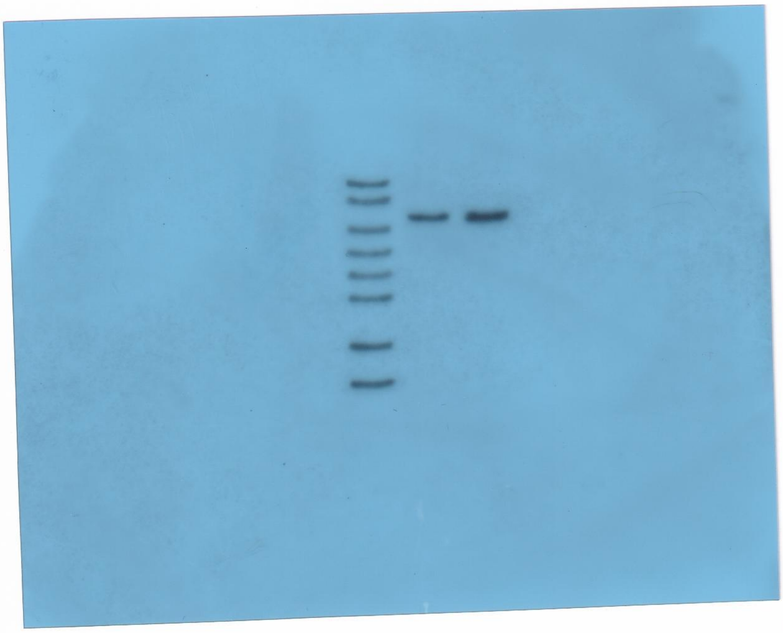

Fig 7D-4 β-actin

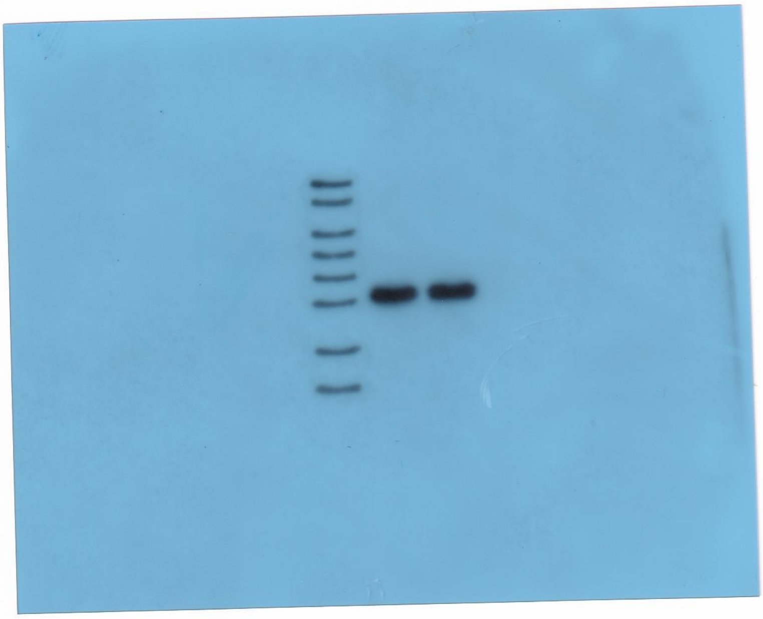

Fig S1A-1 Bcl-2

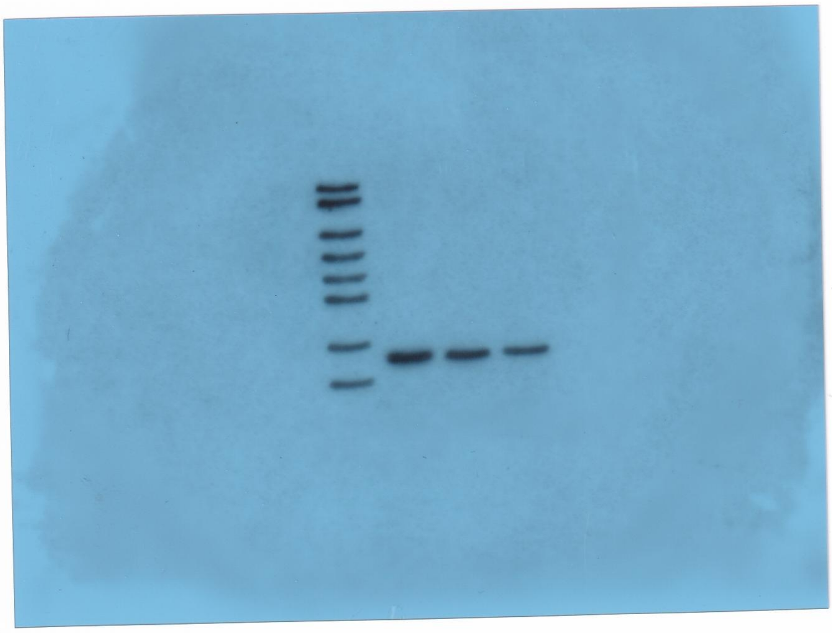

Fig S1A-2 Bax

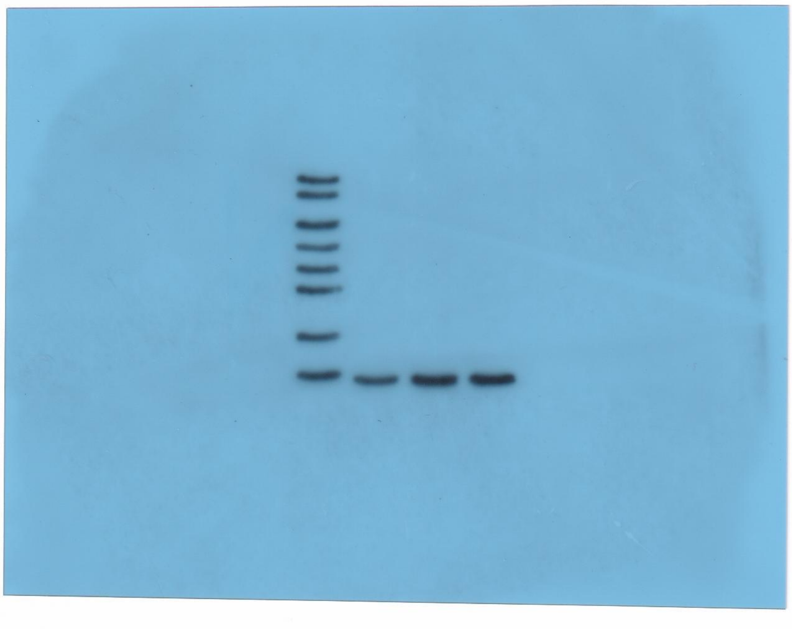

Fig S1A-3 Cleaved Caspase-3

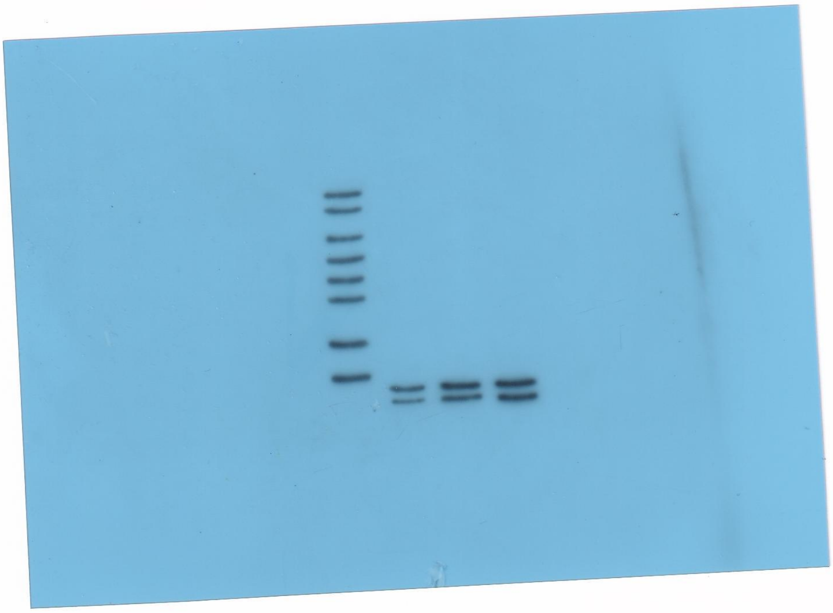

Fig S1A-4 β-actin

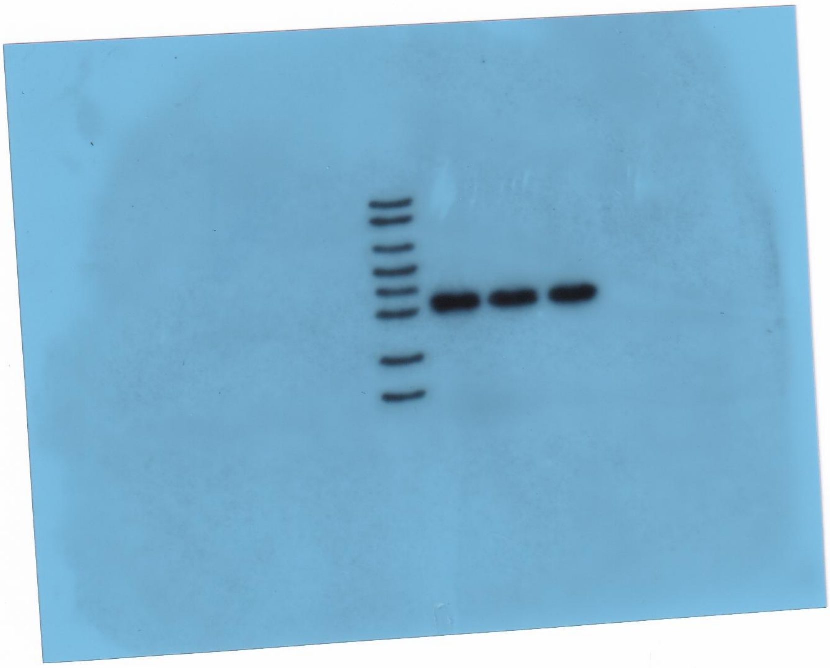

Fig S1A-5 Bcl-2

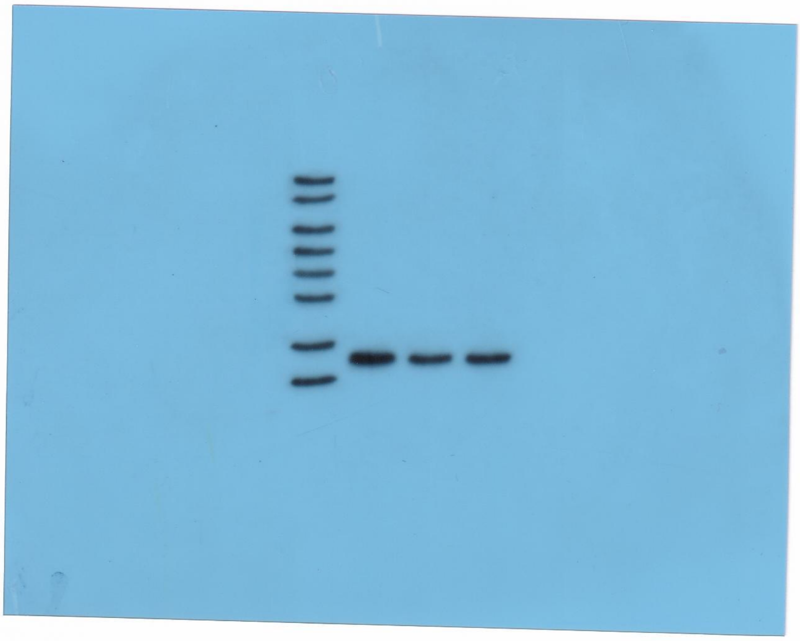

Fig S1A-6 Bax

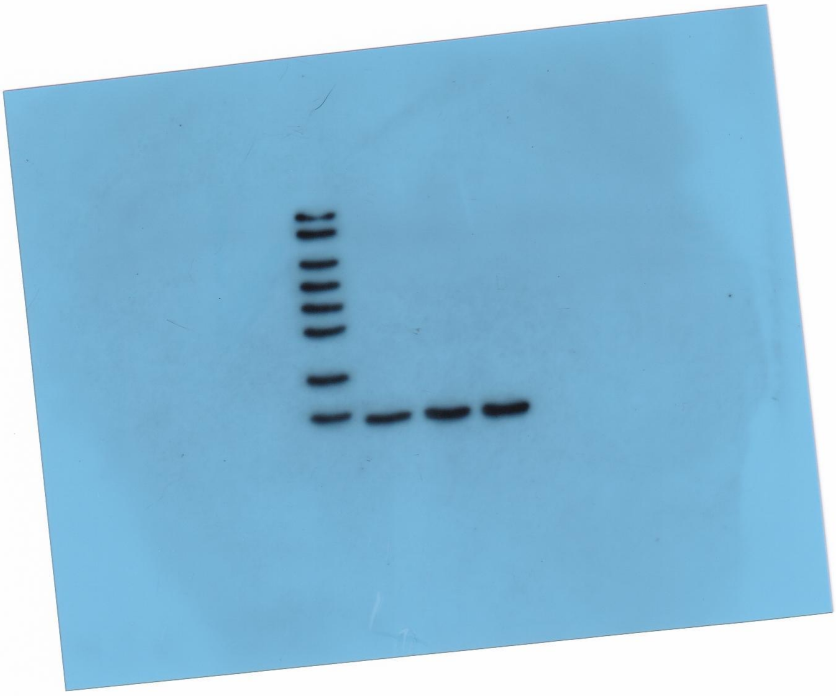

Fig S1A-7 Cleaved Caspase-3

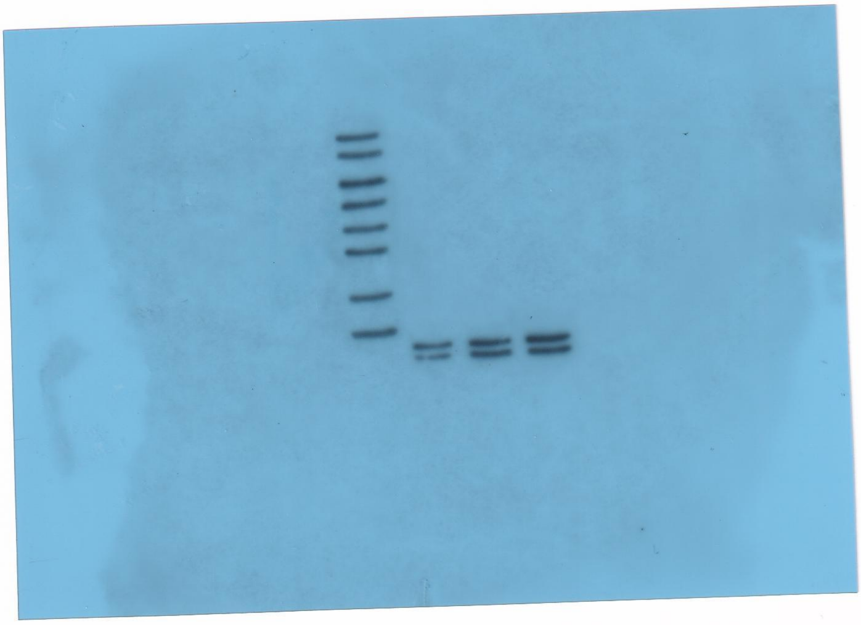

Fig S1A-8 β-actin

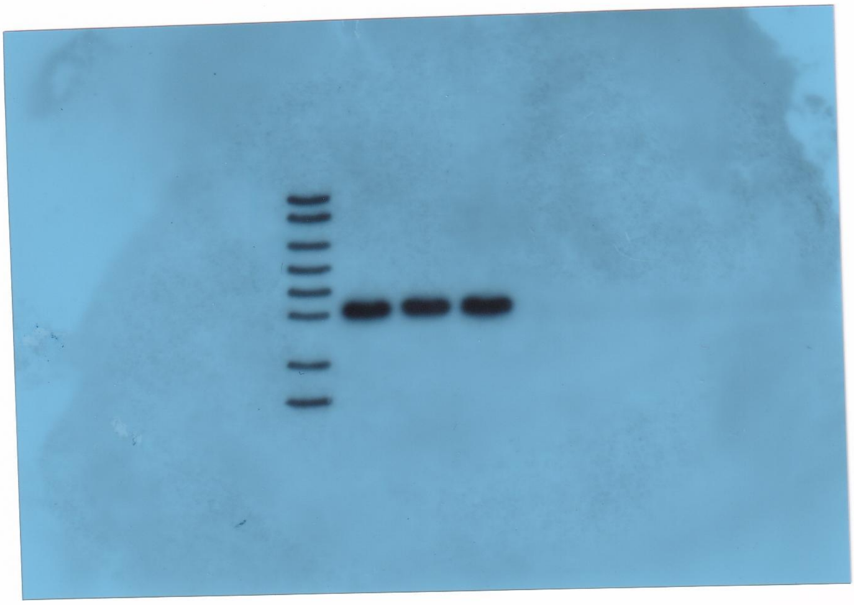

Fig S2D-1 INO80

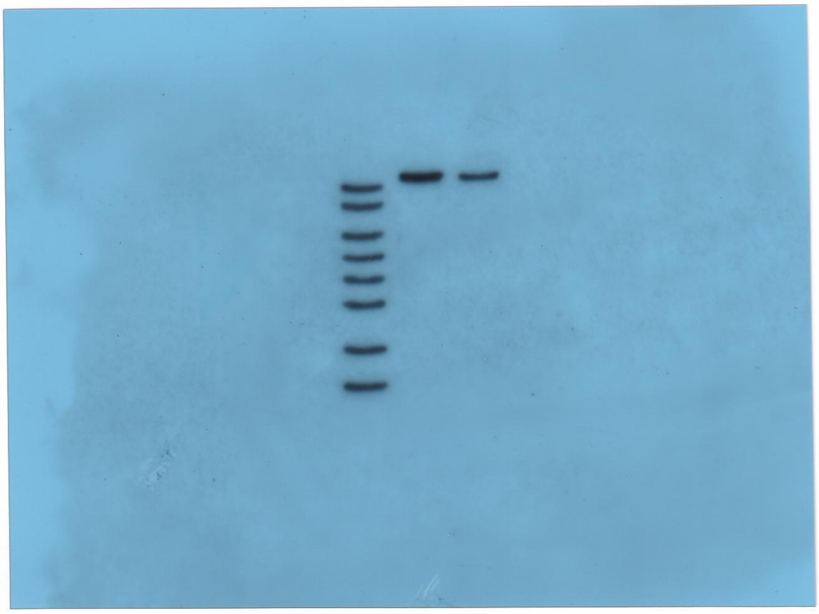

Fig S2D-2 β-actin

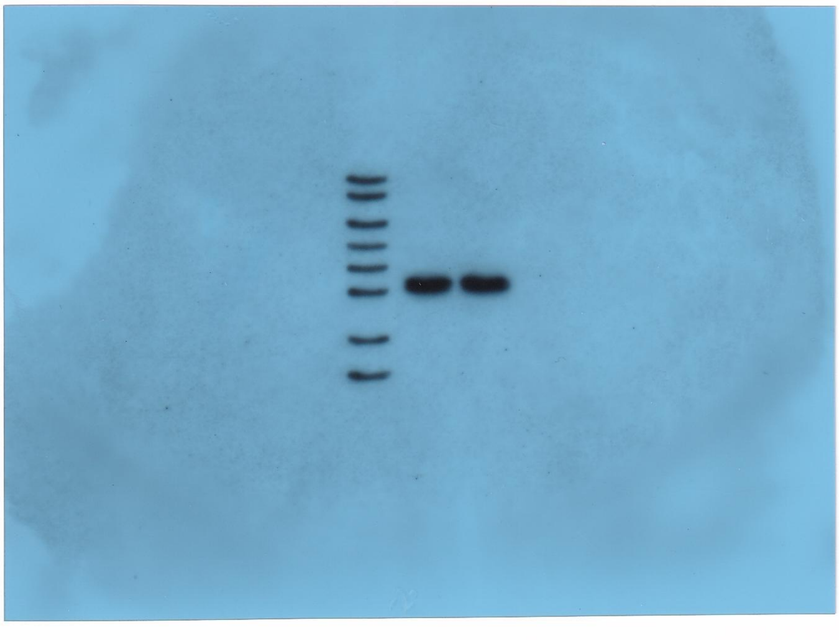

Fig S2D-3 INO80

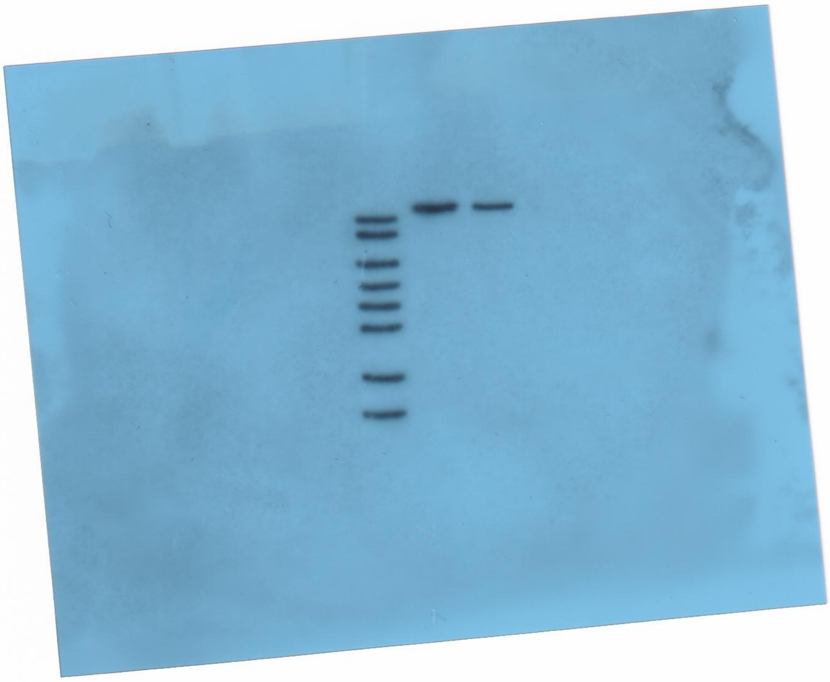

Fig S2D-4 β-actin

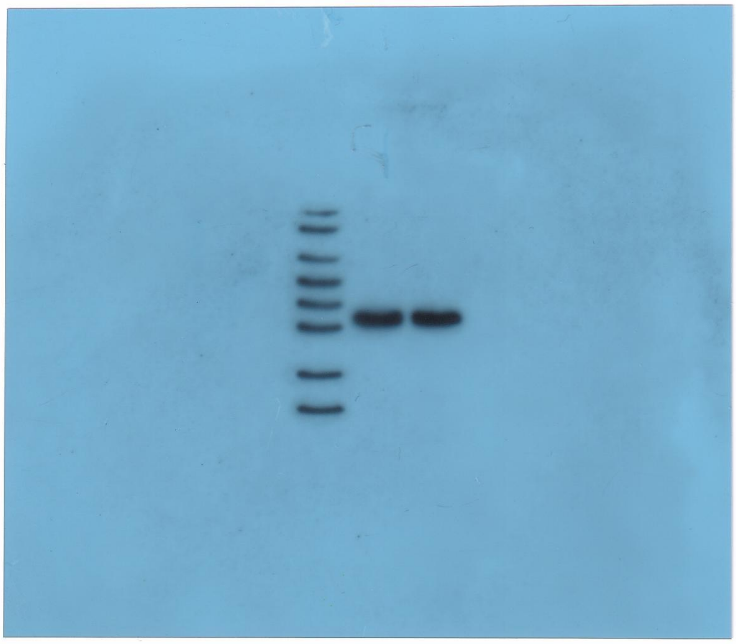

Fig S3F-1 DHX15

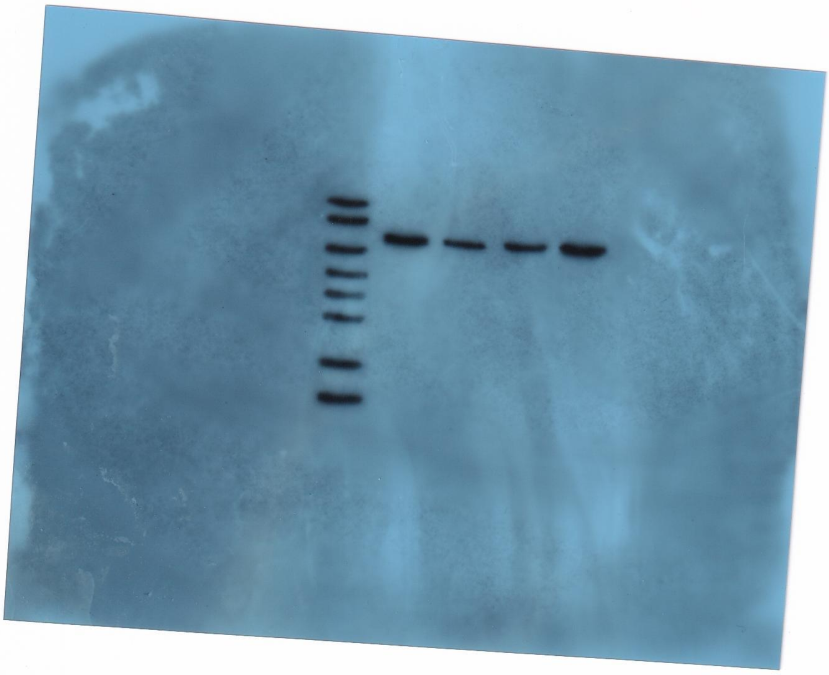

Fig S3F-2 β-actin

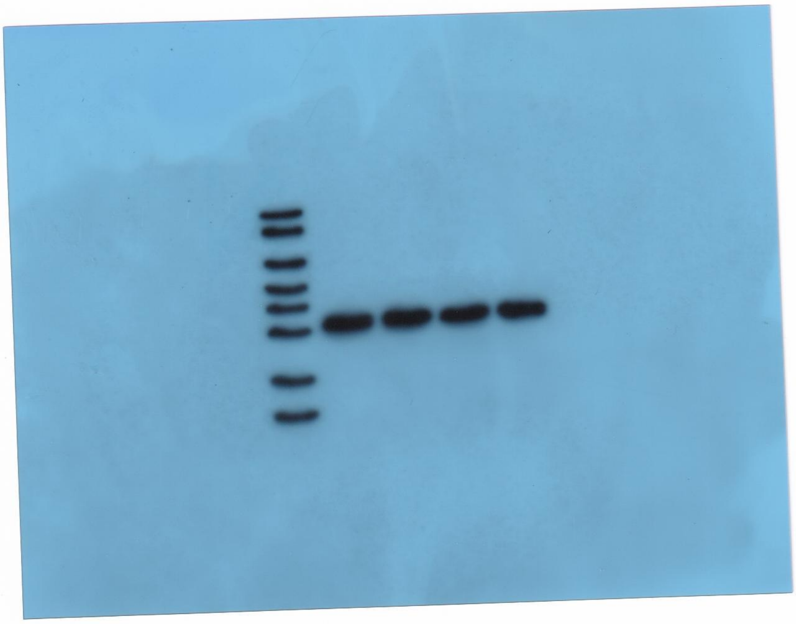

Fig S3F-3 DHX15

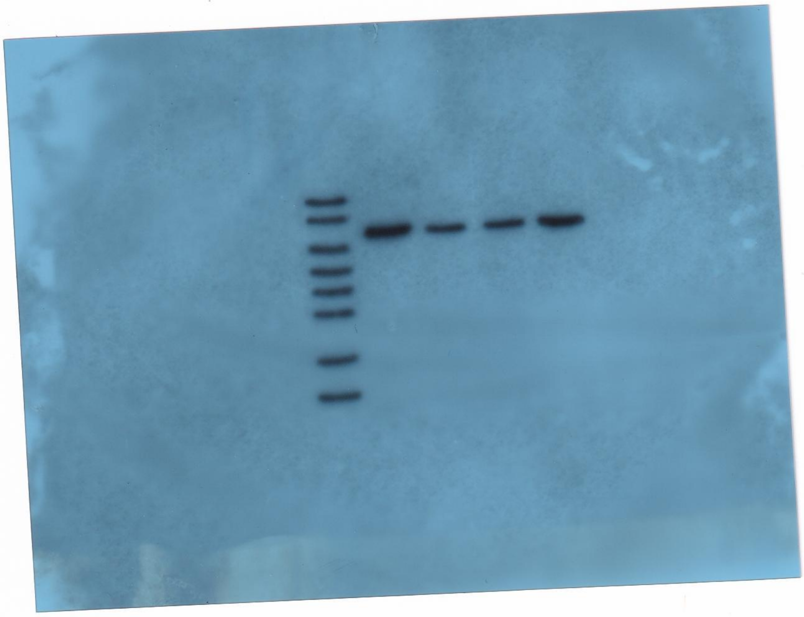

Fig S3F-4 β-actin

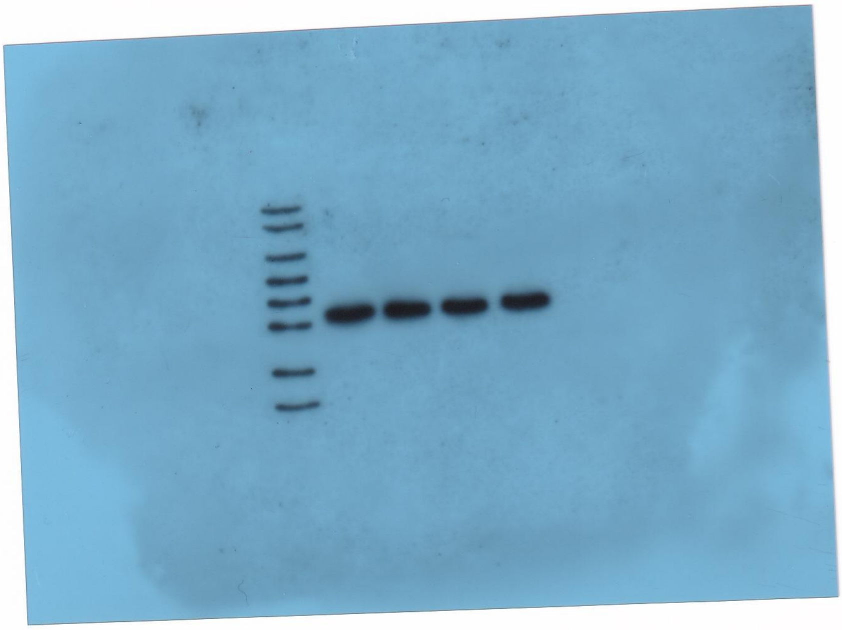

Fig S4A-1 INO80

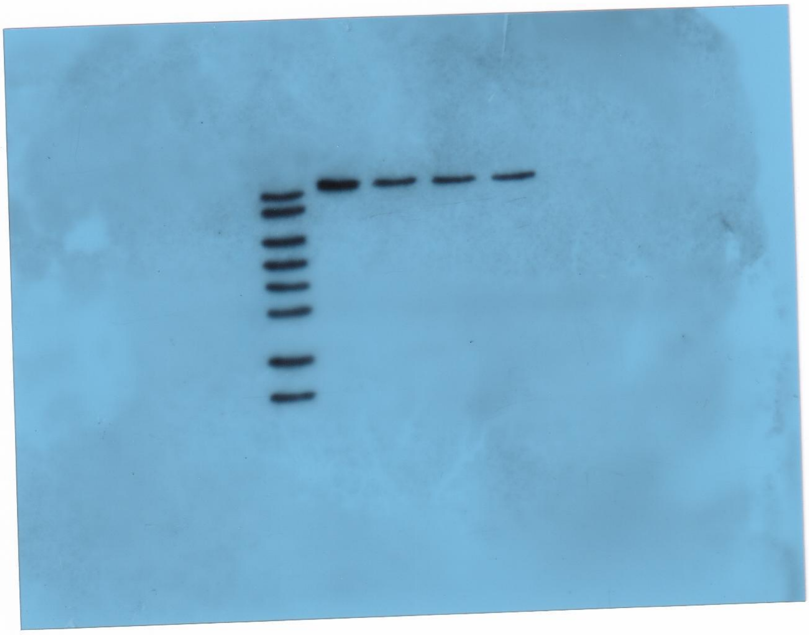

Fig S4A-2 DHX15

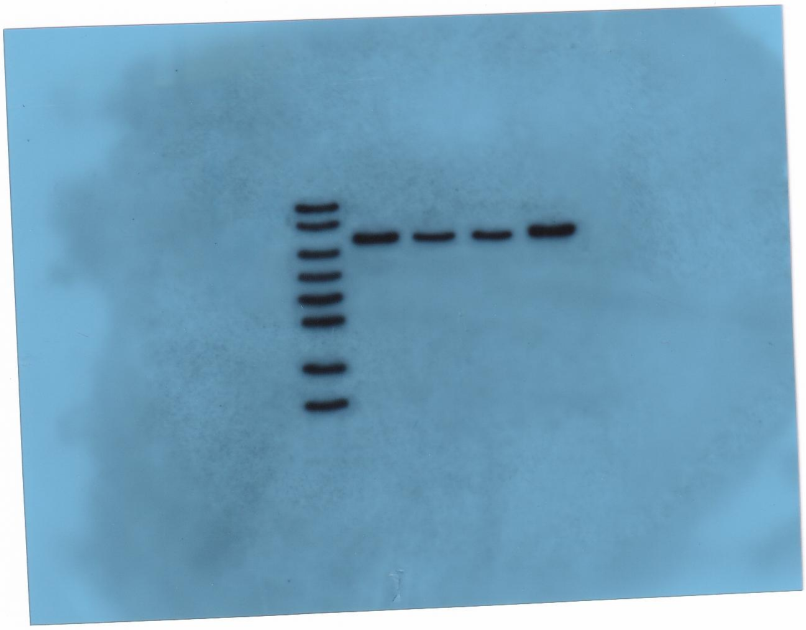

Fig S4A-3 β-actin

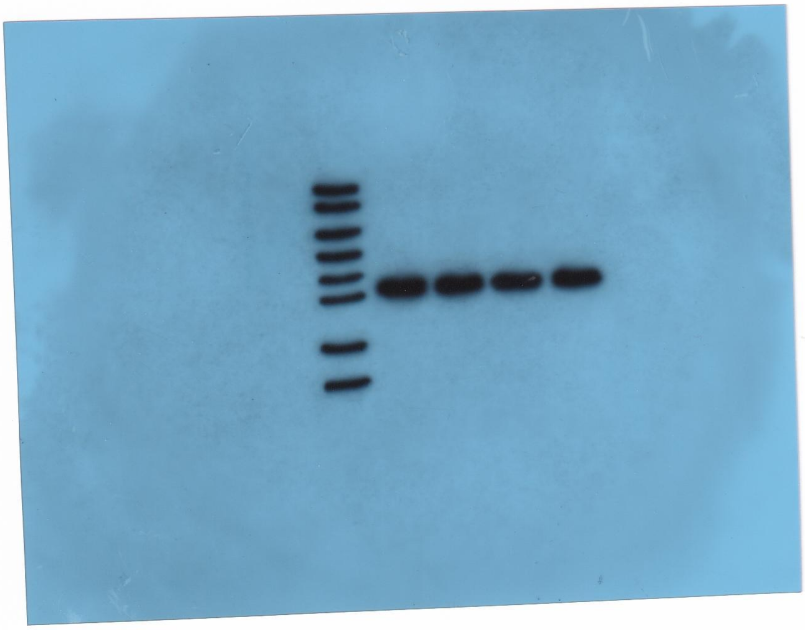

Fig S4A-4 INO80

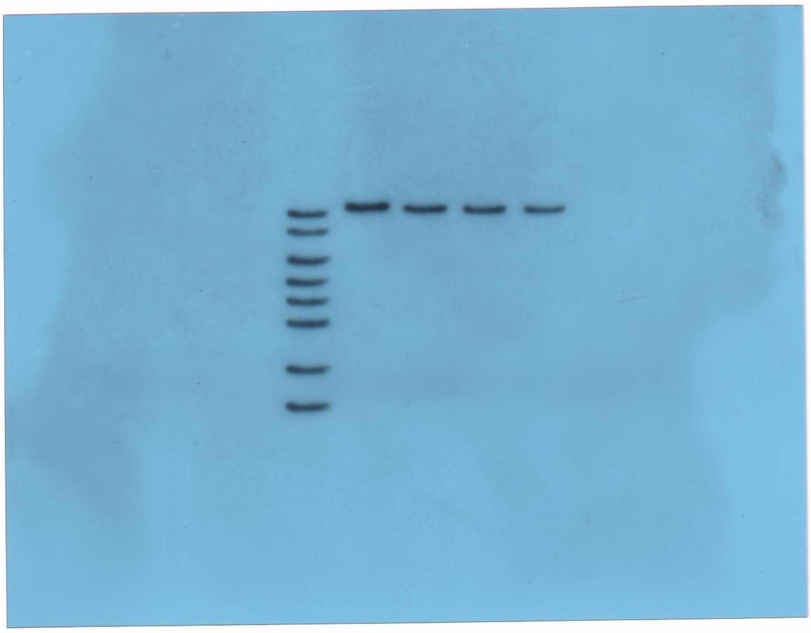

Fig S4A-5 DHX15

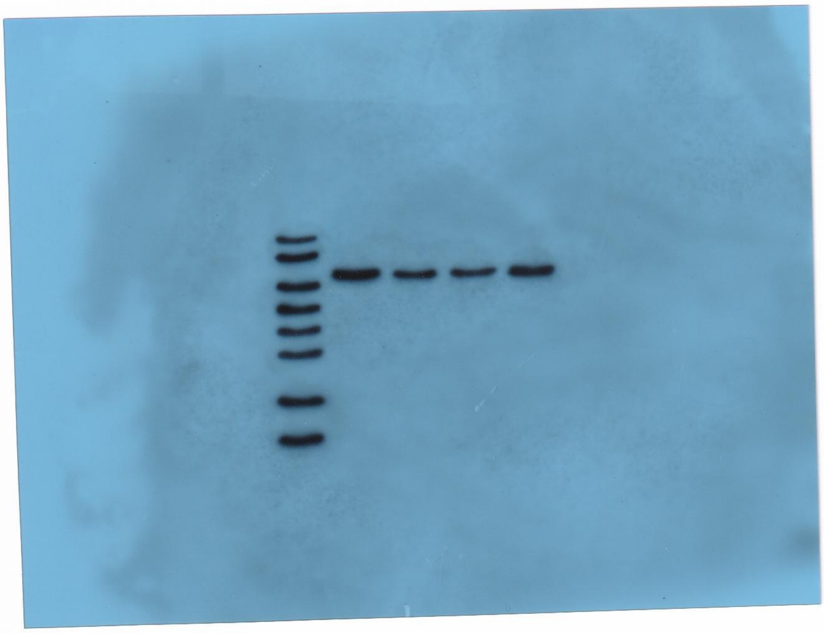

Fig S4A-6 β -actin

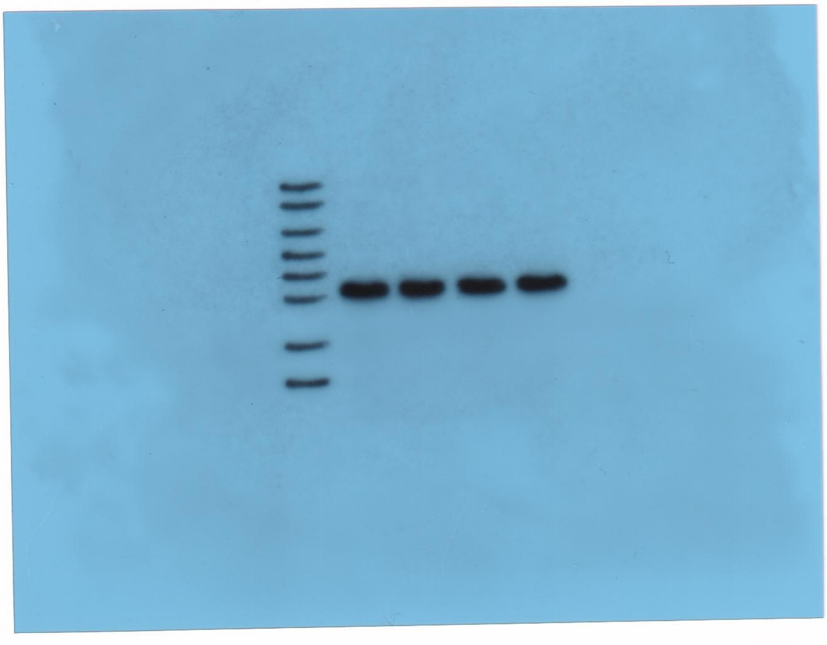

Fig S5B-1 Bcl-2

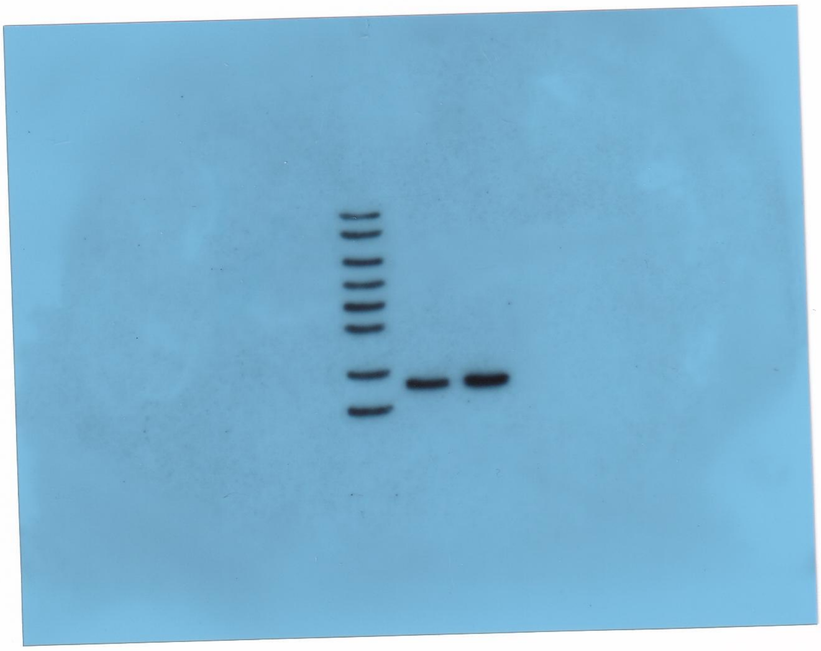

Fig S5B-2 Bax

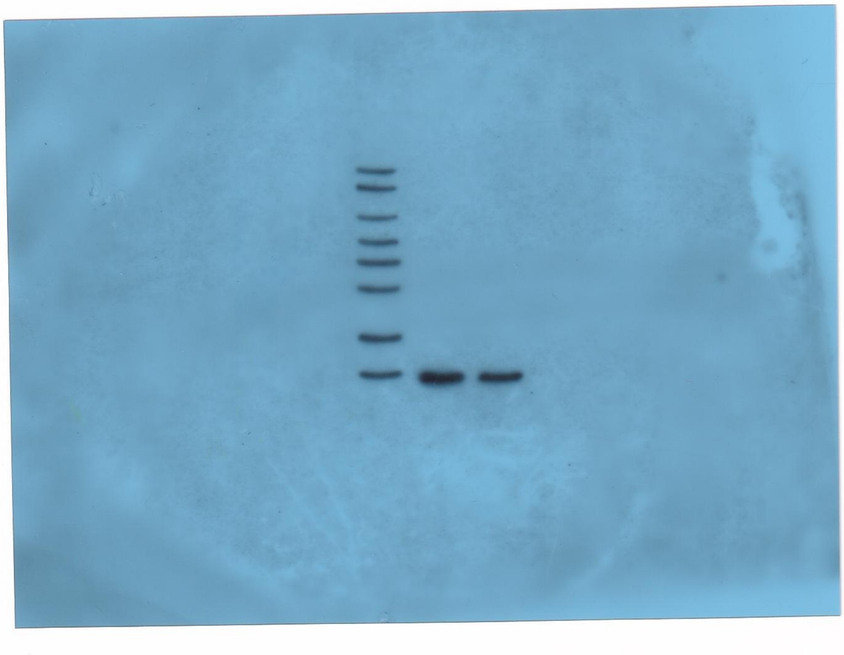

Fig S5B-3 Cleaved Caspase-3

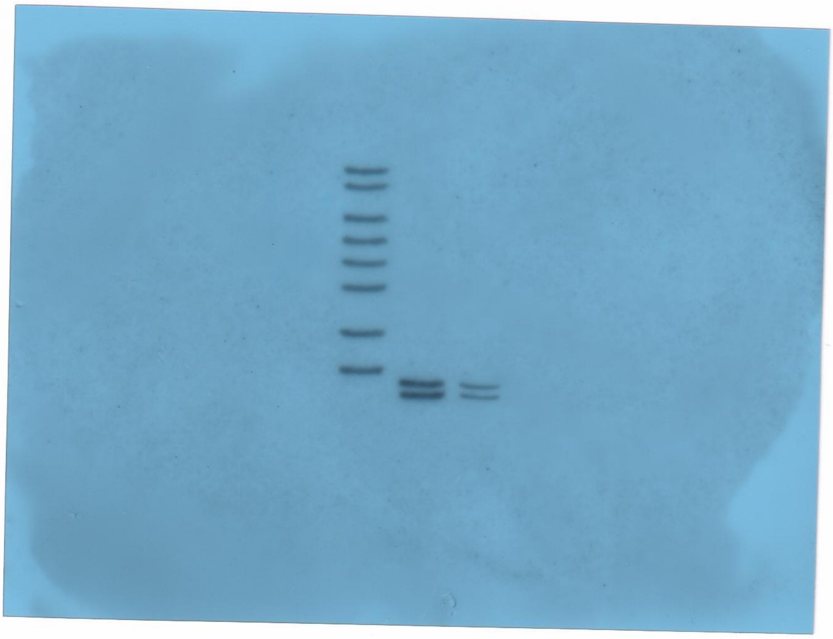

Fig S5B-4 β-actin

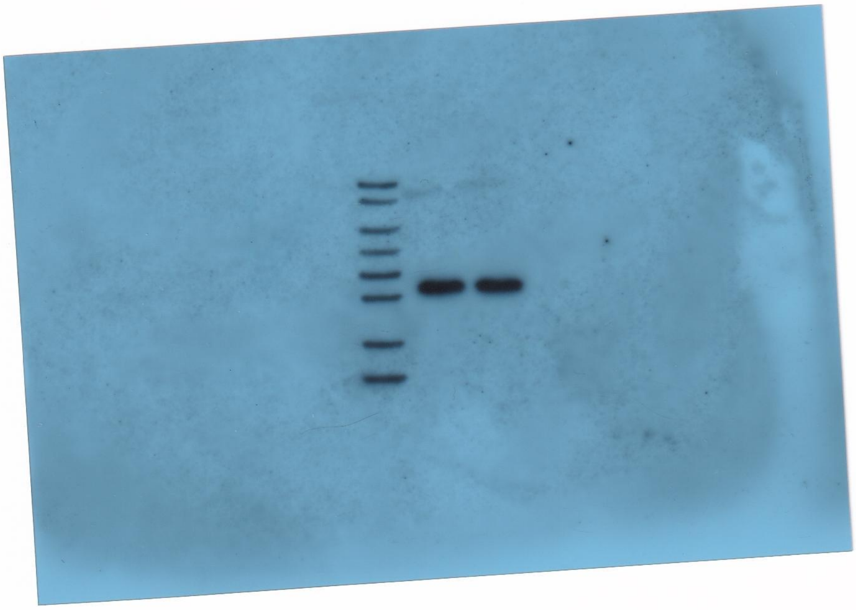

Fig S5B-5 Bcl-2

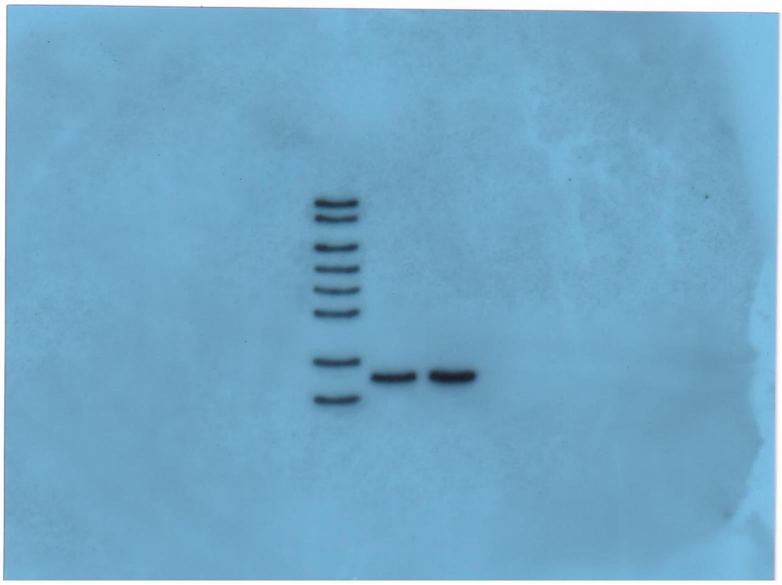

Fig S5B-6 Bax

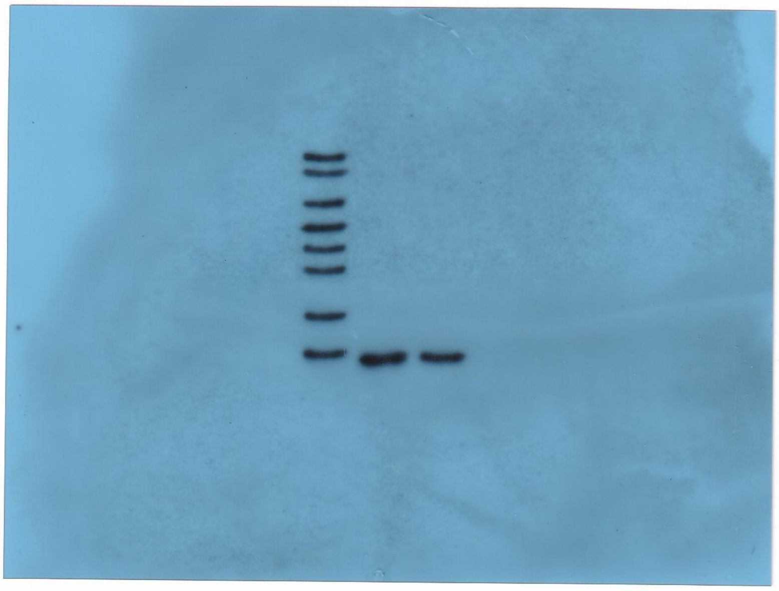

Fig S5B-7 Cleaved Caspase-3

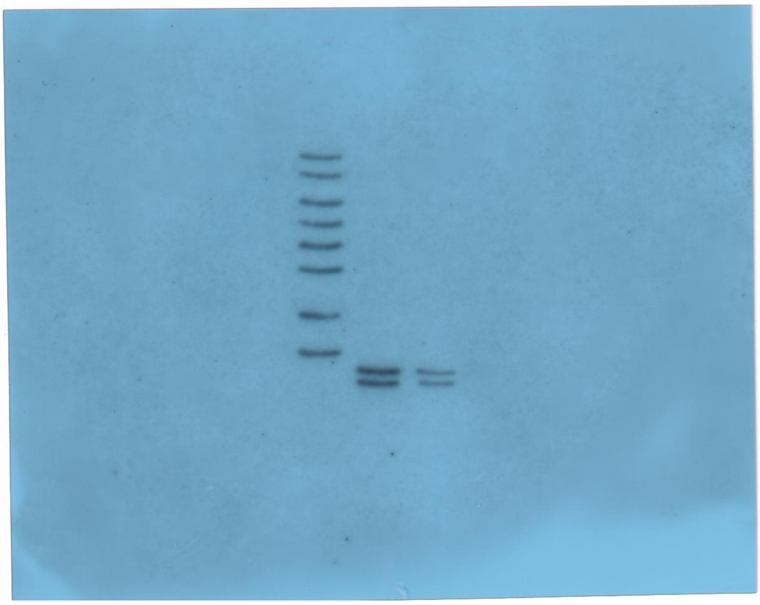

Fig S5B-8 β -actin

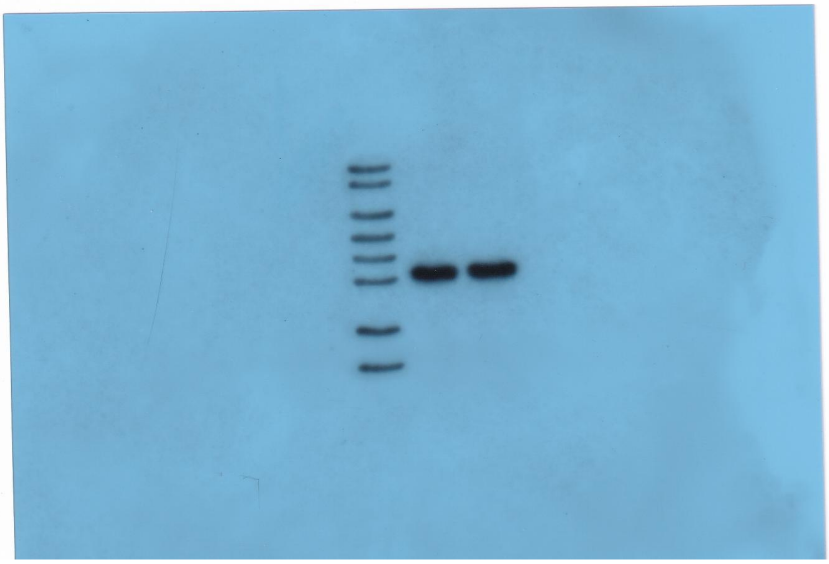

Supplement: Supplementary file 10 — Original western blots [file 41419_2025_7776_MOESM10_ESM.pdf]
